# Supplementary material for: Arylamines QSAR-Based Design and Molecular Dynamics of New Phenylthiophene and Benzimidazole Derivatives with Affinity for the C111, Y268, and H73 Sites of SARS-CoV-2 PLpro Enzyme
Source: Pharmaceuticals (Basel). 2024 May 9;17(5):606. doi: 10.3390/ph17050606 (PMC11124164; doi:10.3390/ph17050606)

Supplementary material

Arylamines QSAR-Based Design and Molecular Dynamics of New Phenylthiophene and Benzimidazole Derivatives with Affinity for the C111, Y268, and H73 Sites of SARS-CoV-2 PLpro Enzyme

Gianfranco Sabadini [1], Marco Mellado [2,*], César Morales [3] and Jaime Mella [1,4,*]

[1] Instituto de Química y Bioquímica, Facultad de Ciencias, Universidad de Valparaíso, Av. Gran Bretaña 1111, Valparaíso 2360102, Chile; gianfranco.sabadini@postgrado.uv.cl

[2] Instituto de Investigación y Postgrado, Facultad de Ciencias de la Salud, Universidad Central de Chile, Santiago 8330507, Chile

[3] Laboratorio de Materiales Funcionales, Centro Integrativo de Biología y Química Aplicada (CIBQA), Facultad de Ciencias de la Salud, Universidad Bernardo OHiggins, General Gana 1702, Santiago 8320000, Chile; cesar.morales@ubo.cl

[4] Centro de Investigación, Desarrollo e Innovación de Productos Bioactivos (CInBIO), Universidad de Valparaíso, Av. Gran Bretaña 1111, Valparaíso 2360102, Chile

* Correspondence: marco.mellado@ucentral.cl (M.M.); jaime.mella@uv.cl (J.M.)

**Table S1.** The experimental pIC_{50}, predictive pIC_{50} and residual values for the training and test set.	Pag. 2
**Table S2.** The SMILES of the entire data set used for the QSAR model.	Pag. 5
**Figure S1.** High res. Images of Docking and Molecular Dynamics	Pag. 8
**Figure S2.** The RMSD of the antivirals and designed molecules.	Pag. 52

Table S1. The experimental pIC_{50}, predictive pIC_{50} and residual values for the training and test set.

	pIC50			
	**Mol**	**Exp**	**Pred**	**Res**
	1	5.7932	5.2703	0.52
	2	5.9586	5.7228	0.24
	3	5.2596	5.5638	-0.30
	4	5.2218	5.6027	-0.38
	5	5.9957	5.6569	0.34
	6	6.2218	5.7595	0.46
	7	5.9208	5.5601	0.36
	8	6.0969	5.6826	0.41
	9	6.1549	5.8123	0.34
	10	5.7959	5.8425	-0.05
	11	5.3665	5.7334	-0.37
	12	5.6198	5.7446	-0.12
	13	5.4089	5.4229	-0.01
	14	5.8861	5.8705	0.02
*	15	5.4815	5.5415	-0.06
	16	5.2147	5.6203	-0.41
	17	4.9706	4.9969	-0.03
	18	5.4815	5.4849	0.00
	19	5.6198	5.9756	-0.36
	20	4.9626	5.2422	-0.28
*	21	5.7959	6.1161	-0.32
	22	5.7212	5.8610	-0.14
	23	5.7447	6.0421	-0.30
	24	5.5528	5.1787	0.37
	25	6.2291	5.9227	0.31
	26	5.8861	5.9265	-0.04
	27	5.7447	5.7750	-0.03
*	28	6.0458	6.3180	-0.27
	29	6.4089	6.4614	-0.05
	30	6.2518	6.4008	-0.15
	31	6.1249	6.0665	0.06
	32	6.0132	5.8246	0.19
	33	6.0915	5.7794	0.31
	34	6.0362	6.0576	-0.02
	35	6.0862	6.0020	0.08
	36	6.1938	6.3055	-0.11
*	37	6.1549	6.3037	-0.15
	38	6.4815	6.2844	0.20
	39	6.1938	6.3979	-0.20
	40	6.3872	6.4158	-0.03
	41	6.6778	6.3946	0.28
	42	6.3665	6.3969	-0.03

	43	6.9469	6.3889	0.56
*	44	6.6021	6.3989	0.20
*	45	6.0915	5.9576	0.13
	46	5.7447	5.8732	-0.13
*	47	5.9586	5.9038	0.05
	48	5.6383	5.6343	0.00
	49	5.8539	5.9360	-0.08
	50	5.5436	5.1288	0.41
	51	5.1612	5.1707	-0.01
	52	4.8239	4.4353	0.39
*	53	5.1249	5.2728	-0.15
	54	5.0000	5.4479	-0.45
	55	5.0000	5.1307	-0.13
	56	5.1192	5.3934	-0.27
*	57	5.2441	5.4936	-0.25
	58	4.8861	4.7325	0.15
	59	4.3645	4.6669	-0.30
	60	4.5258	4.6212	-0.10
	61	4.5058	4.6240	-0.12
	62	4.4584	4.5846	-0.13
*	63	4.4389	4.7455	-0.31
*	64	5.0223	4.6103	0.41
	65	4.5800	4.5345	0.05
	66	4.6421	4.5707	0.07
*	67	4.1209	4.0921	0.03
	68	4.0400	4.1036	-0.06
	69	6.1938	6.3859	-0.19
*	70	6.1675	5.7847	0.38
*	71	6.2076	6.1755	0.03
	72	5.7305	5.6786	0.05
	73	5.4841	5.6207	-0.14
	74	5.6126	5.6438	-0.03
*	75	5.5918	5.1286	0.46
	76	5.6576	5.2757	0.38
	77	3.9031	4.1783	-0.28
*	78	4.2157	4.3637	-0.15
*	79	5.6073	5.0538	0.55
	80	5.2924	5.3276	-0.04
	81	5.1938	5.3670	-0.17
	82	5.1549	5.2915	-0.14
*	83	4.7773	4.8182	-0.04
	84	4.7077	4.5312	0.18
	85	4.7905	4.5555	0.23
	86	4.8928	5.0501	-0.16
	87	4.6253	4.7200	-0.09
	88	4.7595	4.7559	0.00

	89	4.6882	4.6716	0.02
	90	4.4123	4.6861	-0.27
	91	4.7160	5.1436	-0.43
	92	5.1487	5.4517	-0.30
*	93	4.9314	5.3882	-0.46
	94	5.2899	5.8119	-0.52
	95	6.2076	5.7904	0.42
	96	5.8013	5.7898	0.01
*	97	5.8861	5.7878	0.10
	98	5.6073	5.8467	-0.24
	99	6.1805	5.8153	0.37
	100	6.1739	5.8121	0.36
	101	5.9393	5.8987	0.04
*	102	5.3990	5.6804	-0.28
	103	5.8861	5.9937	-0.11
	104	5.1878	5.2057	-0.02
	105	5.0511	5.1481	-0.10
	106	5.5654	5.1510	0.41
	107	5.2596	5.3131	-0.05
	108	5.2132	5.3635	-0.15
	109	6.0506	5.6907	0.36
	110	5.3546	5.4823	-0.13
*	111	5.8416	5.4159	0.43
	112	5.0353	5.6163	-0.58
*	113	5.0888	5.1672	-0.08

* Test set compound

Table S2. The SMILES of the entire data set used for the QSAR model.

Molecule	pIC_{50} Exp	SMILES
1	5.7932	CC(c1cccc2ccccc12)NC(c1c(C)ccc(N)c1)=O
2	5.9586	CC(c1cccc2ccccc12)NC(c1c(C)ccc(NC(CC2)CCN2C(OC(C)(C)C)=O)c1)=O
3	5.2596	CC(c1cccc2ccccc12)NC(c1c(C)ccc(NC(C(C2)CN2C(OC(C)(C)C)=O)=O)c1)=O
4	5.2218	CC(c1cccc2ccccc12)NC(c1c(C)ccc(NC(C(CC2)CCN2C(OC(C)(C)C)=O)=O)c1)=O
5	5.9957	CC(c1cccc2ccccc12)NC(c1c(C)ccc(NC2CNC2)c1)=O
6	6.2218	CC(c1cccc2ccccc12)NC(c1c(C)ccc(NC2CCNCC2)c1)=O
7	5.9208	CC(c1cccc2ccccc12)NC(c1c(C)ccc(NC(C2CNC2)=O)c1)=O
8	6.0969	CC(c1cccc2ccccc12)NC(c1c(C)ccc(NC(C2CCNCC2)=O)c1)=O
9	6.1549	CC(c1cccc2ccccc12)NC(c1c(C)ccc(NC2CCN(C)CC2)c1)=O
10	5.7959	CC(c1cccc2ccccc12)NC(c1c(C)ccc(N(C)C2CN(C)C2)c1)=O
11	5.3665	CC(c1cccc2ccccc12)NC(c1c(C)ccc(NC2CN(C)C2)c1)=O
12	5.6198	CC(c1cccc2ccccc12)NC(c1c(C)ccc(N(C)C2CNC2)c1)=O
13	5.4089	CC(c1cccc2ccccc12)NC(c1c(C)ccc(CNCC(O)=O)c1)=O
14	5.8861	CC(c1cccc2ccccc12)NC(c1c(C)ccc(CNC2CNC2)c1)=O
15	5.4815	CC(c1cccc2ccccc12)NC(c1c(C)ccc(CN(C2)CC2C(O)=O)c1)=O
16	5.2147	CC(c1cccc2ccccc12)NC(c1c(C)ccc(CN(CC2)CCC2C(O)=O)c1)=O
17	4.9706	CC(c1cccc2ccccc12)NC(c(cc(cc1)N(C)C2CNC2)c1Cl)=O
18	5.4815	CC(c1csc2c1cccc2)NC(c1c(C)ccc(N)c1)=O
19	5.6198	CC(c1csc2c1cccc2)NC(c1c(C)ccc(N(C)C2CNC2)c1)=O
20	4.9626	CC(c1csc2c1cccc2)NC(c(cc(cc1)N(C)C2CNC2)c1Cl)=O
21	5.7959	CC(c1c(c(cccc2)c2[nH]2)c2ccc1)NC(c1c(C)ccc(N(C)C2CNC2)c1)=O
22	5.7212	CC(c1csc2c1cccc2)NC(c1c(C)ccc(NC2CNC2)c1)=O
23	5.7447	CC(c1c(c(cccc2)c2[nH]2)c2ccc1)NC(c1c(C)ccc(NC2CNC2)c1)=O
24	5.5528	CC(c1cc(-c2cccs2)ccc1)NC(c(cc(cc1)NC2CNC2)c1Cl)=O
25	6.2291	CC(c1cccc(-c2cccs2)c1)NC(c1c(C)ccc(NC2CNC2)c1)=O
26	5.8861	CC(c1cccc(-c2cscc2)c1)NC(c1c(C)ccc(NC2CNC2)c1)=O
27	5.7447	CC(c1cc(-c2c[nH]cc2)ccc1)NC(c1c(C)ccc(NC2CNC2)c1)=O
28	6.0458	CC(c1cccc(-c2ccc(CN3CCOCC3)s2)c1)NC(c1c(C)ccc(NC2CNC2)c1)=O
29	6.4089	CC(c1cccc(-c2ccc(CNC3CCCC3)s2)c1)NC(c1c(C)ccc(NC2CNC2)c1)=O
30	6.2518	CC(c1cccc(-c2ccc(CN3CCCC3)s2)c1)NC(c1c(C)ccc(NC2CNC2)c1)=O
31	6.1249	CC(c1cccc(-c2ccc(C)s2)c1)NC(c1c(C)ccc(NC2CNC2)c1)=O
32	6.0132	CC(c1cccc(-c2ccc(C(O)=O)s2)c1)NC(c1c(C)ccc(NC2CNC2)c1)=O
33	6.0915	CC(c1cccc(-c2ccc(C(OC)=O)s2)c1)NC(c1c(C)ccc(NC2CNC2)c1)=O
34	6.0362	CC(c1cccc(-c2ccc(C(NCC3OCCC3)=O)s2)c1)NC(c1c(C)ccc(NC2CNC2)c1)=O
35	6.0862	CC(c1cccc(-c2ccc(C(NCC3OCC3)=O)s2)c1)NC(c1c(C)ccc(NC2CNC2)c1)=O
36	6.1938	CC(c1cccc(-c2ccc(CN(CC3)CC3O)s2)c1)NC(c1c(C)ccc(NC2CNC2)c1)=O
37	6.1549	CC(c1cccc(-c2ccc(CN(CC3)CC3O)s2)c1)NC(c1c(C)ccc(NC2CNC2)c1)=O
38	6.4815	CC(c1cccc(-c2ccc(CNC3COCC3)s2)c1)NC(c1c(C)ccc(NC2CNC2)c1)=O
39	6.1938	CC(c1cccc(-c2ccc(CNC(CCN3)C3=O)s2)c1)NC(c1c(C)ccc(NC2CNC2)c1)=O
40	6.3872	CC(c1cccc(-c2ccc(CNC(C3)CNC3=O)s2)c1)NC(c1c(C)ccc(NC2CNC2)c1)=O
41	6.6778	CC(c1cccc(-c2ccc(CNC(CC3)CC3O)s2)c1)NC(c1c(C)ccc(NC2CNC2)c1)=O
42	6.3665	CC(c1cccc(-c2ccc(CNC(CC3)CC3O)s2)c1)NC(c1c(C)ccc(NC2CNC2)c1)=O
43	6.9469	CC(c1cccc(-c2ccc(CNC(CC3)CC3O)s2)c1)NC(c1c(C)ccc(NC2CNC2)c1)=O

44	6.6021	CC(c1cccc(-c2ccc(CNC(CC3)CC3O)s2)c1)NC(c1c(C)ccc(NC2CNC2)c1)=O
45	6.0915	CC(c1cccc(-c2ccc(CNC(OC(C)(C)C)=O)s2)c1)NC(c1c(C)ccc(N)c1)=O
46	5.7447	CC(c1cccc(-c2ccc(CN)s2)c1)NC(c1c(C)ccc(N)c1)=O
47	5.9586	CC(c1cccc(-c2ccc(CNC(C3CCCC3)=O)s2)c1)NC(c1c(C)ccc(N)c1)=O
48	5.6383	CC(c1cccc(-c2ccc(CNC(C)=O)s2)c1)NC(c1c(C)ccc(NC(C)=O)c1)=O
49	5.8539	CC(c1cccc(-c2ccc(CNC(CC3)CC3O)s2)c1)NC(c1c(C)ccc(NC(C)=O)c1)=O
50	5.5436	O=C1N(c2ccccc2)[Se]c2c1cccc2
51	5.1612	CC(c1cccc2ccccc12)NC(c1c(C)cccc1)=O
52	4.8239	CC(c1cccc2ccccc12)NC(c(cccc1)c1Cl)=O
53	5.1249	CC(c1cccc2ccccc12)NC(c(cc1)c(C)cc1N)=O
54	5.0000	CC(c1cccc2ccccc12)NC(c1c(C)ccc(N(C)C)c1)=O
55	5.0000	CC(c1cccc2ccccc12)NC(c(cc1)c(C)cc1NS(C)(=O)=O)=O
56	5.1192	CC(c1cccc2ccccc12)N(C1)Cc2c1cccc2
57	5.2441	CC(c1cccc2ccccc12)N(C1)Cc2c1ccc(N)c2
58	4.8861	Oc(cc1)ccc1C(Oc1c2c(O)cc(O)c1-c(cc(cc1)C(Oc3c4c(O)cc(O)c3)=CC4=O)c1O)=CC2=O
59	4.3645	COc(cc1)ccc1C(Oc1c2c(O)cc(O)c1-c(cc(cc1)C(Oc3c4c(O)cc(O)c3)=CC4=O)c1O)=CC2=O
60	4.5258	COc(cc1O)cc(OC(c(cc2)cc(-c(c(OC(c(cc3)ccc3O)=C3)c(c(O)c4)C3=O)c4O)c2OC)=C2)c1C2=O
61	4.5058	COc(cc1)ccc1C(Oc1c2c(O)cc(O)c1-c(cc(cc1)C(Oc3c4c(O)cc(O)c3)=CC4=O)c1OC)=CC2=O
62	4.4584	COc(cc1)ccc1C(Oc1c2c(O)cc(O)c1-c(cc(cc1)C(Oc3c4c(O)cc(OC)c3)=CC4=O)c1OC)=CC2=O
63	4.4389	Oc1ccc(C(C(c(c(OC(c(cc2)cc(O)c2O)=C2)c(c(O)c3)C2=O)c3O)C2=O)Oc3c2c(O)cc(O)c3)cc1
64	5.0223	Oc(cc1)ccc1C(Oc(c1c2O)cc(O)c2Oc(cc2)ccc2C(Oc2c3c(O)cc(O)c2)=CC3=O)=CC1=O
65	4.5800	COc(cc1)ccc1C(Oc(c1c2O)cc(OC)c2Oc(cc2)ccc2C(Oc2c3c(O)cc(O)c2)=CC3=O)=CC1=O
66	4.6421	COc(ccc(C(Oc1c2c(O)cc(O)c1)=CC2=O)c1)c1Oc(cc1)ccc1C(Oc1c2c(O)cc(O)c1)=CC2=O
67	4.1209	Oc(cc1)ccc1C(Oc1c2c(O)cc(O)c1)=CC2=O
68	4.0400	COc(cc1)ccc1C(Oc1c2c(O)cc(O)c1)=CC2=O
69	6.1938	CCOC(SSC(OCC)=S)=S
70	6.1675	S=C(N1CCCC1)SSC(N1CCCC1)=S
71	6.2076	CN(C)C(SSC(N(C)C)=S)=S
72	5.7305	COc(nc12)ccc2nc(cc(cc2)Cl)c2c1Nc(cc1CN2CCCC2)cc(CN2CCCC2)c1O
73	5.4841	CN(C)Cc1cc(Nc(c2c3)c(ccc(Cl)c4)c4nc2ccc3OC)cc(CN(C)C)c1O
74	5.6126	COc(cc12)ccc2nc(cc(cc2)Cl)c2c1Nc(cc1CN2CCCC2)cc(CN2CCCC2)c1O
75	5.5918	COc(cc12)ccc2nc(cc(cc2)Cl)c2c1Nc(cc1)cc(CN2CCCC2)c1O
76	5.6576	CC(c1cc2ccccc2cc1)NC(c1c(C)ccc(N)c1)=O
77	3.9031	OCC(C(C(C1O)O)O)OC1Oc1cc(O)cc(/C=C/c(cc2)ccc2O)c1
78	4.2157	Oc1ccc(/C=C/c2cc(O)cc(O)c2)cc1
79	5.6073	Cc1[n+](Cc2nccnc2)c(C(c(cccc2)c2C2=O)=O)c2n1CCOC.[Br-]
80	5.2924	CC(c1cccc2ccccc12)NC(c1c(C)ccc(NC(NC(N)=O)=O)c1)=O
81	5.1938	CC(c1cccc2ccccc12)NC(c1c(C)ccc(NC(C=C)=O)c1)=O
82	5.1549	CC(c1cccc2ccccc12)NC(c1c(C)ccc(NC(Oc(cc2)ccc2[N+]([O-])=O)=O)c1)=O
83	4.7773	CC(C)c1c(CNCc2cc(C(O)=O)c(C)o2)occ1
84	4.7077	OC(CCN(C1CCOCC1)C(c(cc1)ccc1OC1CCCC1)=O)=O
85	4.7905	OC(CCN(C1CCOCC1)C(CCc1ncc(-c(cccc2)c2F)o1)=O)=O
86	4.8928	Cc1nsc(NC)c1C(Nc(cc1)cc(CC2)c1N(Cc(cc1)ccc1Cl)C2=O)=O
87	4.6253	OC(CN(Cc1ccccc1)C(c1ccc(CN(Cc2ccccc2)S(c(cc2)ccc2Cl)(=O)=O)cc1)=O)=O
88	4.7595	OC(C(Cc1ccccc1)NC(c1ccc(CN(Cc2ccccc2)S(c(cc2)ccc2Cl)(=O)=O)cc1)=O)=O
89	4.6882	OC(C(c1ccccc1)NC(c1ccc(CN(Cc2ccccc2)S(c(cc2)ccc2Cl)(=O)=O)cc1)=O)=O

90	4.4123	CC(C)(Cc(cc1)ccc1-c(cc1)cc(C(F)(F)F)c1Cl)C(O)=O
91	4.7160	CC(c1cccc2ccccc12)N(C)CC(C(N1)=O)=CNC1=O
92	5.1487	CC(c1cccc2ccccc12)N(C)Cc1ccc[nH]1
93	4.9314	CC(c1cccc2ccccc12)N(C)Cc(cc1)cnc1OC
94	5.2899	CC(c1cccc2ccccc12)N(C)Cc1ccc2ncccc2c1
95	6.2076	CC(c1cccc2ccccc12)N(C)Cc1c(cc[nH]2)c2ccc1
96	5.8013	CC(c1cccc2ccccc12)N(C)Cc(cc1)cc2c1[nH]cc2
97	5.8861	CC(c1cccc2ccccc12)N(C)Cc1cc([nH]cc2)c2cc1
98	5.6073	CC(c1cccc2ccccc12)N(C)Cc(cc1)cc2c1[nH]nn2
99	6.1805	CC(c1cccc2ccccc12)N(C)Cc(cc1)cc2c1[nH]nc2
100	6.1739	CC(c1cccc2ccccc12)N(C)Cc1n[nH]c2c1cccc2
101	5.9393	CC(c1cccc2ccccc12)N(C)Cc1nn(CC(N)=O)c2c1cccc2
102	5.3990	CC(c1cccc2ccccc12)N(C)Cc1n[nH]c(cc2)c1cc2O
103	5.8861	CC(c1cccc2ccccc12)N(C)Cc1ccc(B(O)O)cc1
104	5.1878	CC(c1cccc2ccccc12)N(C)Cc(cc1F)cc(F)c1O
105	5.0511	CC(c1cccc2ccccc12)N(C)Cc(cc1)cc(O)c1O
106	5.5654	CC(c1cccc2ccccc12)N(C)Cc1cc(O)cc(O)c1
107	5.2596	CC(c1cccc2ccccc12)N(C)Cc(cc1)ncc1O
108	5.2132	CC(c1cccc2ccccc12)N(C)Cc1nccc(S(N)(=O)=O)c1
109	6.0506	CC(c1cccc2ccccc12)N(C)Cc1c(C)ccc(N)c1
110	5.3546	CC(c1cccc2ccccc12)N(C)Cc(cc1)ccc1NC(C)=O
111	5.8416	CC(c1cccc2ccccc12)N(C)Cc(c(F)cc(N)c1)c1F
112	5.0353	CC(c1cccc2ccccc12)N(C)Cc(cc1)ccc1N(C)CCO
113	5.0888	O=C1C2=C(N=C(C3=CN=CC=C3)O2)C(C4=CC=CC=C41)=O

Figure S1. High res. Images of Docking and Molecular Dynamics

Indinavir in Y268 (State of protonation 1):

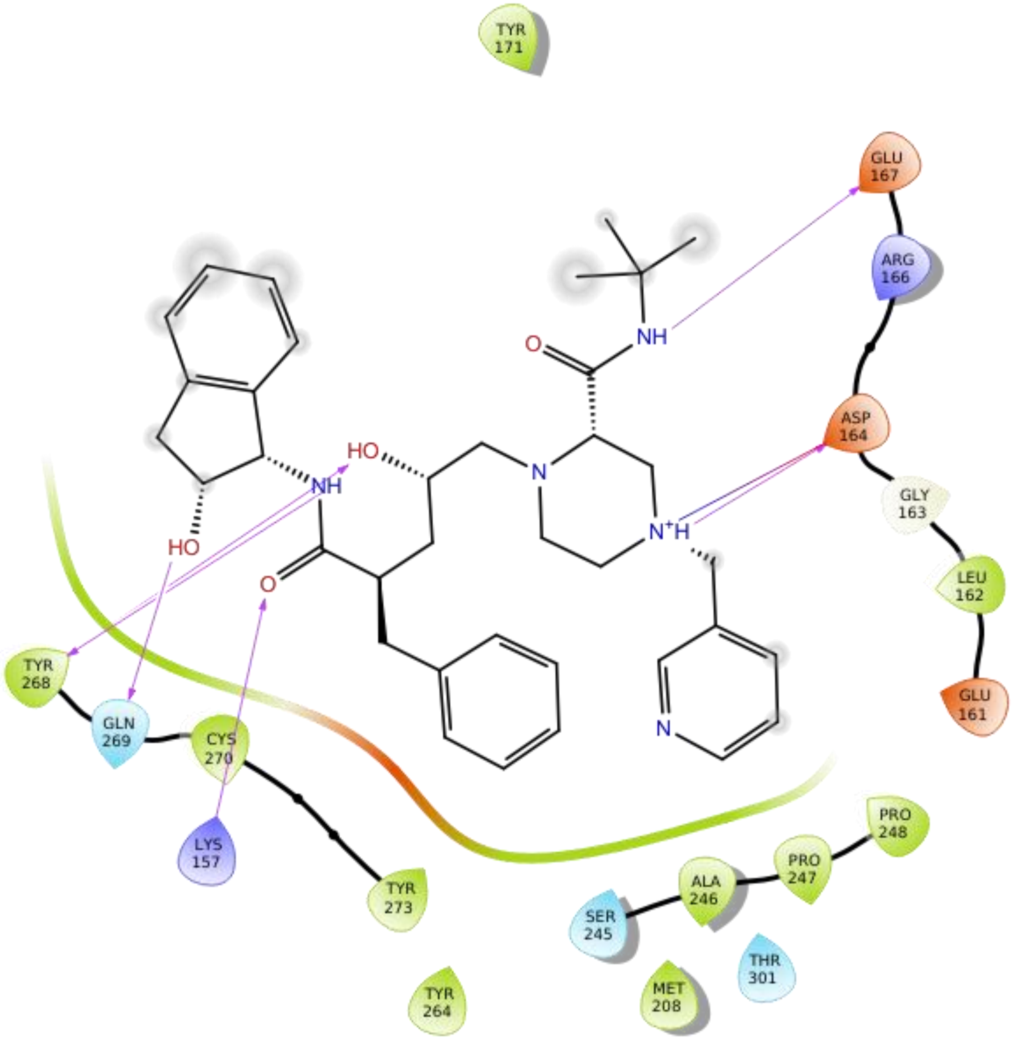

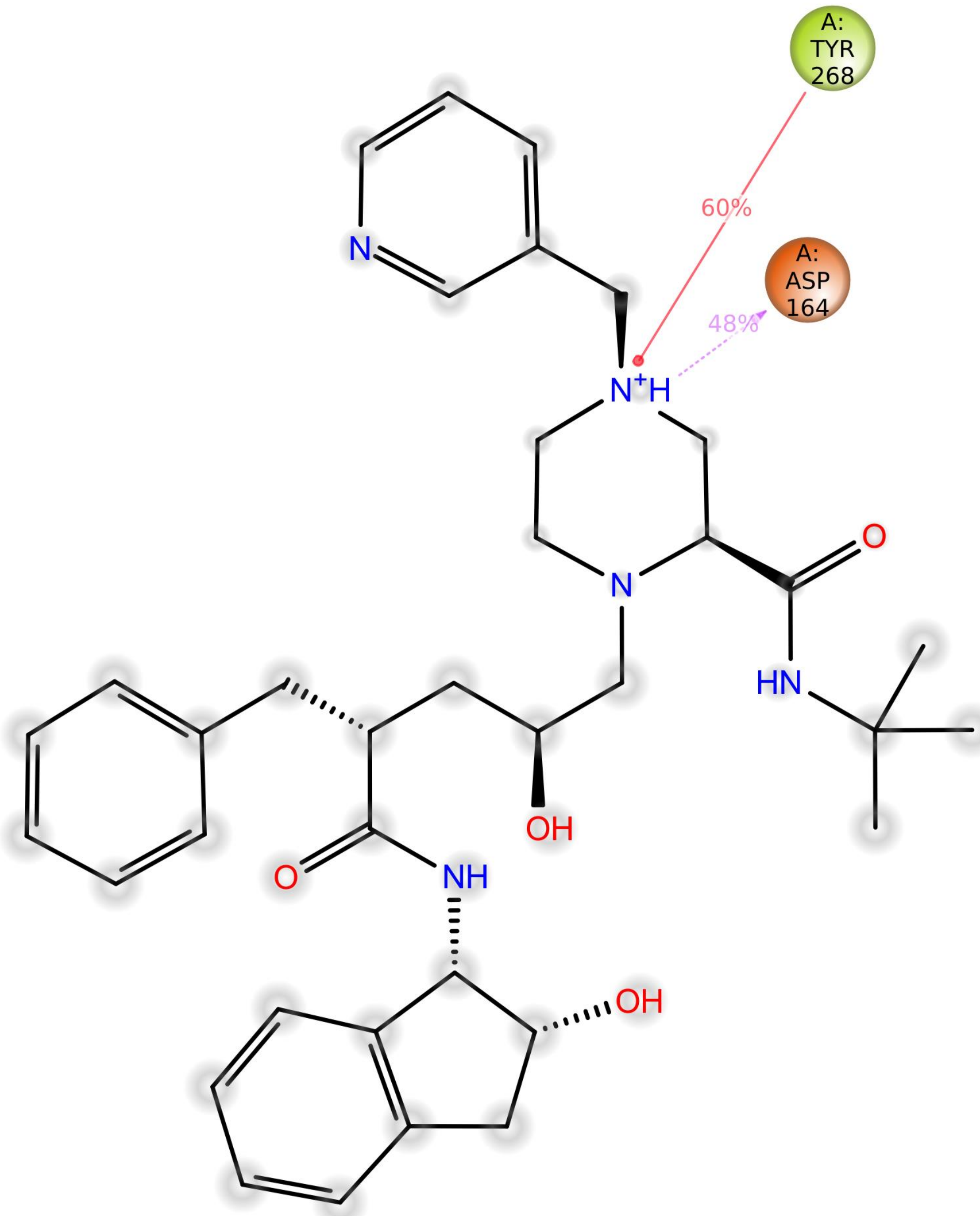
A:
TYR
268
60%
A:
ASP
164
48%
N
N⁺H
N
O
HN
OH
O
NH
OH

Indinavir in Y268 (State of protonation 2):

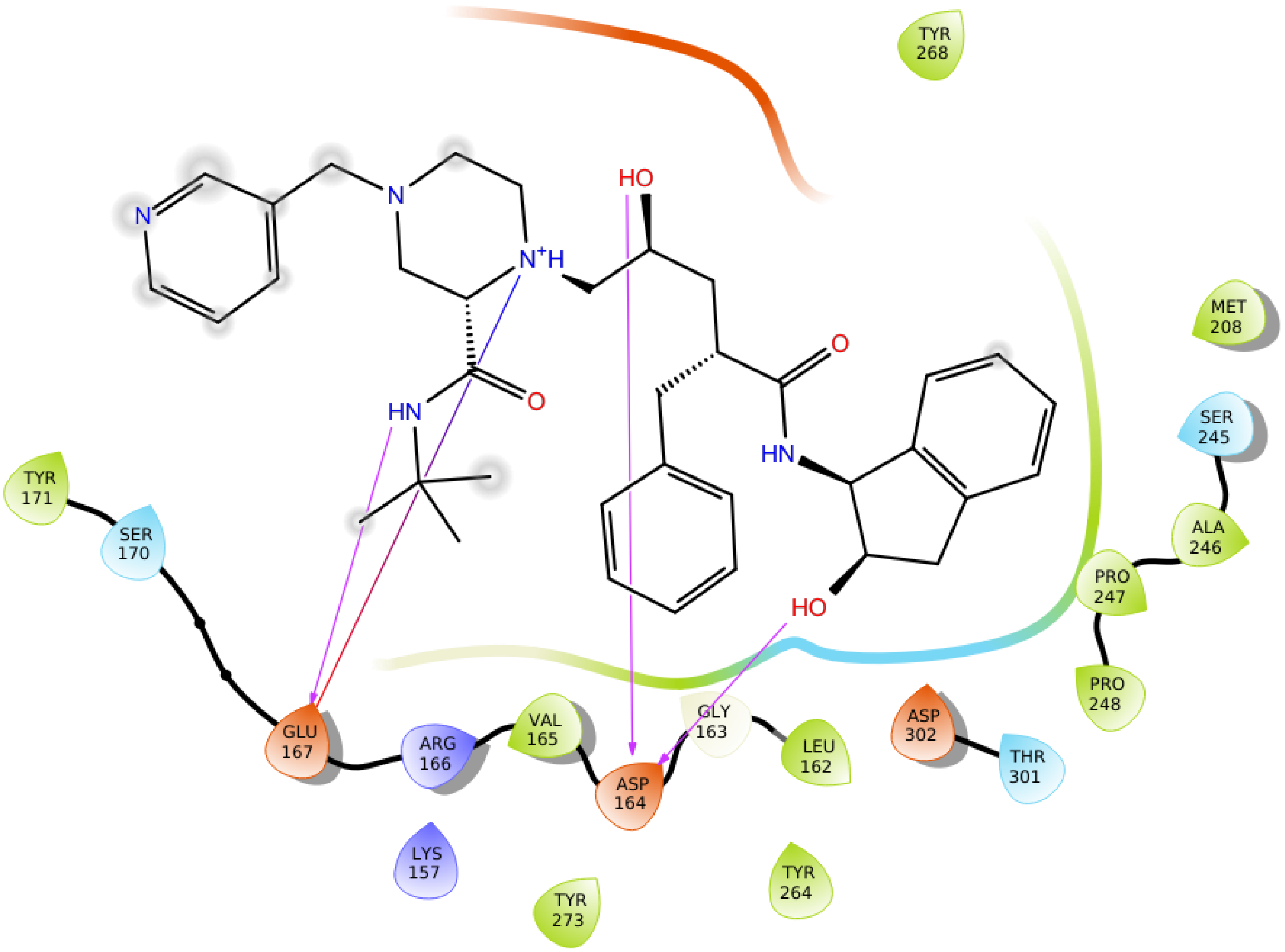

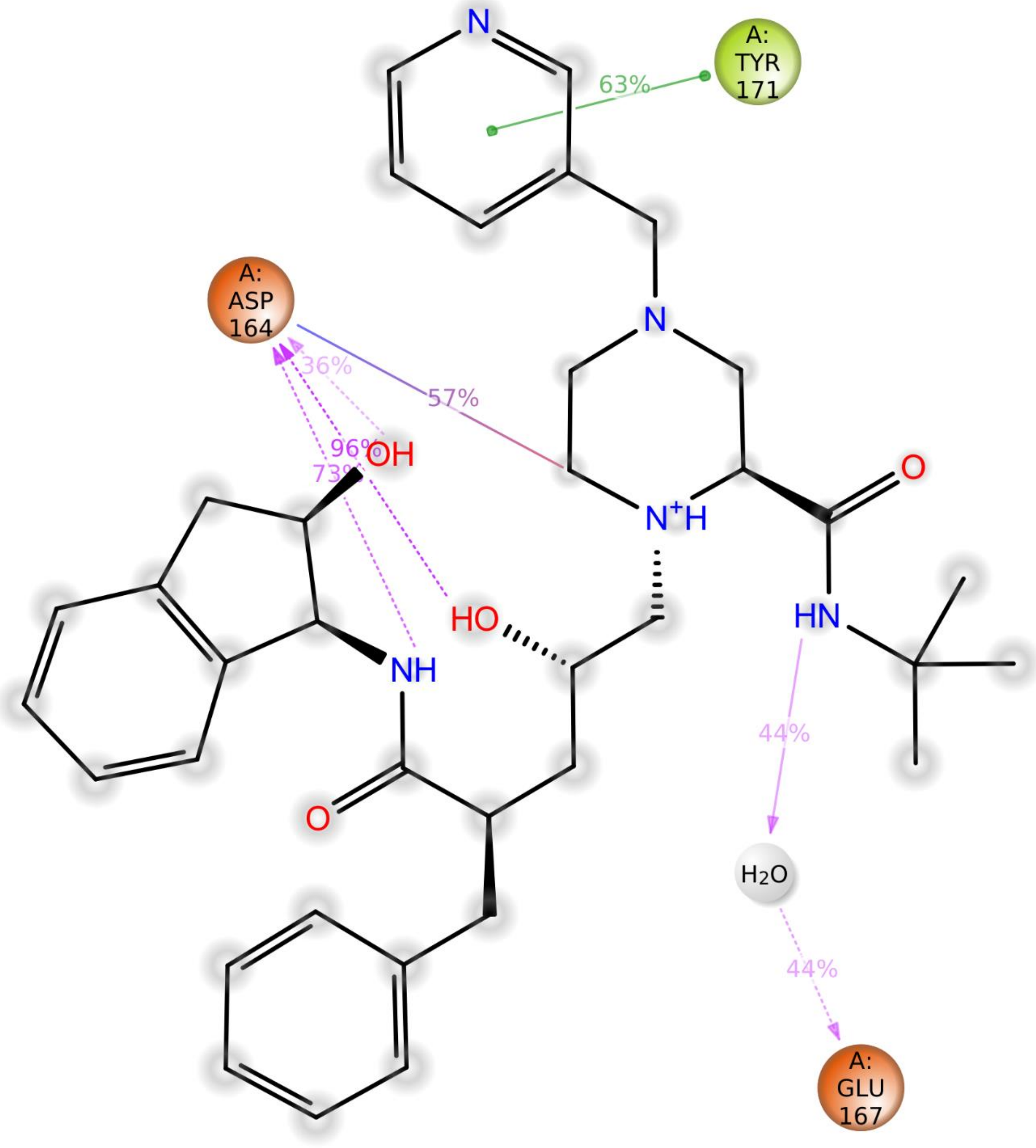

N
A:
TYR
171
63%
A:
ASP
164
36%
57%
96%
73%
OH
N
N⁺H
O
HN
HO
NH
O
44%
H2O
44%
A:
GLU
167

Palinavir in Y268

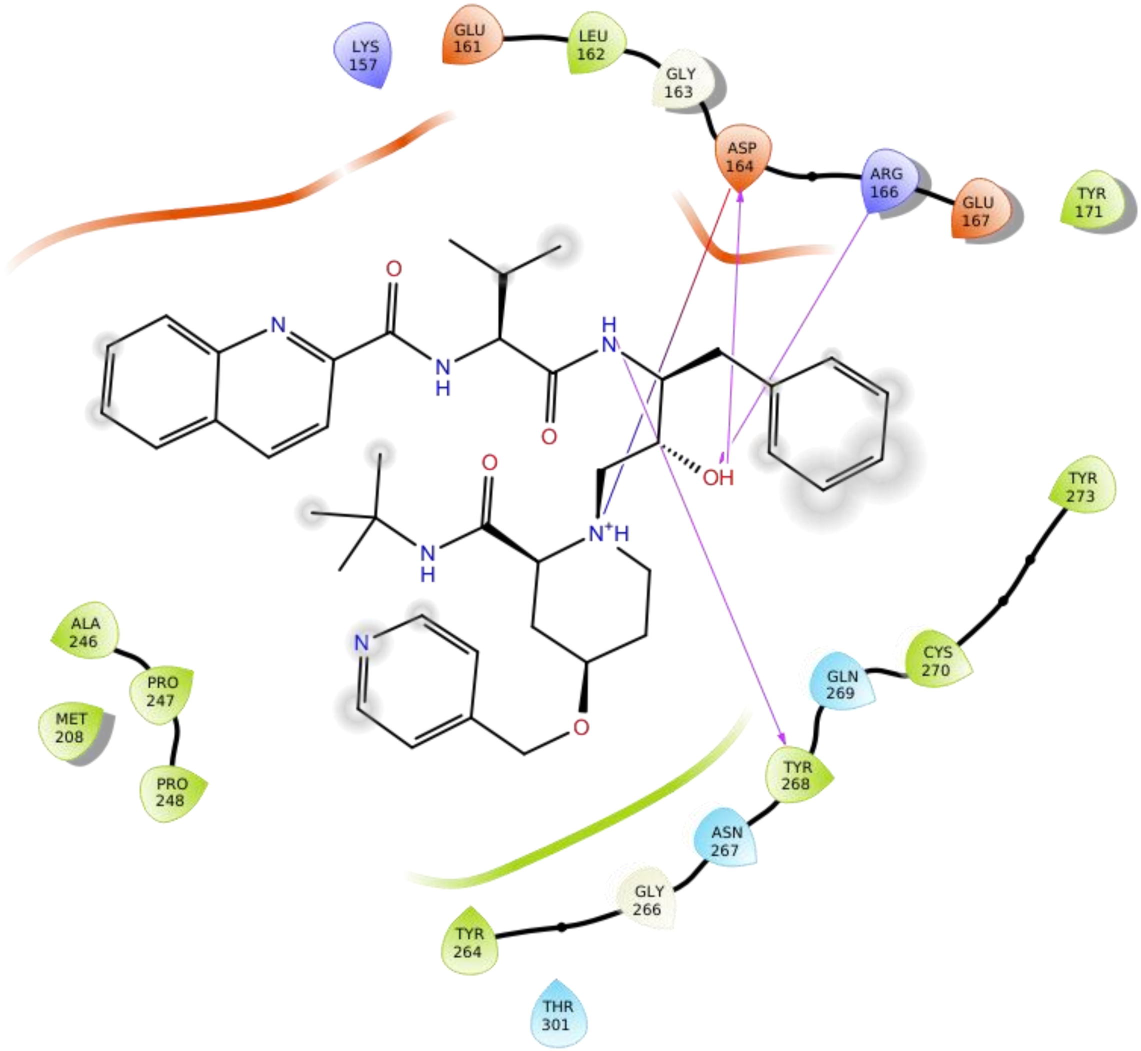

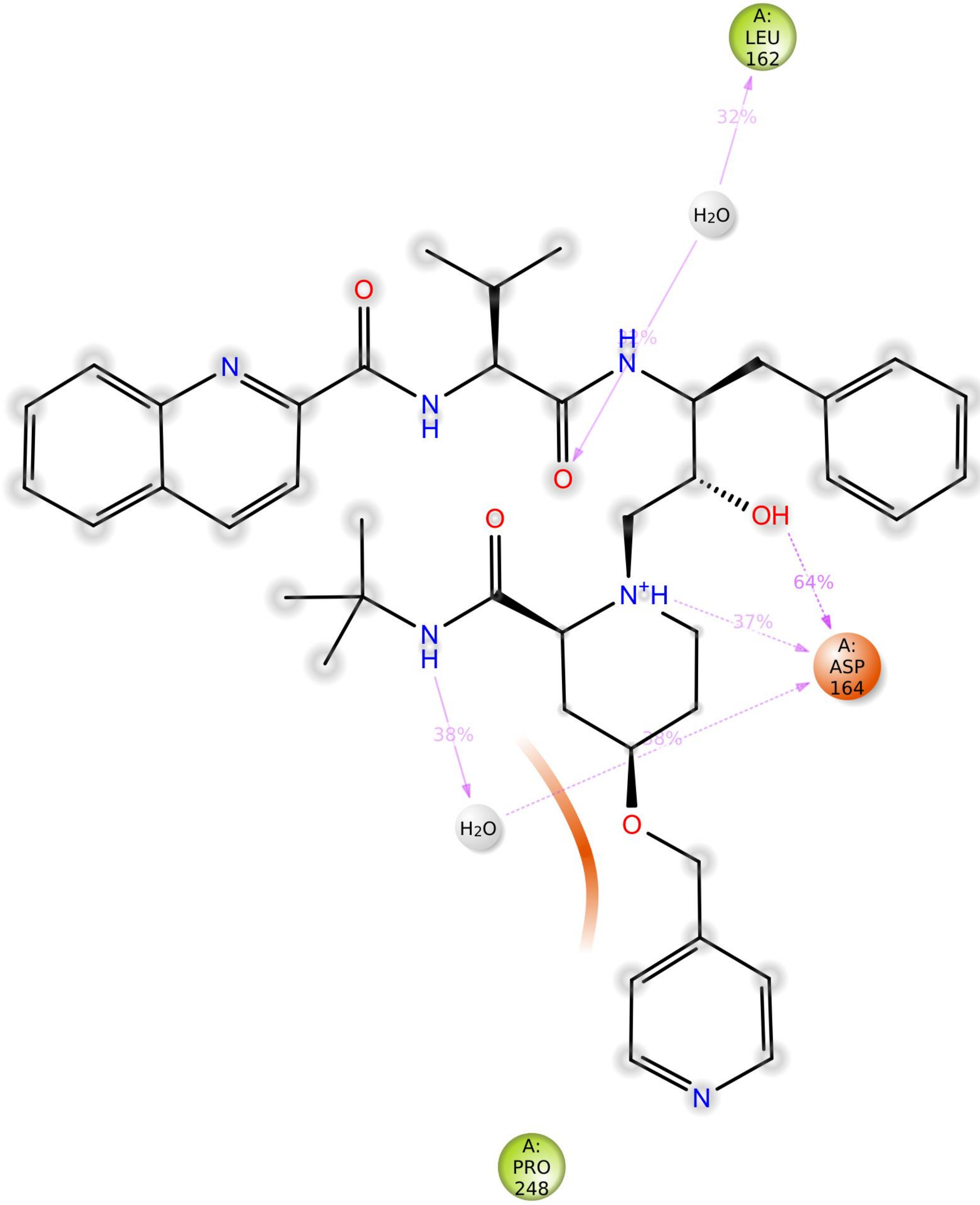
A: LEU 162
32%
H2O
64%
37%
A: ASP 164
38%
H2O
A: PRO 248

Etravirine in Y268

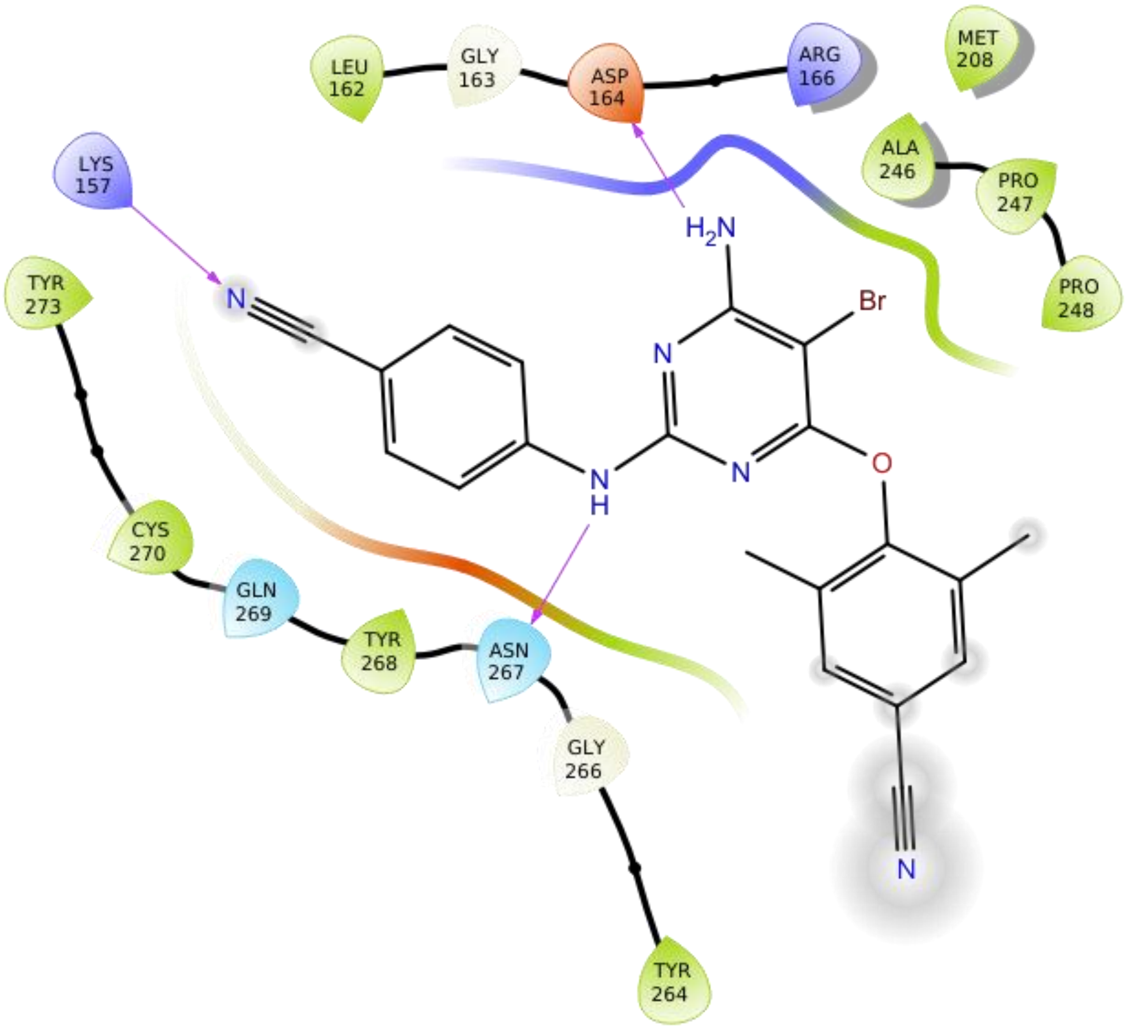

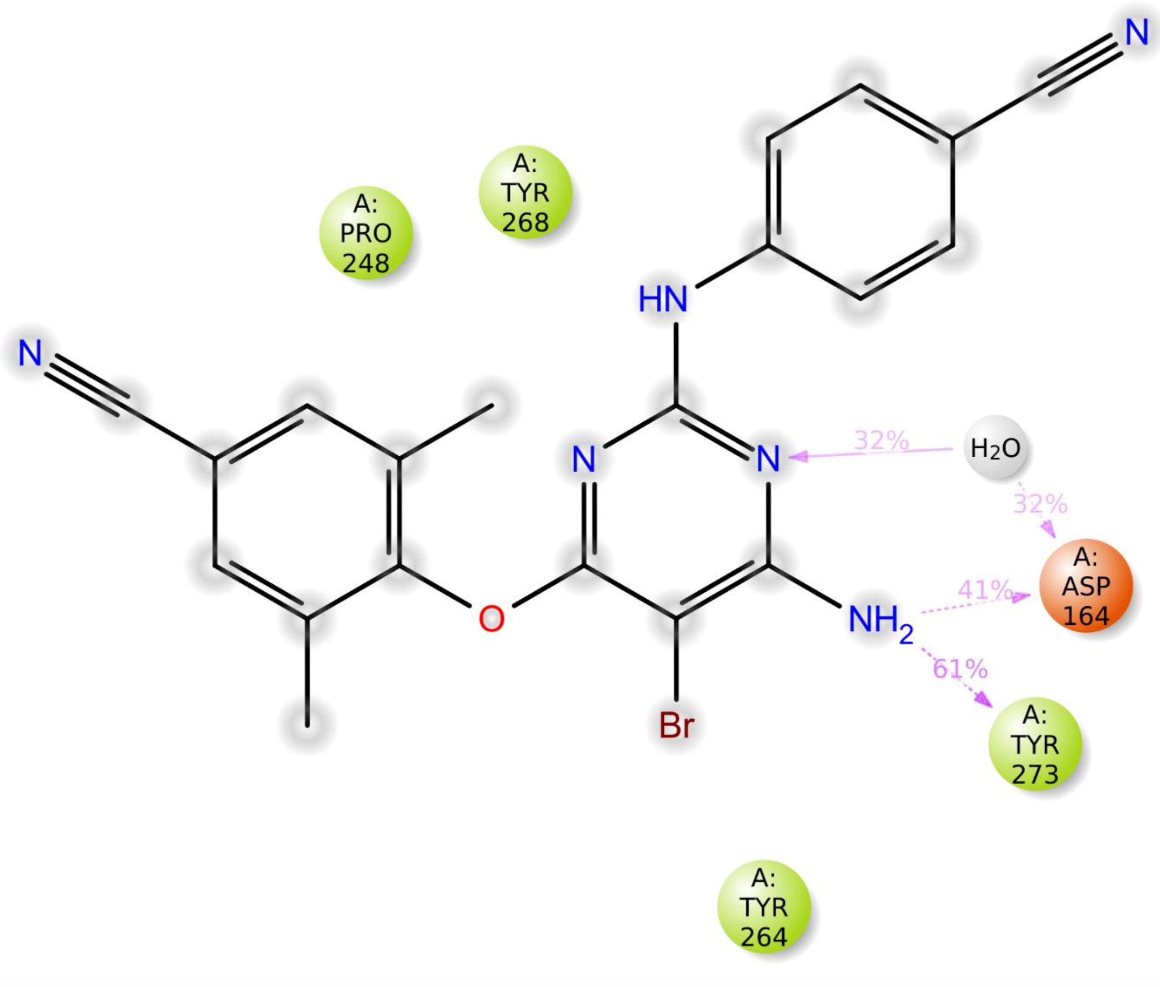

N
A:
TYR
268
A:
PRO
248
HN
N
N
N
32%
H2O
32%
A:
ASP
164
41%
O
NH2
61%
Br
A:
TYR
273
A:
TYR
264

Compound Pred12 in Y268

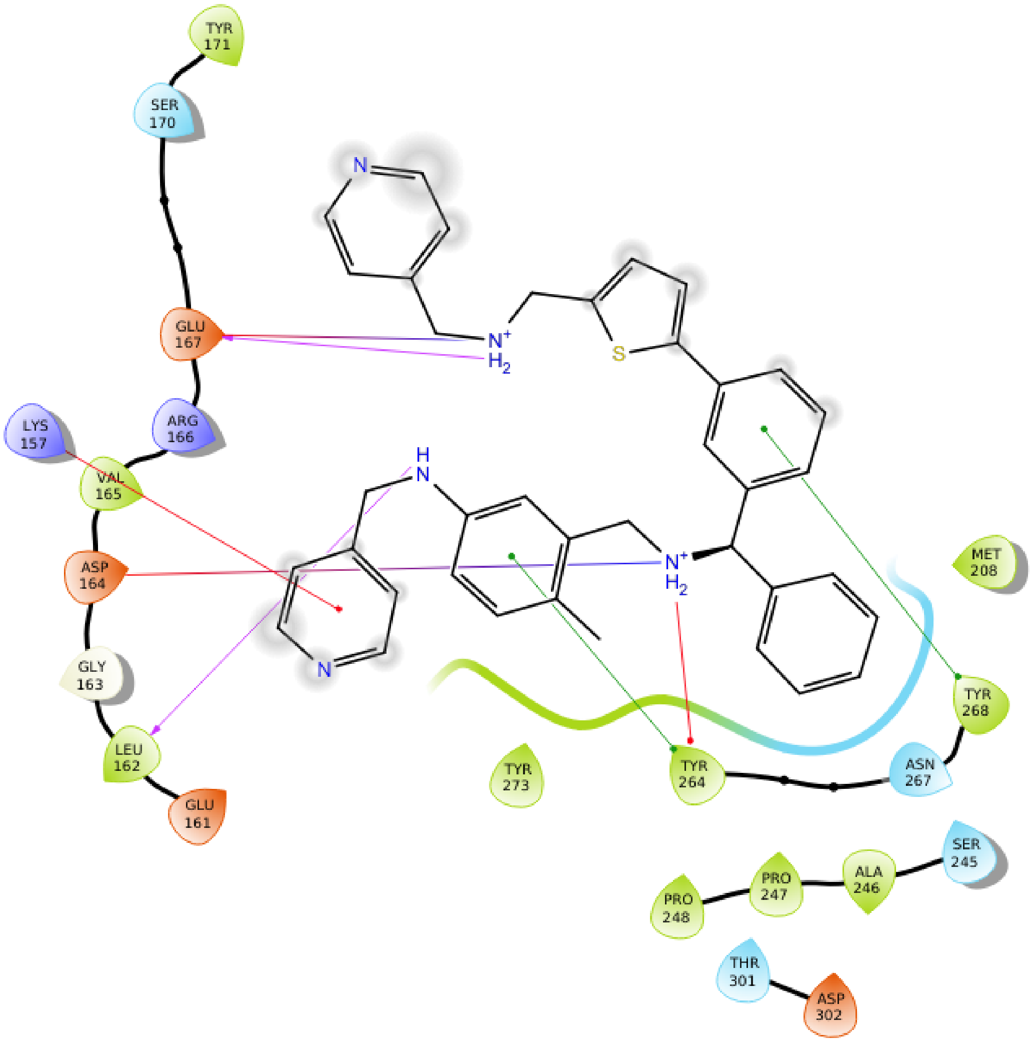

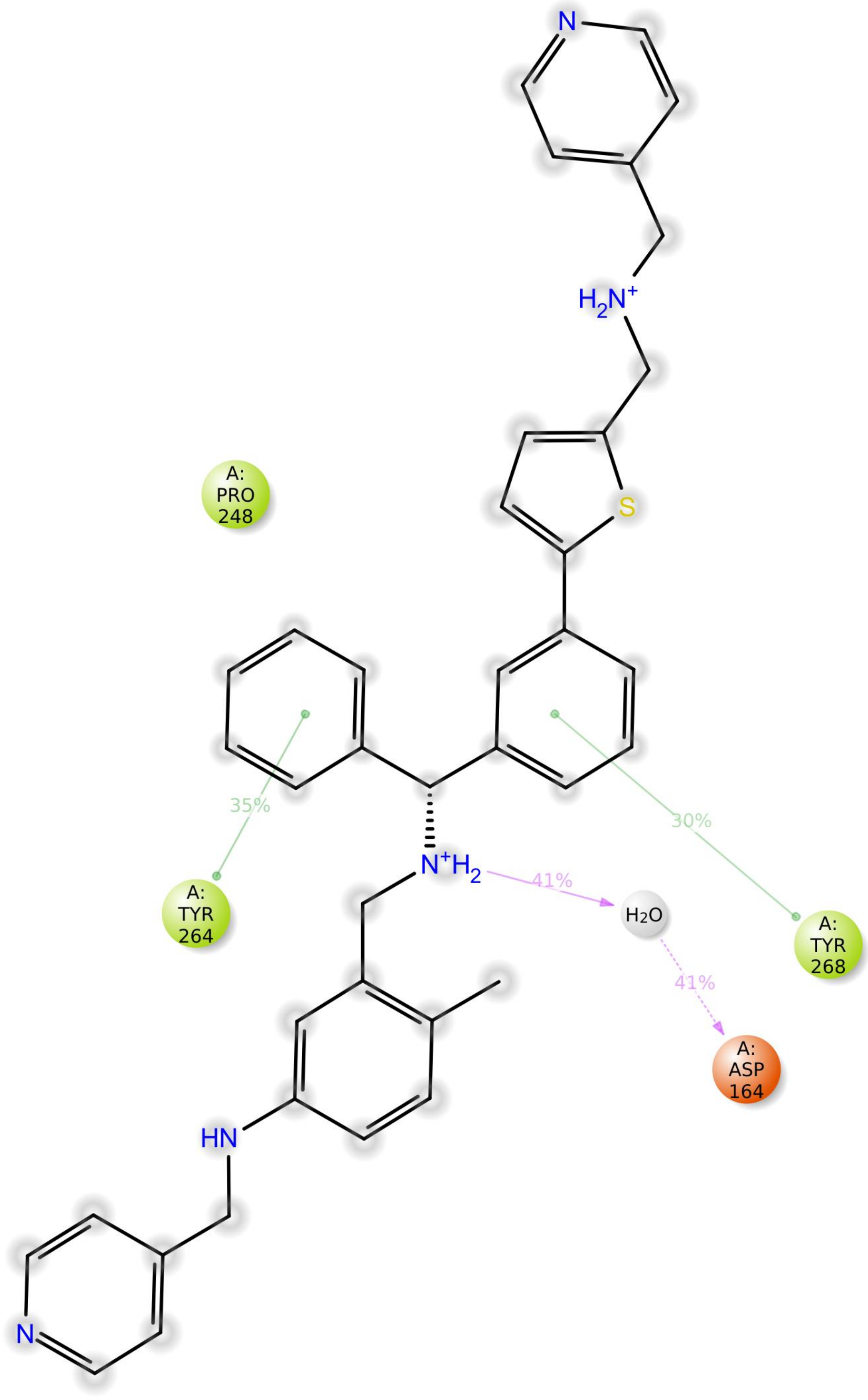
N
H2N+
S
A: PRO 248
35%
30%
N+H2
41%
H2O
41%
A: TYR 264
A: TYR 268
A: ASP 164
HN
N

Compound Pred14 in Y268

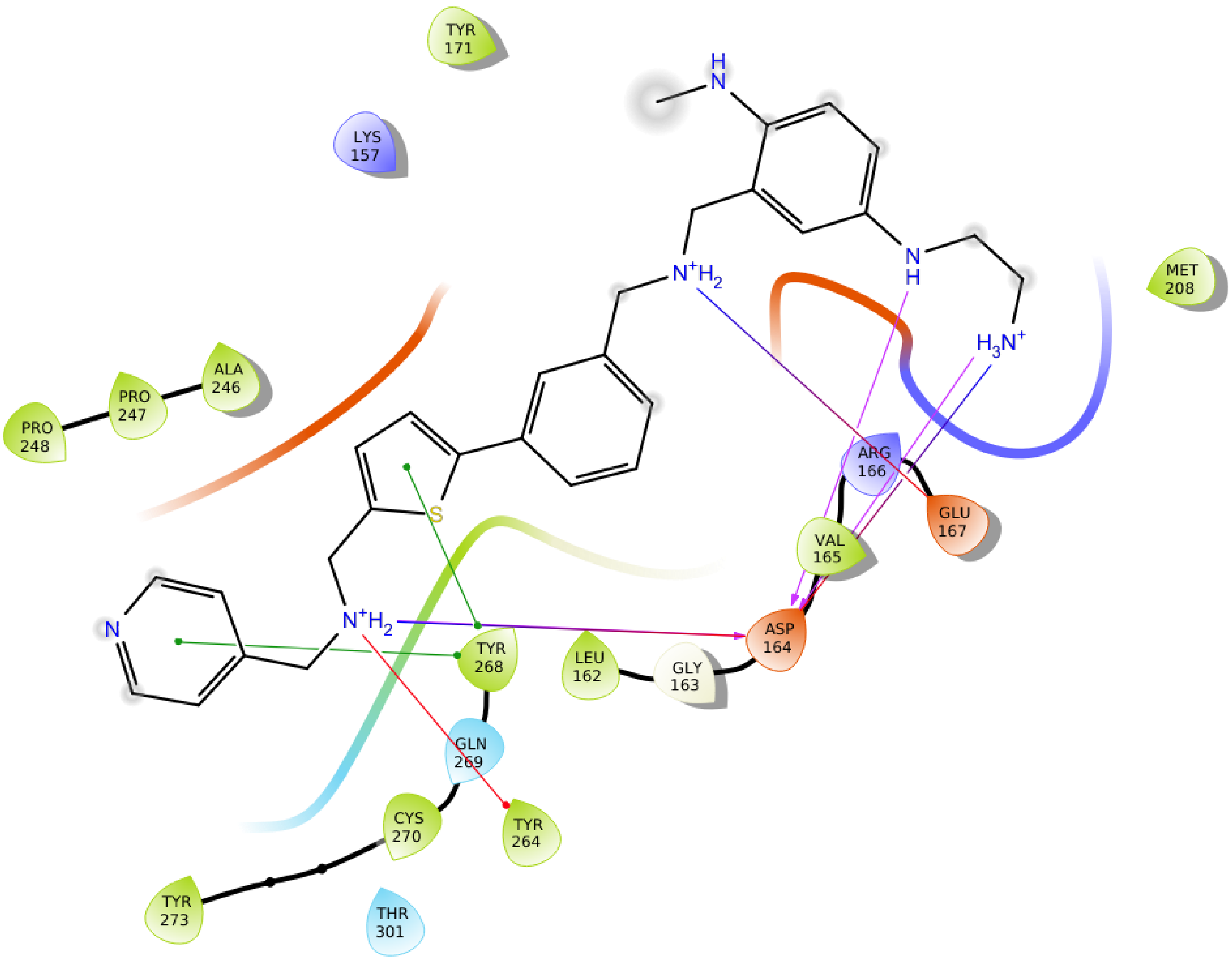

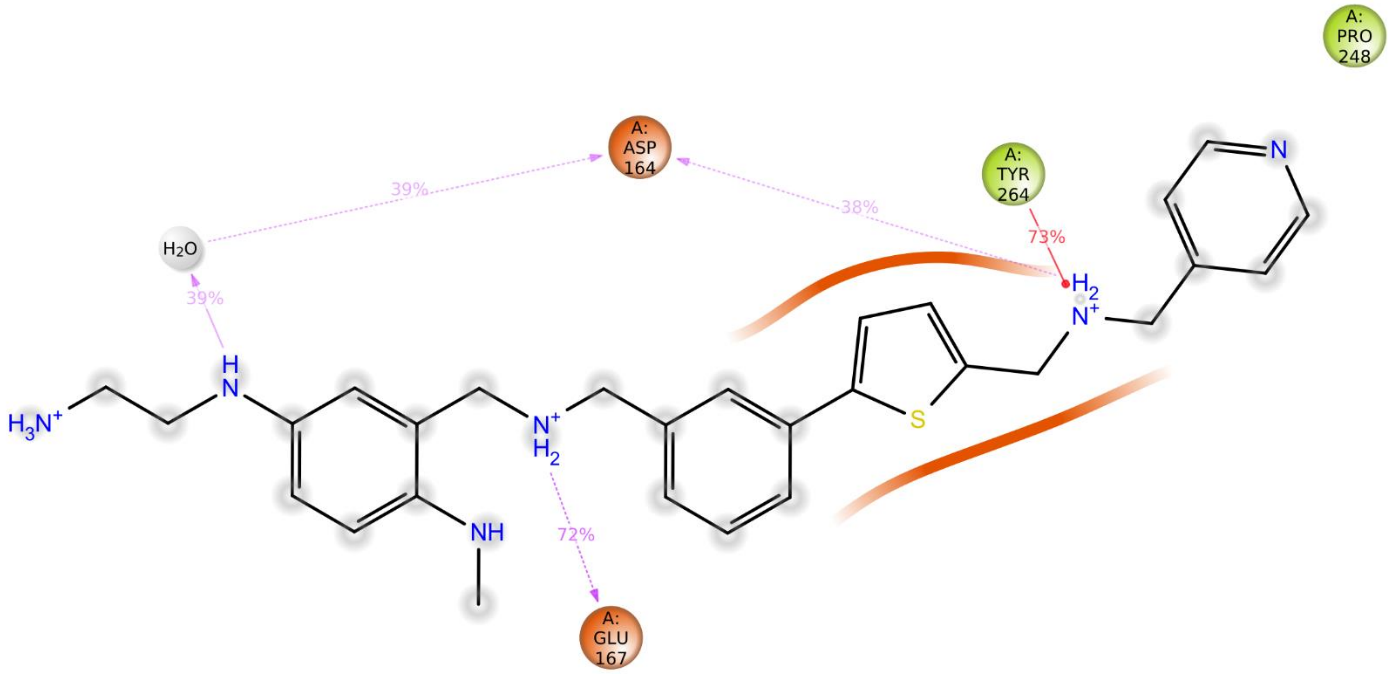
A:
PRO
248
A:
ASP
164
A:
TYR
264
39%
38%
73%
H₂O
39%
72%
A:
GLU
167

Compound Pred15 in Y268

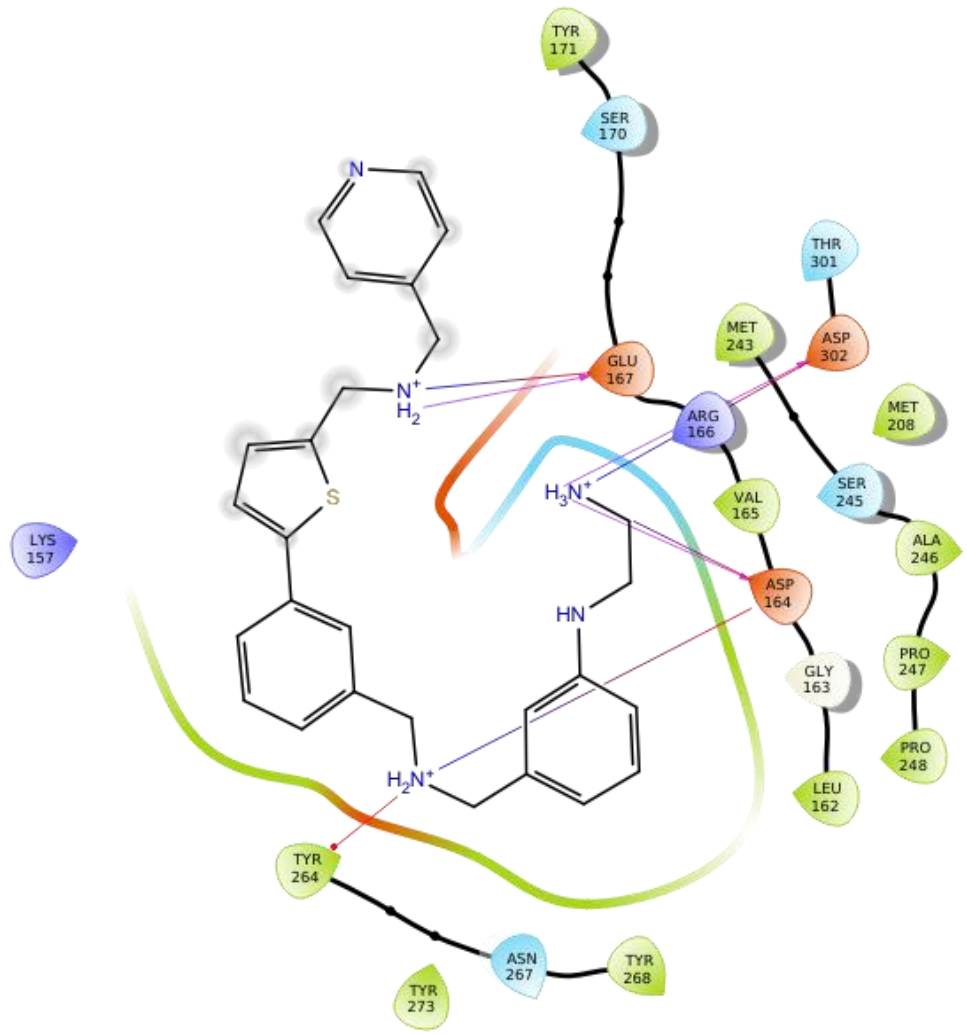

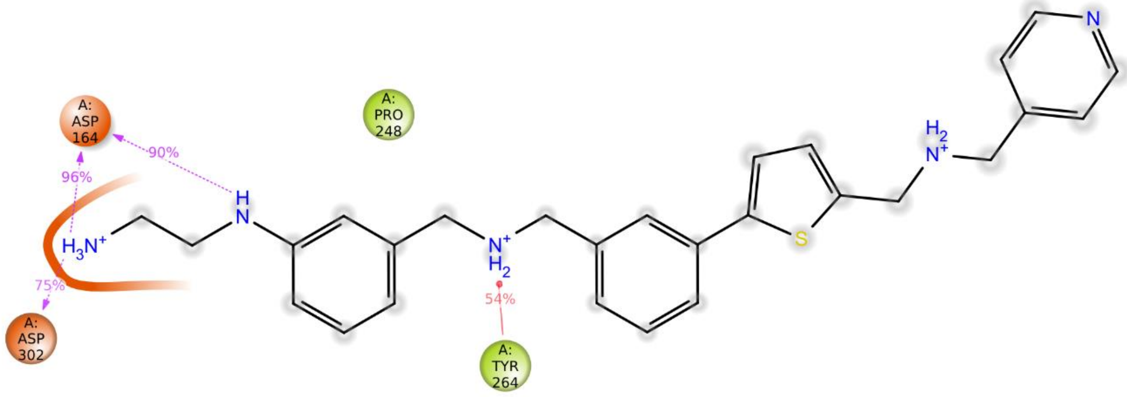

Compound Pred10 in Y268

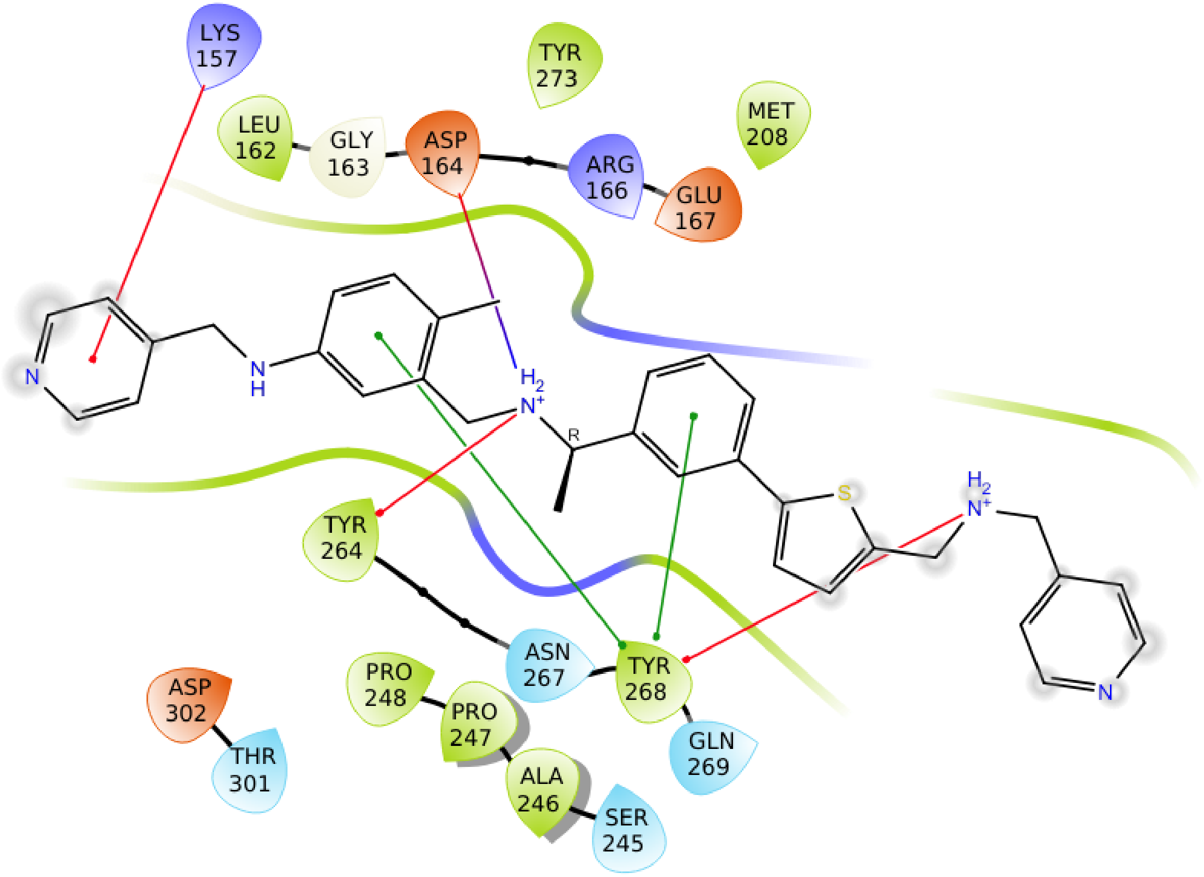

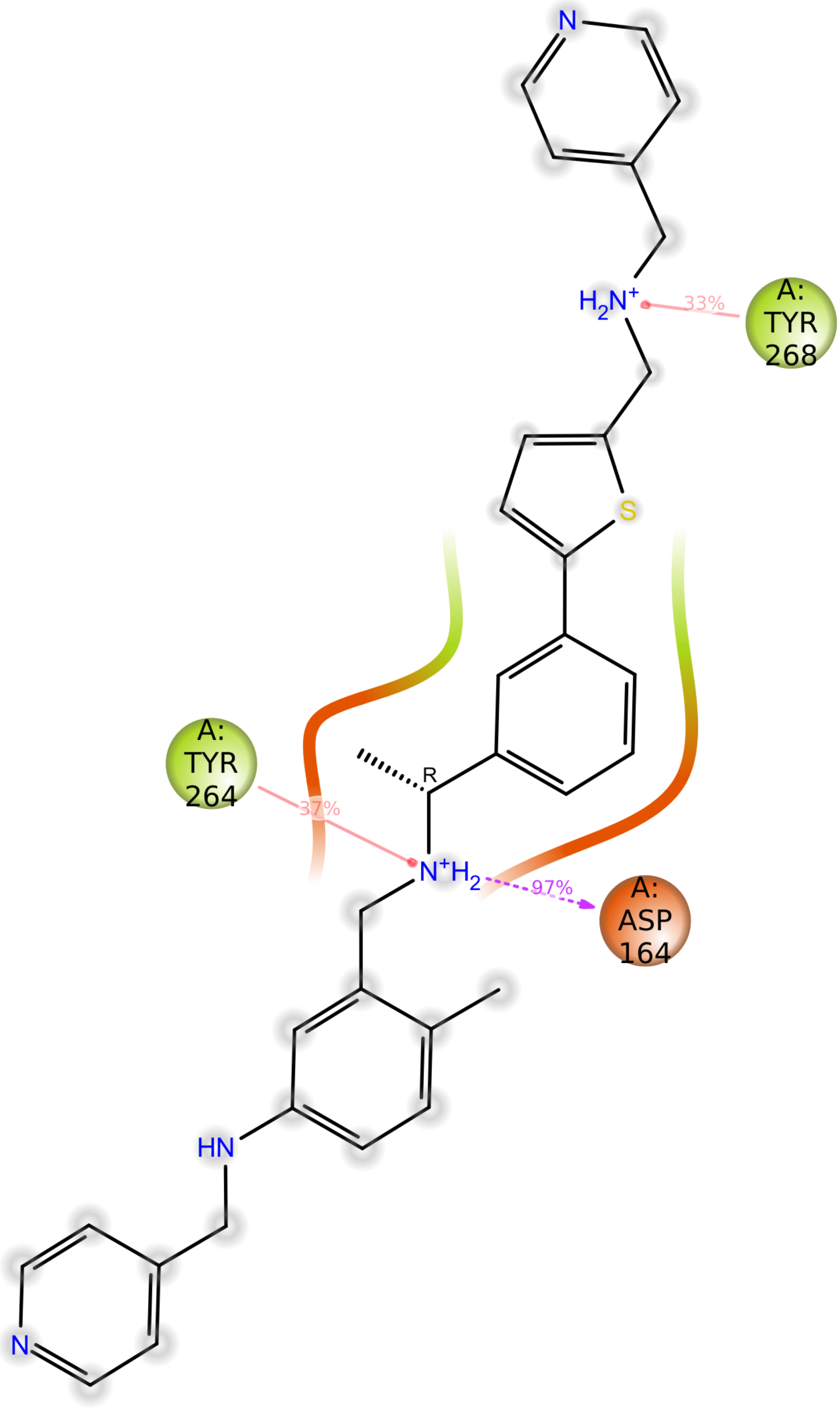
N
H2N+
33%
A: TYR 268
S
A: TYR 264
R
37%
N+H2
97%
A: ASP 164
HN
N

Etravirine in C111

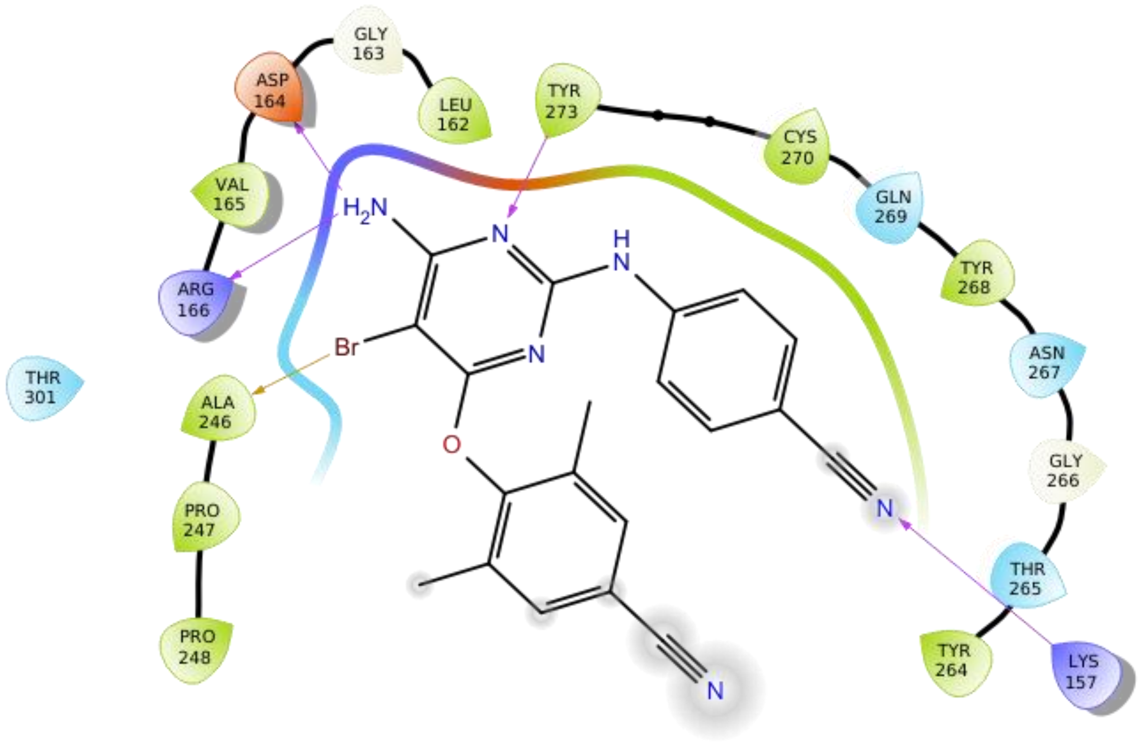

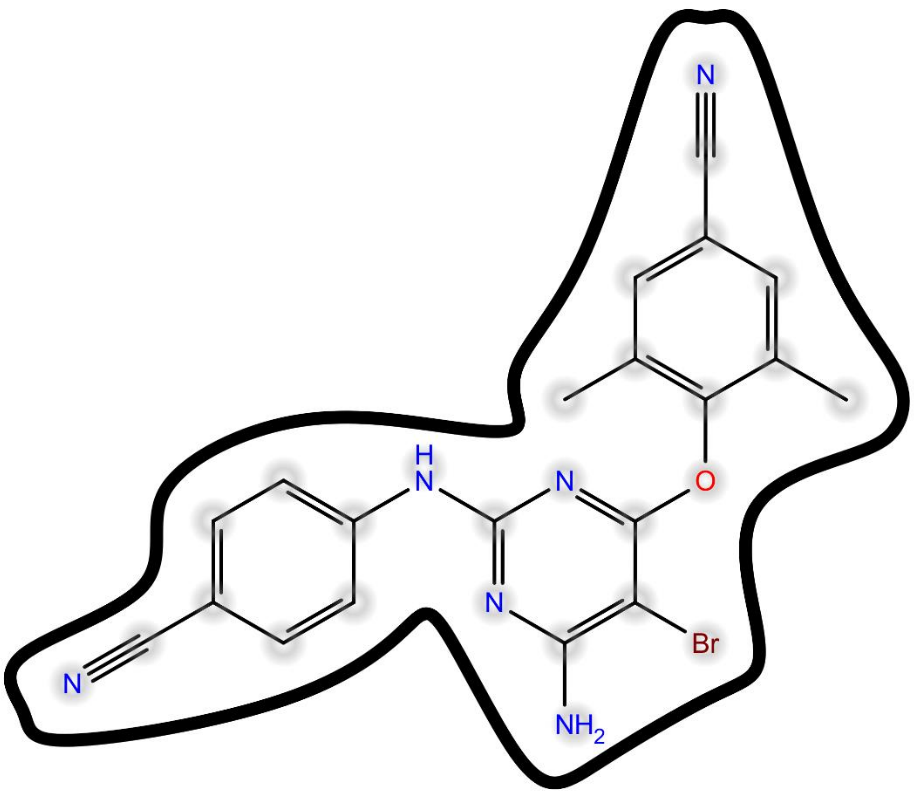
N
H
N
N
O
N
Br
NH2
N

Indinavir in C111 (State of protonation 1)

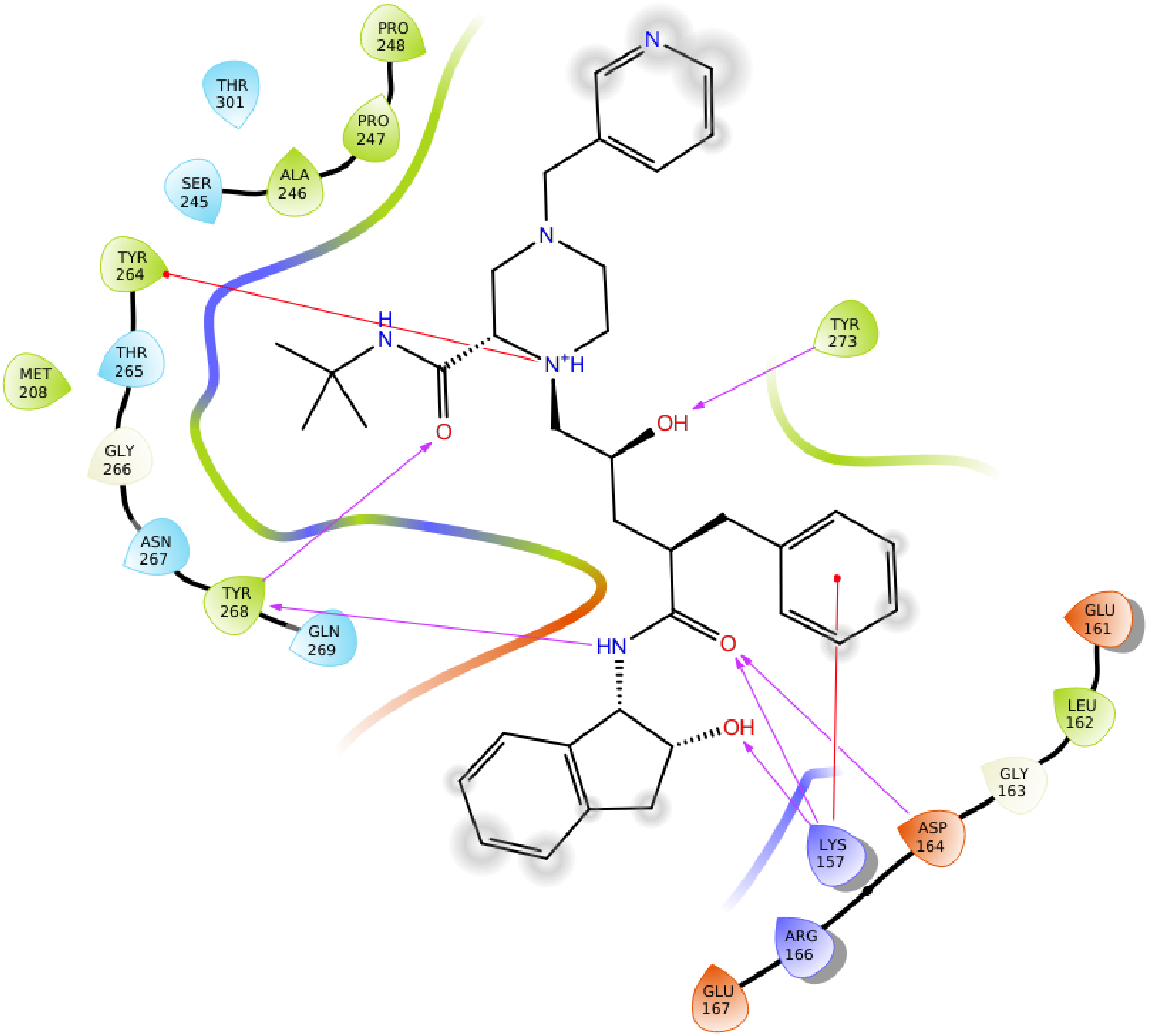

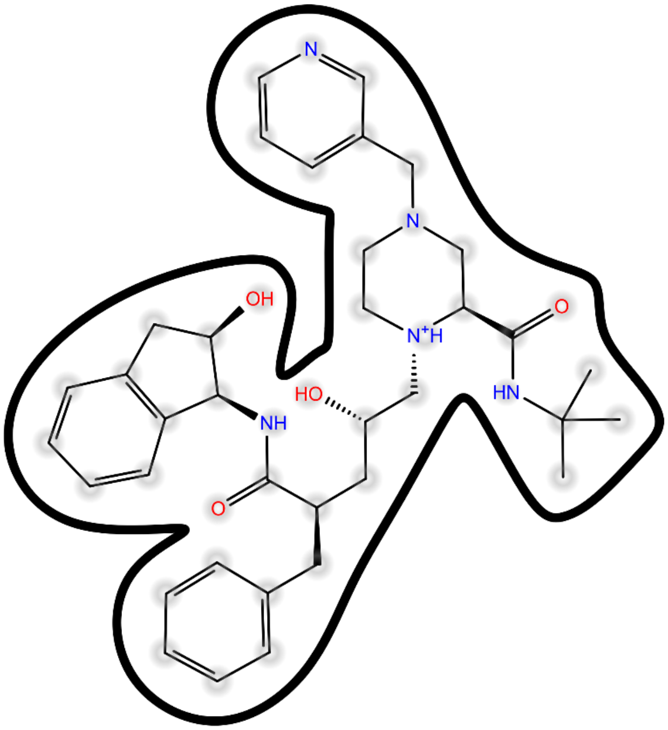
N
N
N⁺H
OH
HO
NH
O
O
HN

Indinavir in C111 (State of protonation 2)

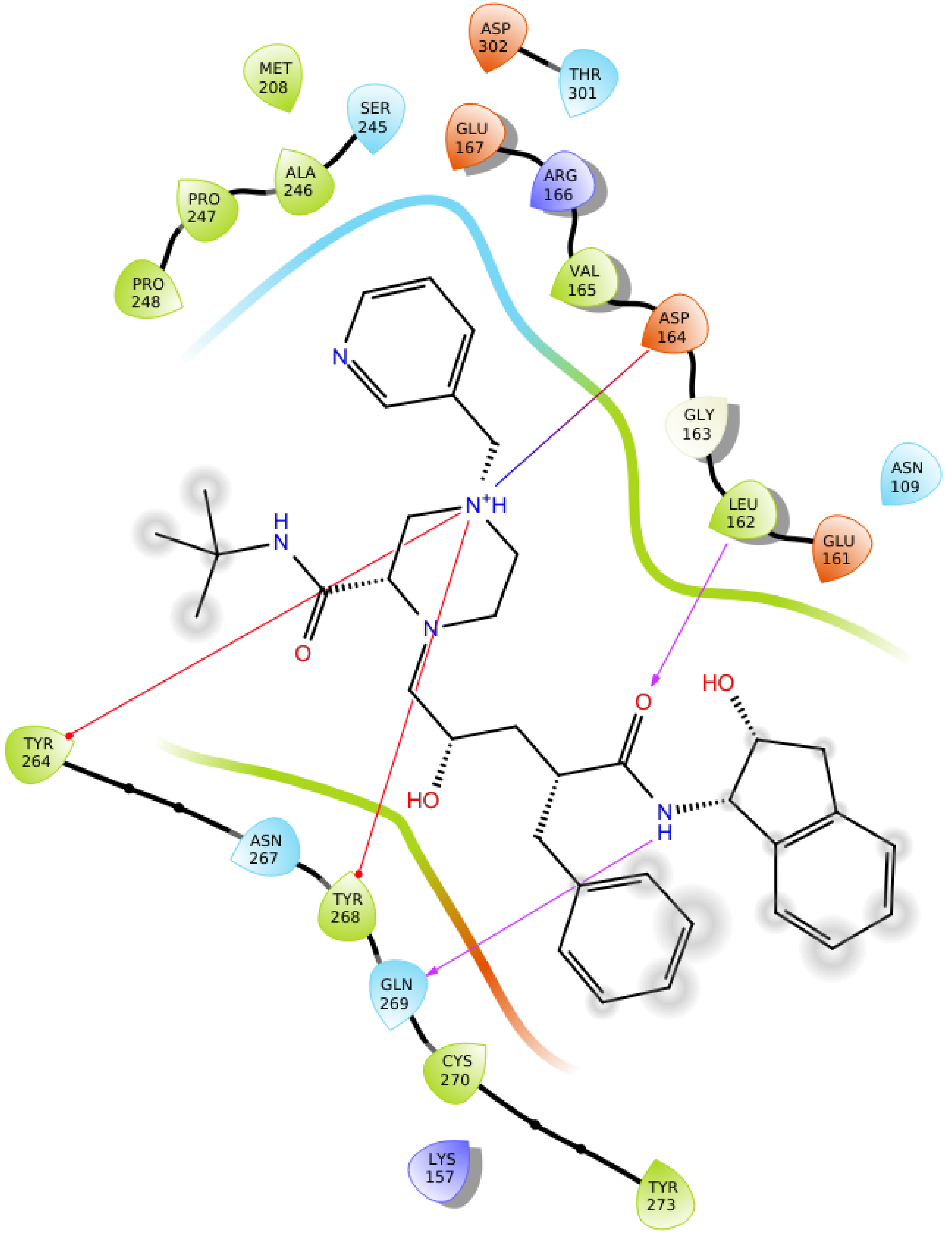

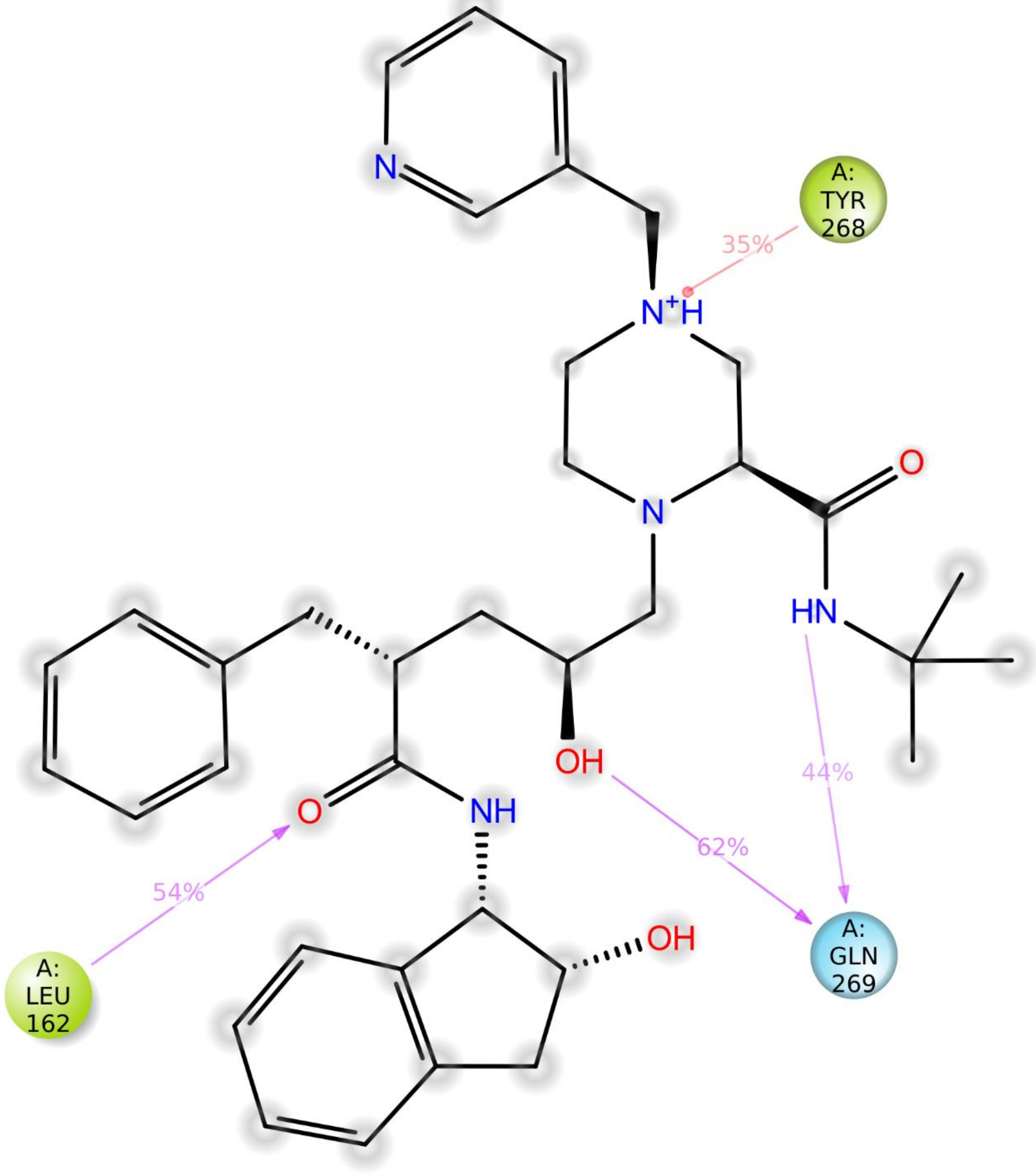

N
N+H
A: TYR 268
35%
O
N
HN
44%
OH
62%
O
NH
54%
OH
A: LEU 162
A: GLN 269

Lopinavir in C111

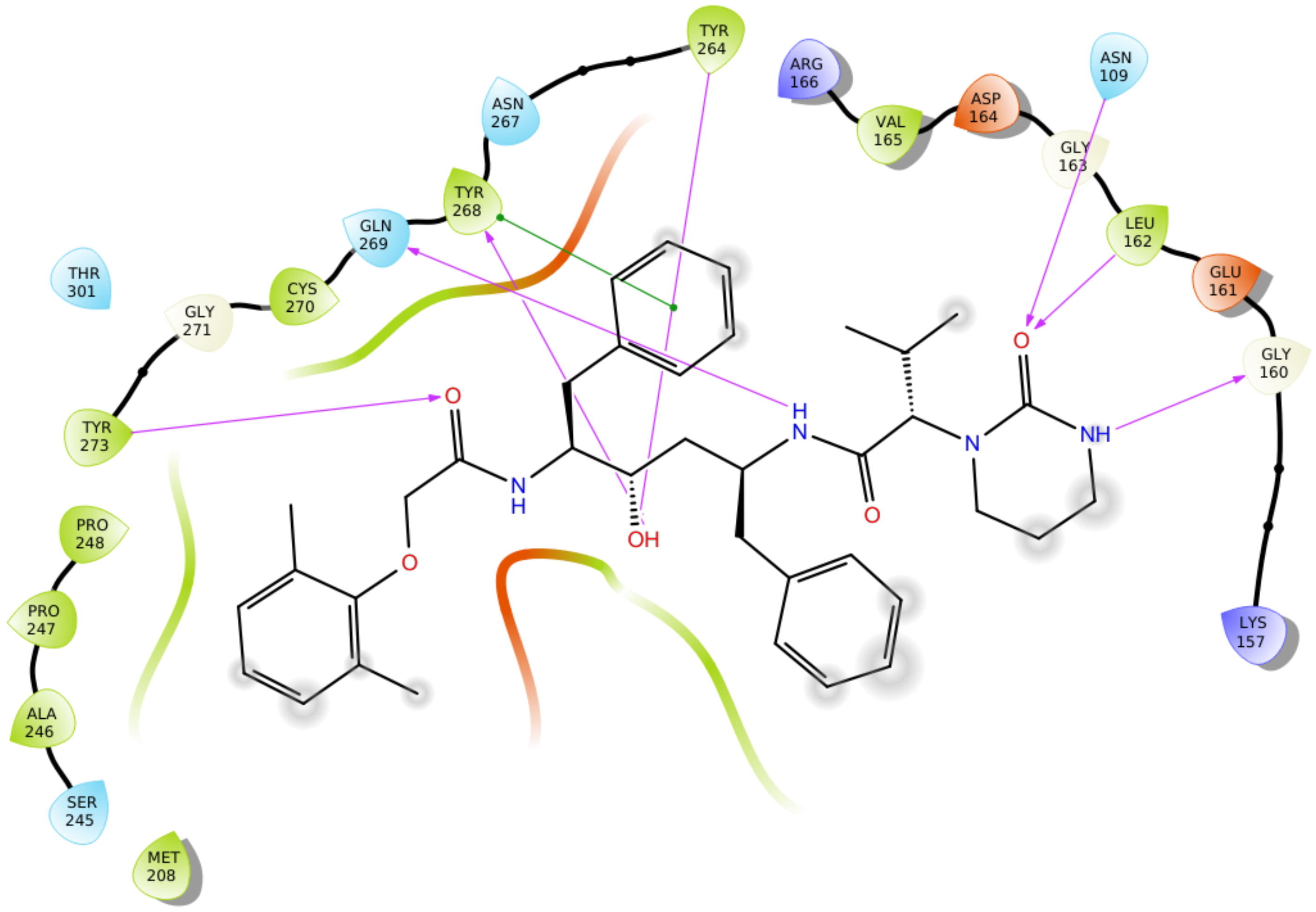

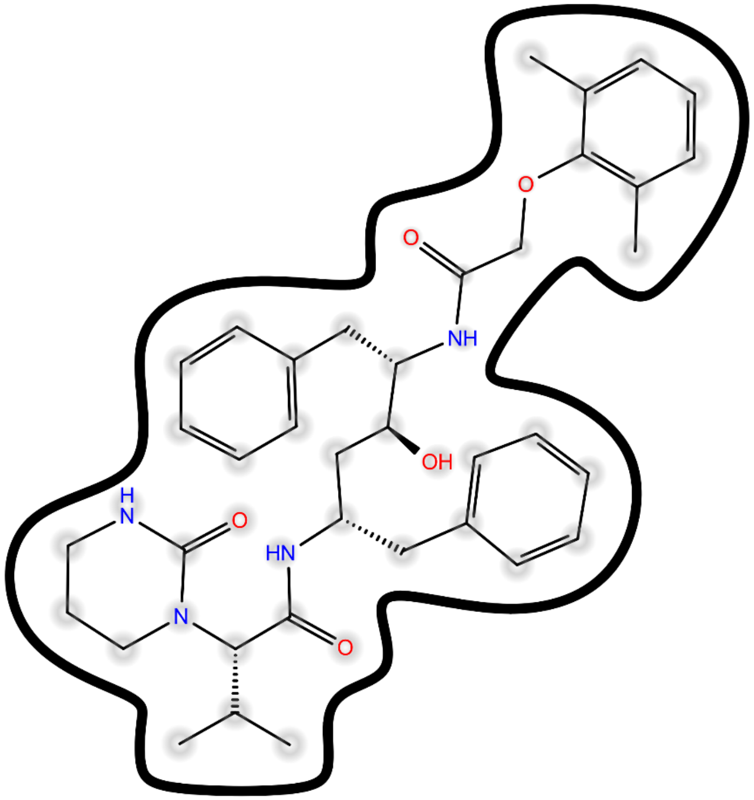
O
O
NH
OH
H
N
O
HN
N
O

Compound Pred6 in C111

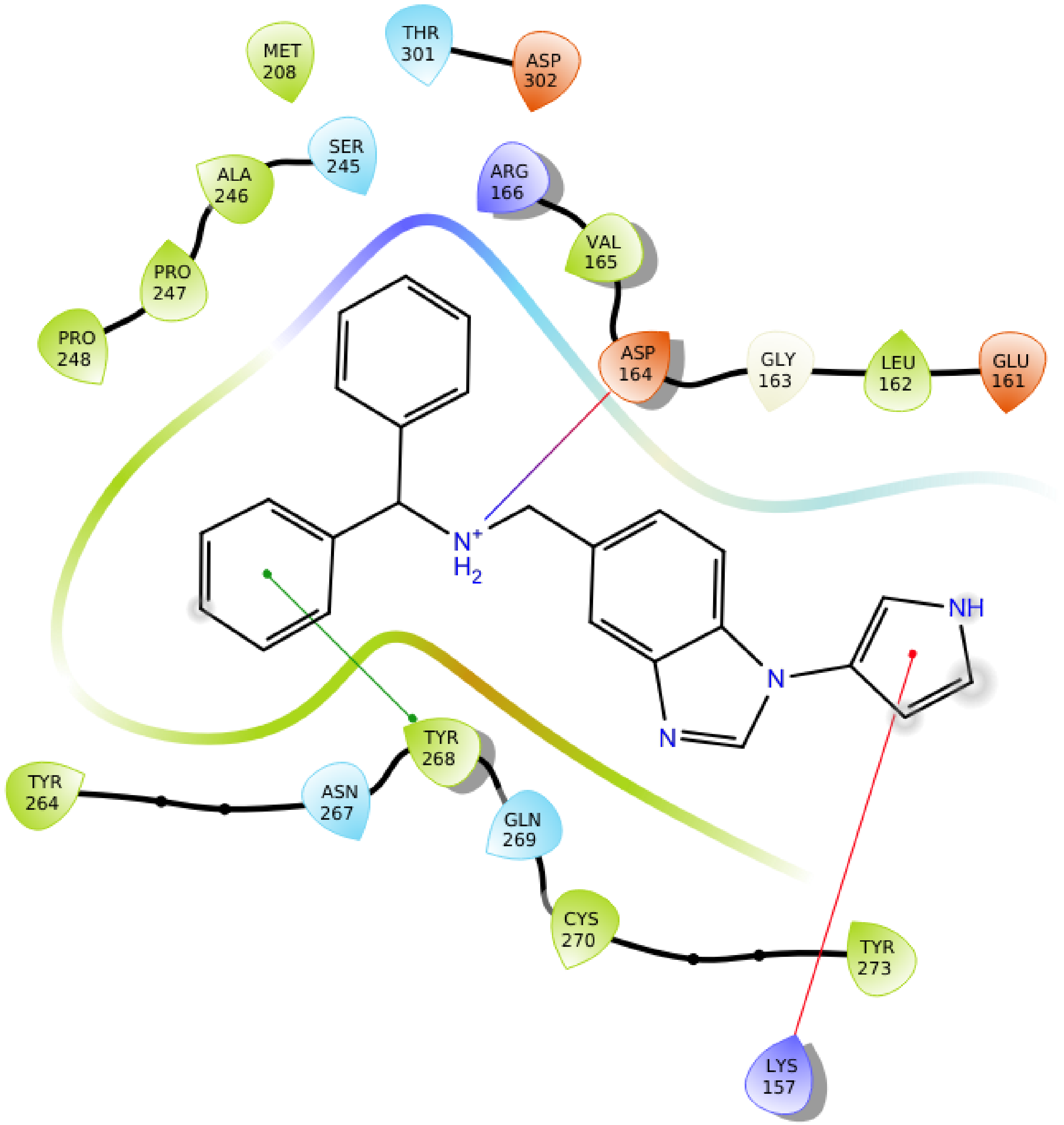

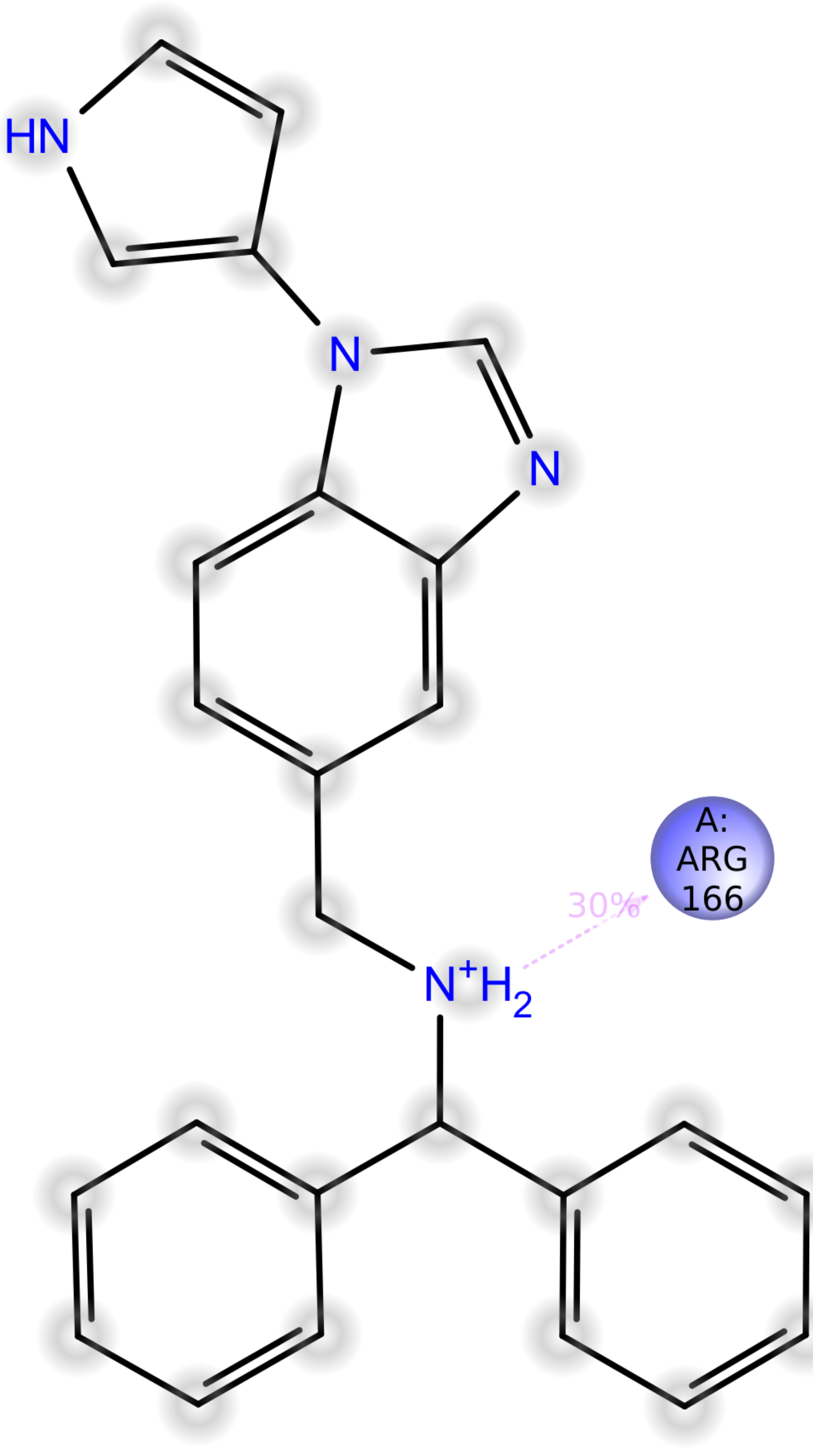
HN
N
N
N+H2
A:
ARG
166
30%

Compound Pred13 in C111

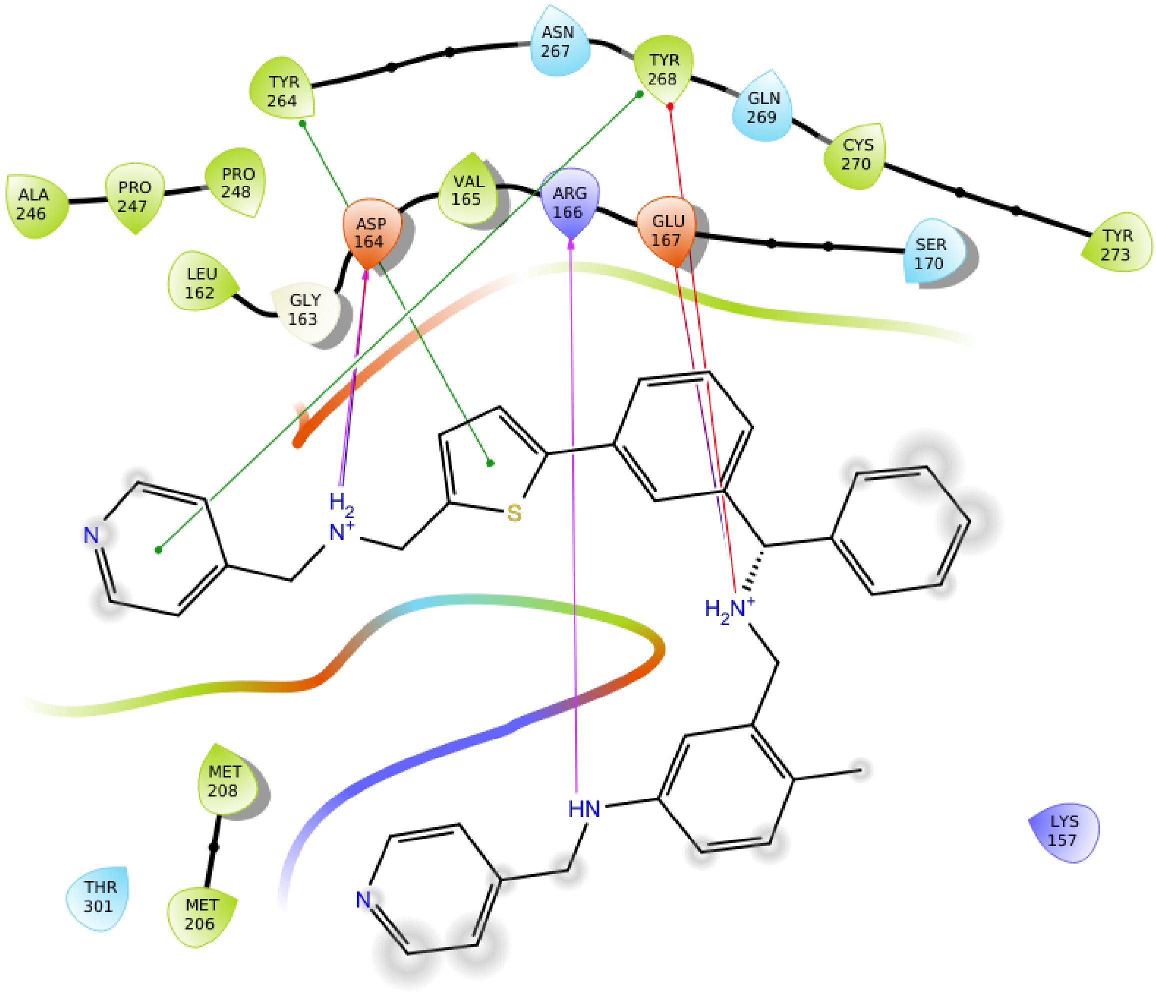

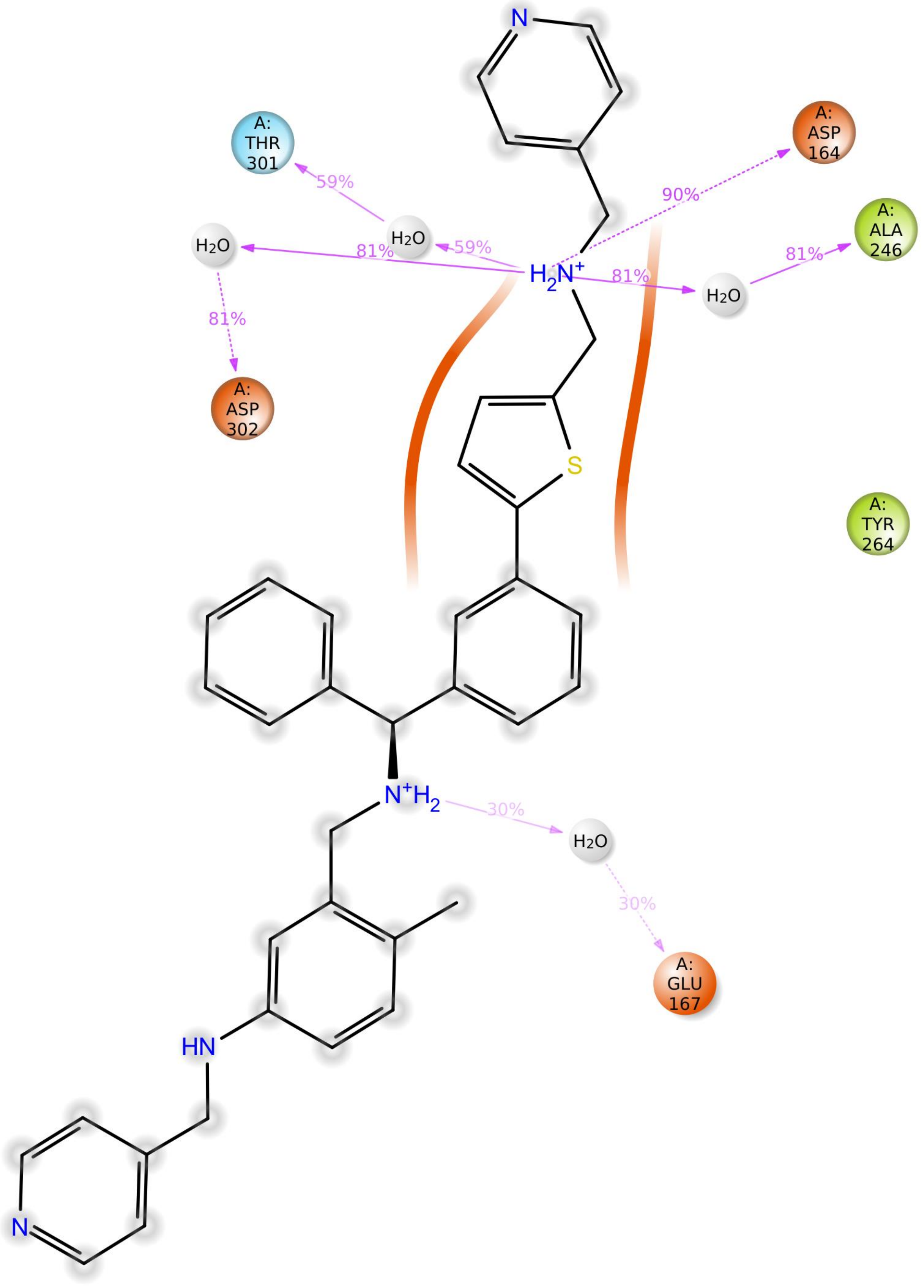
A: THR 301
A: ASP 164
A: ALA 246
A: ASP 302
A: TYR 264
A: GLU 167
H2O
H2O
H2O
H2O
59%
59%
81%
81%
81%
81%
90%
30%
30%
N
S
HN
N

Compound Pred14 in C111

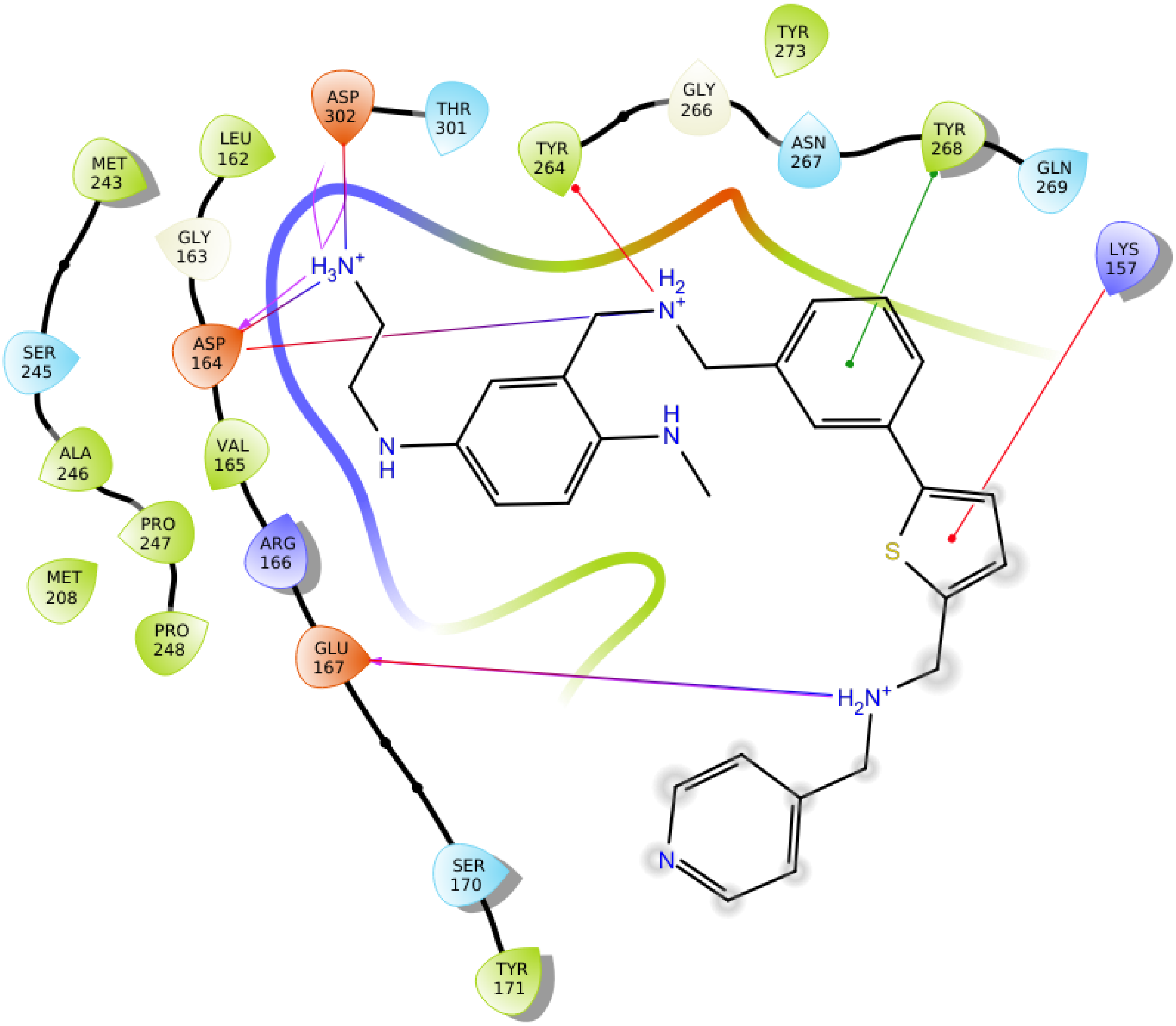

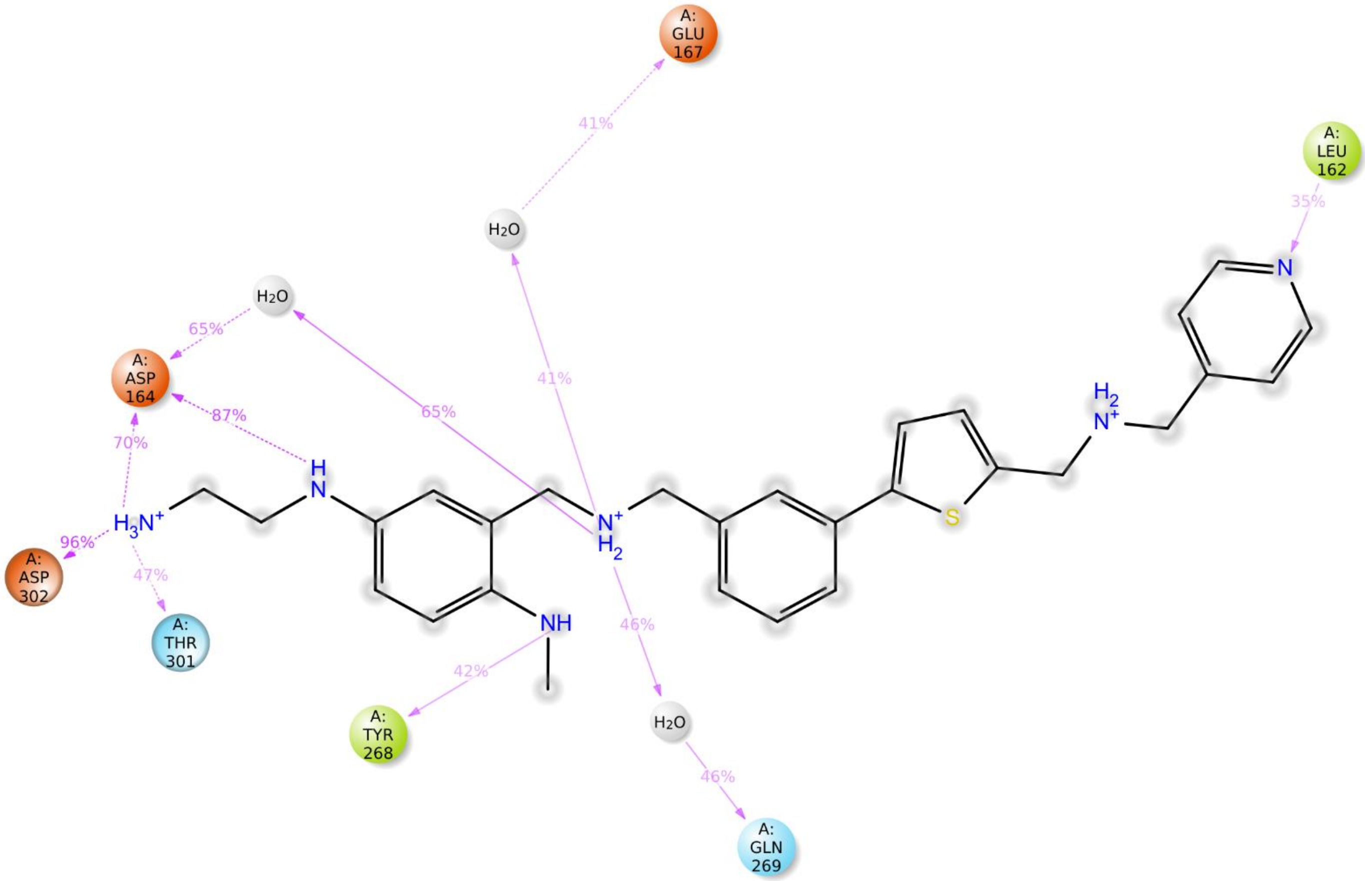
A:
GLU
167
41%
A:
LEU
162
35%
H2O
H2O
65%
A:
ASP
164
41%
87%
65%
70%
H3N+
96%
A:
ASP
302
47%
A:
THR
301
NH
42%
46%
A:
TYR
268
H2O
46%
A:
GLN
269
S
N

Compound Pred15 in C111

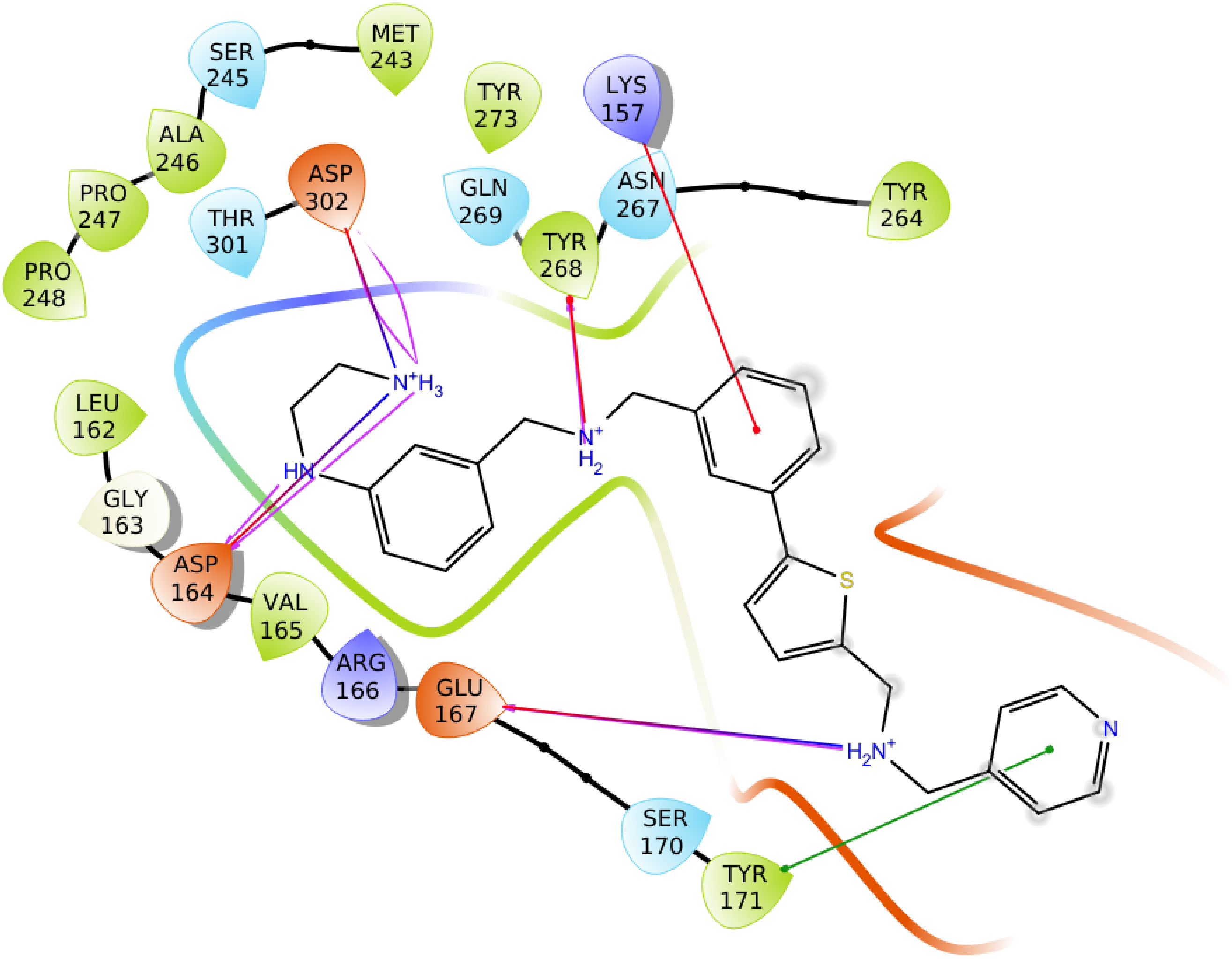

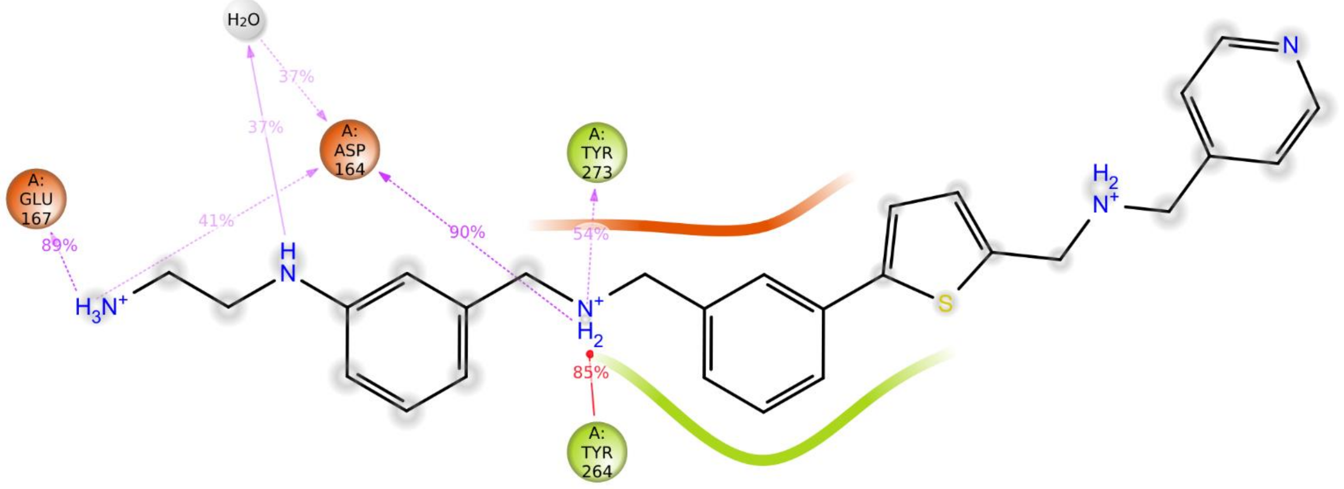
H2O
37%
37%
A: ASP 164
A: GLU 167
41%
89%
90%
A: TYR 273
54%
85%
A: TYR 264
N
S

Palinavir in H73

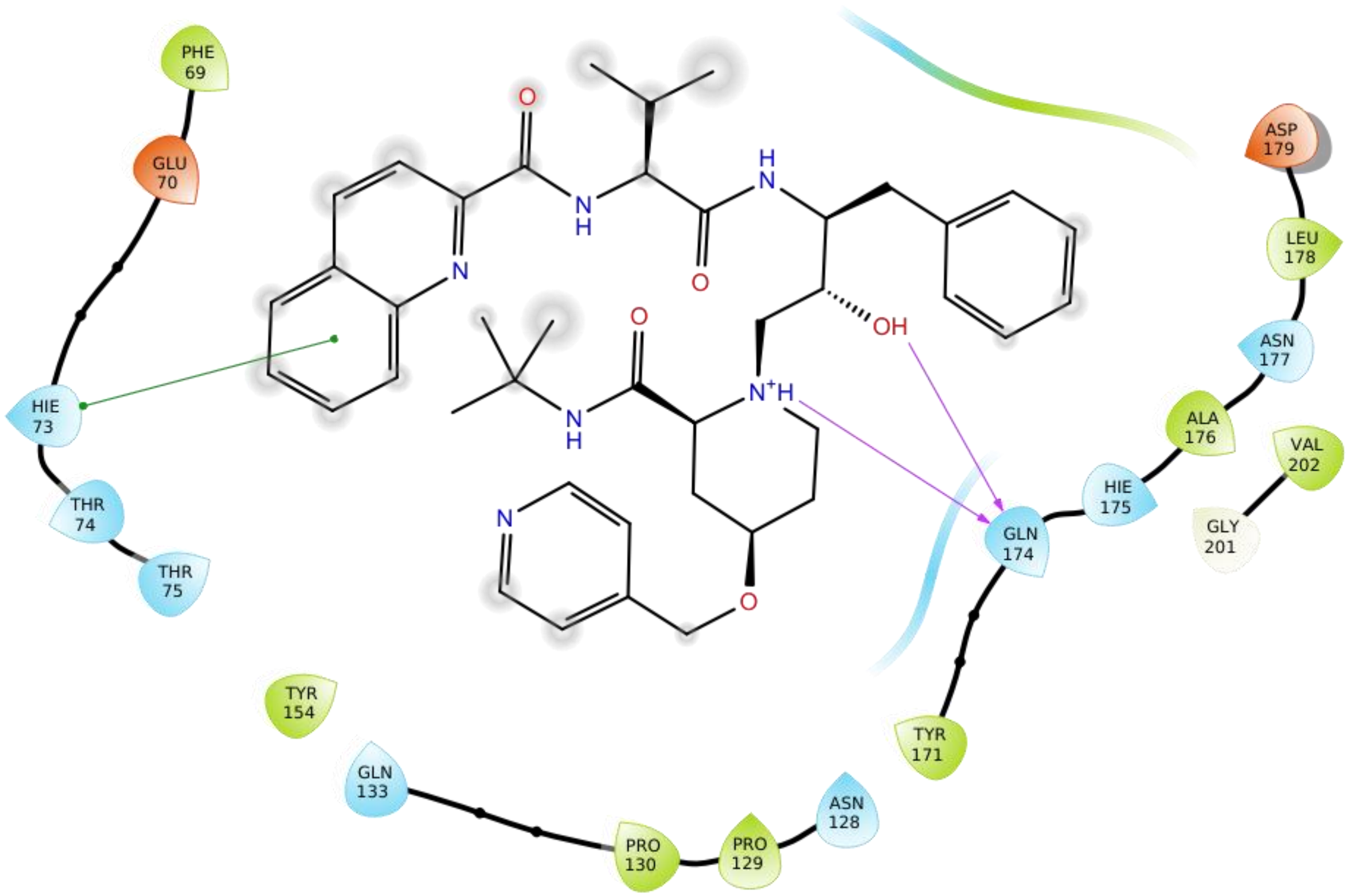

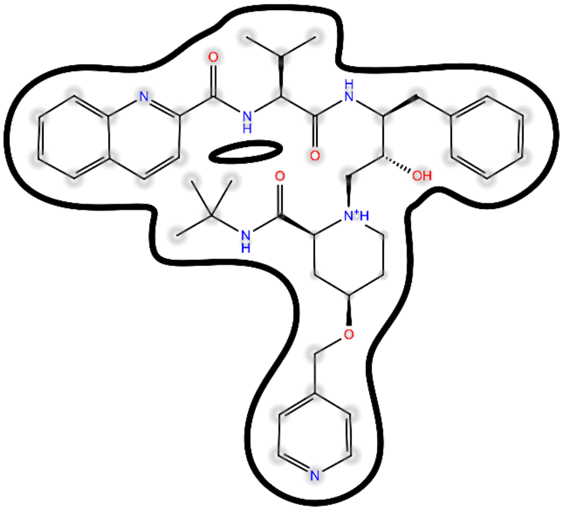

N
O
N
H
H
N
O
OH
O
N
H
N⁺H
O
N

Indinavir in H73 (State of protonation 1)

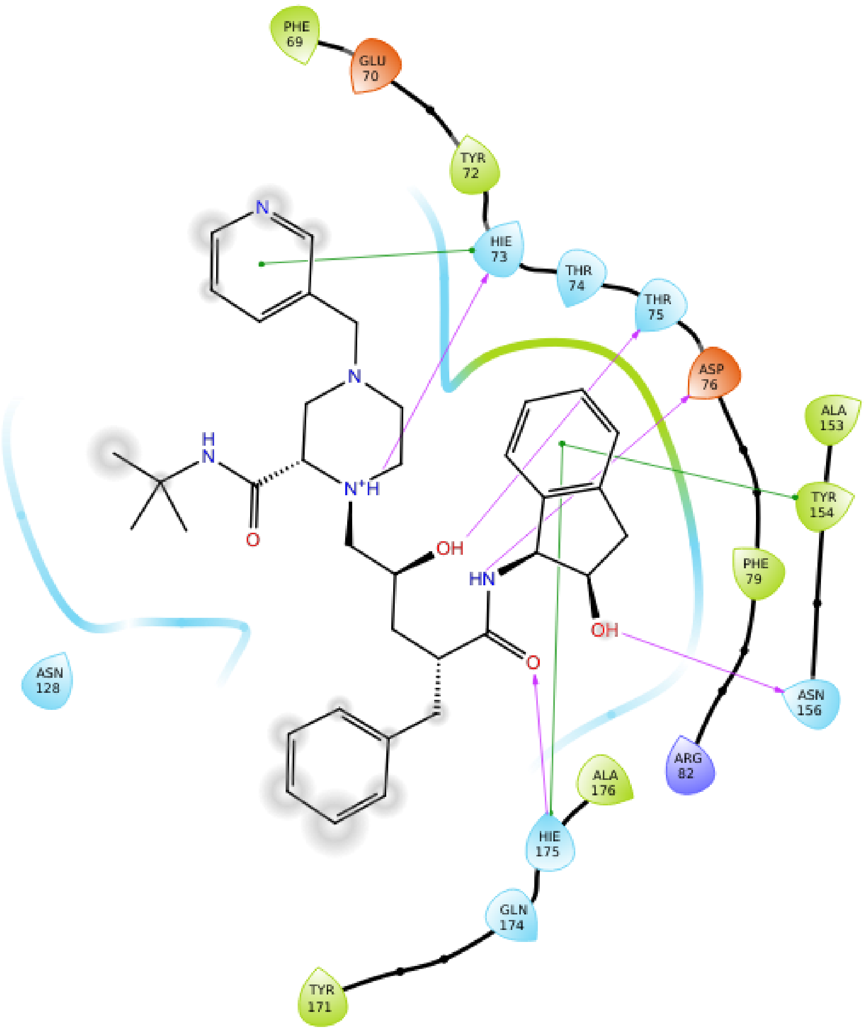

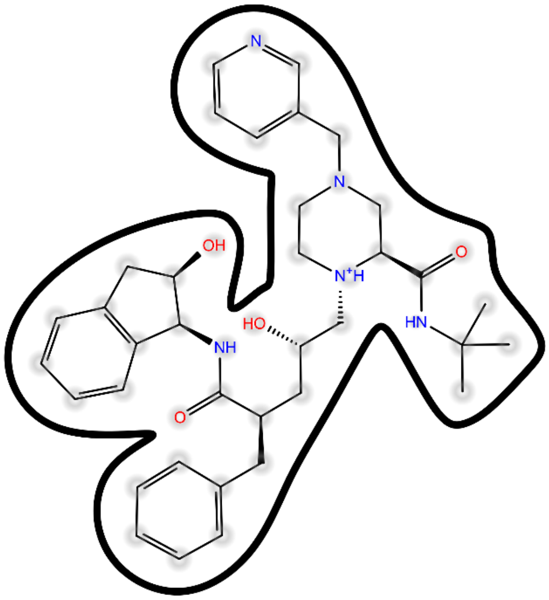
N
N
N+H
O
HN
HO
OH
NH
O

Indinavir in H73 (State of protonation 2)

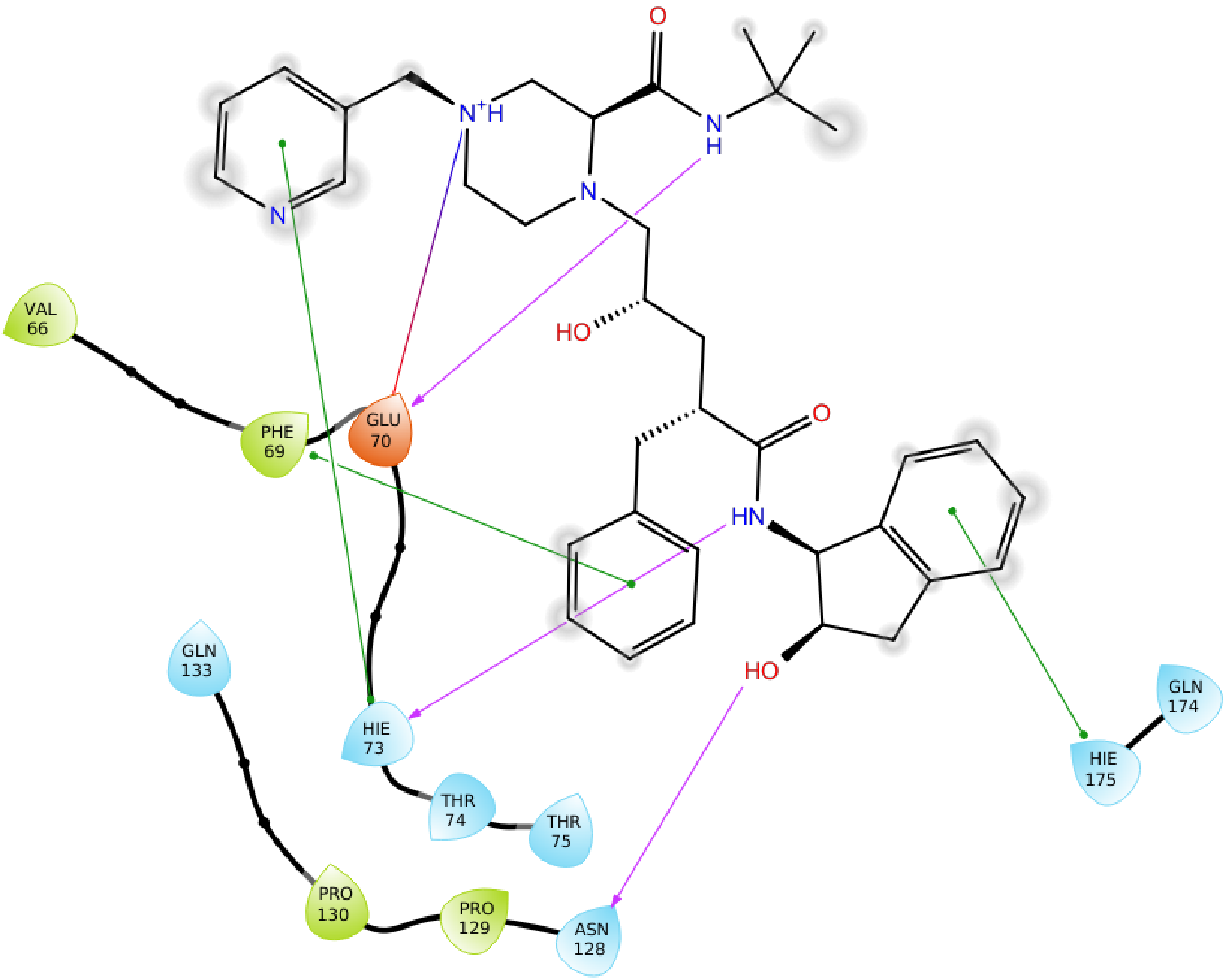

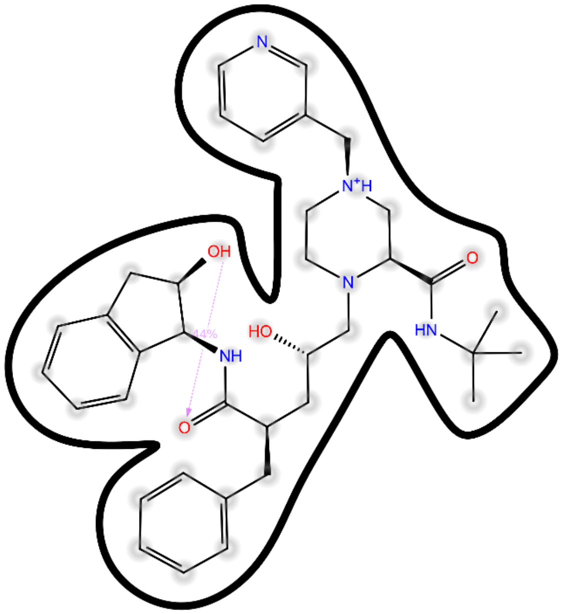

Atazanivir in H73

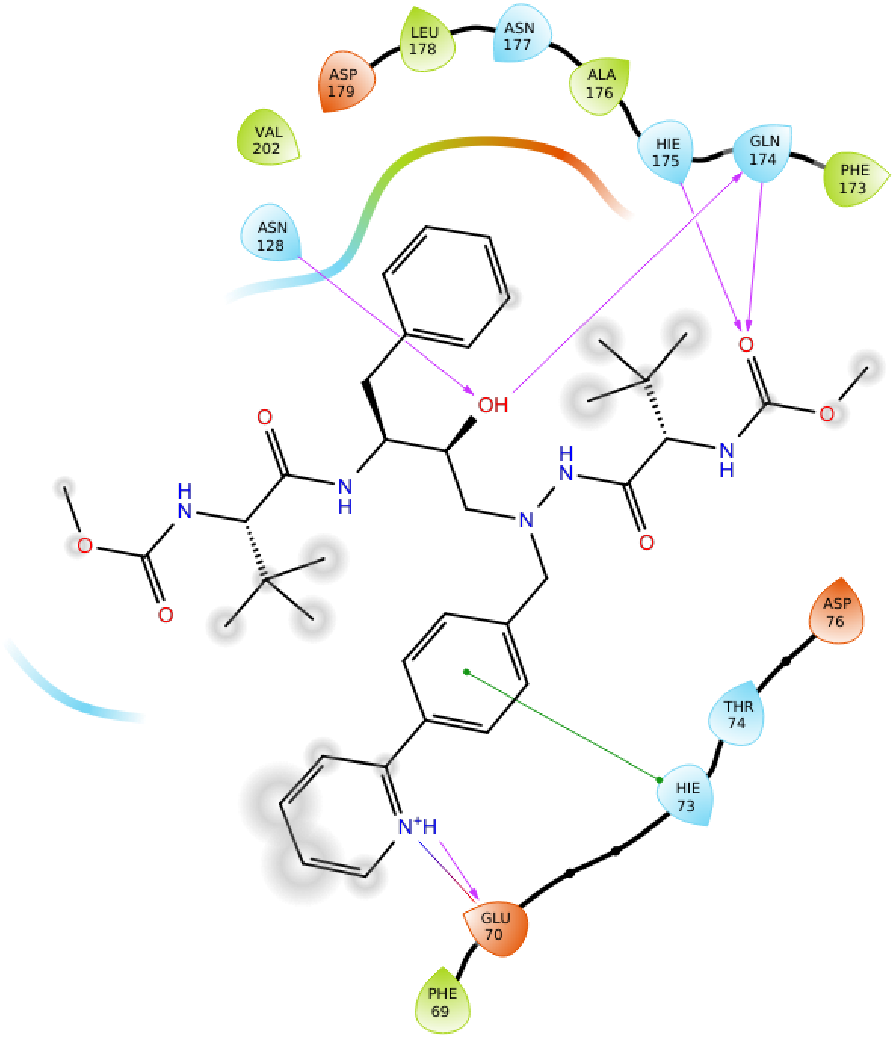

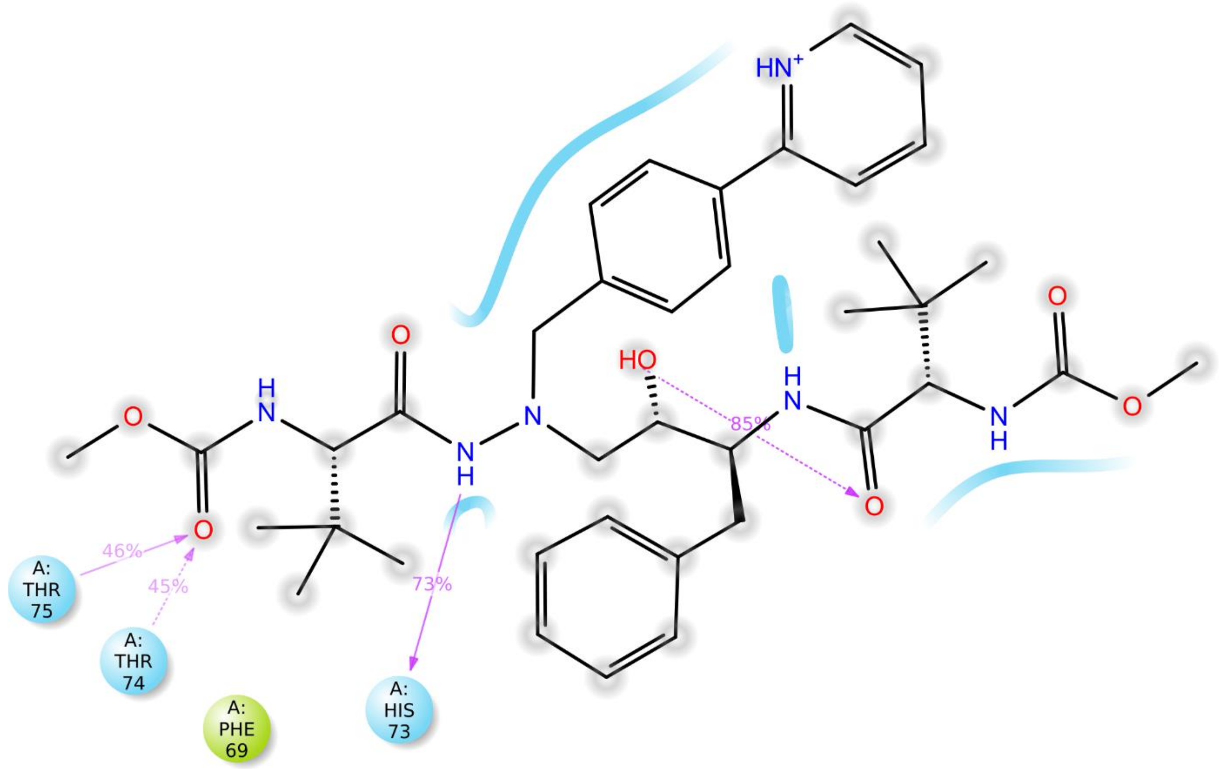

HN+
O
HO
H
N
H
N
O
N
H
N
85%
O
O
O
N
H
O
46%
45%
73%
O
A:
THR
75
A:
THR
74
A:
PHE
69
A:
HIS
73

Compound Pred10 in H73

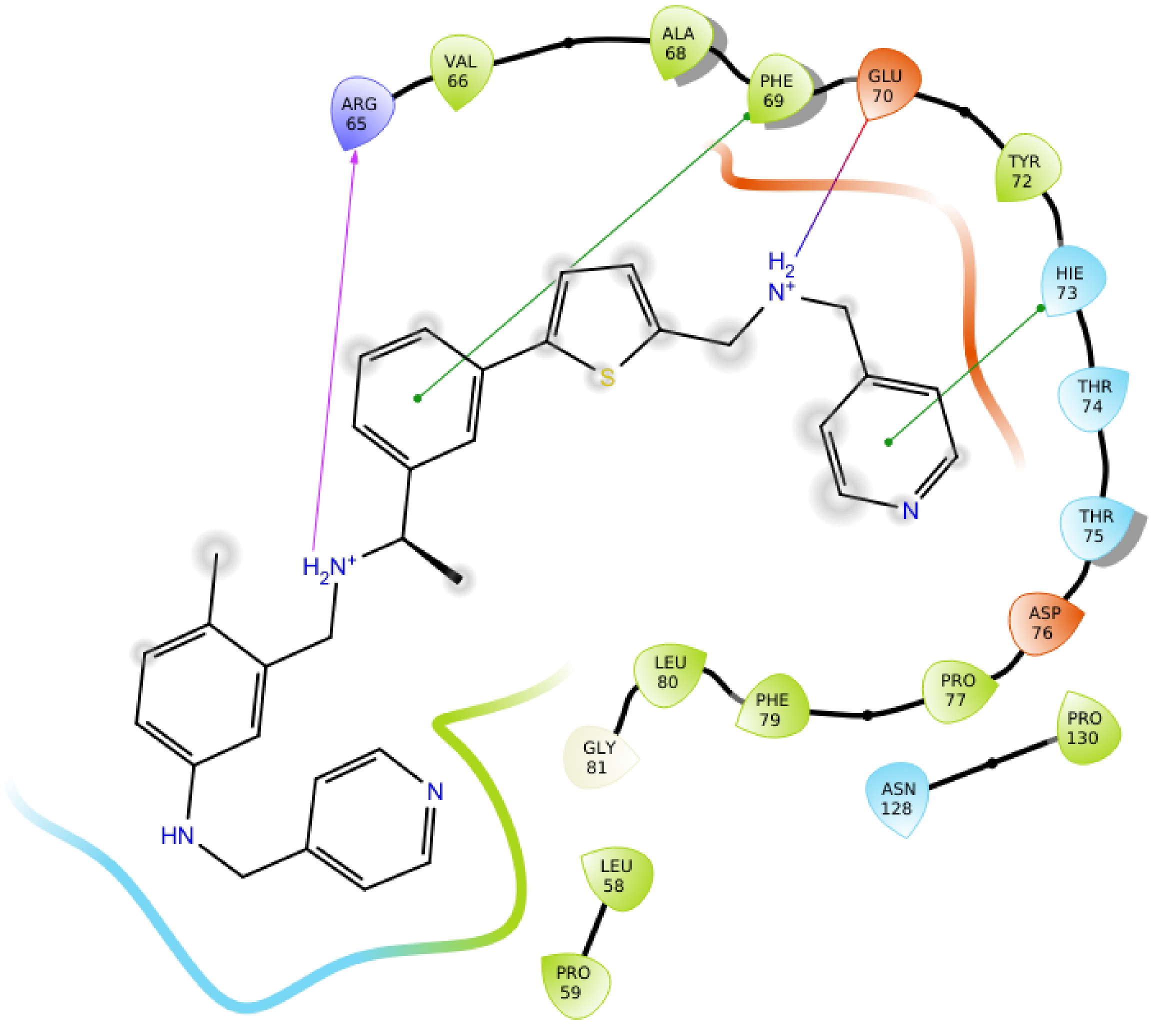

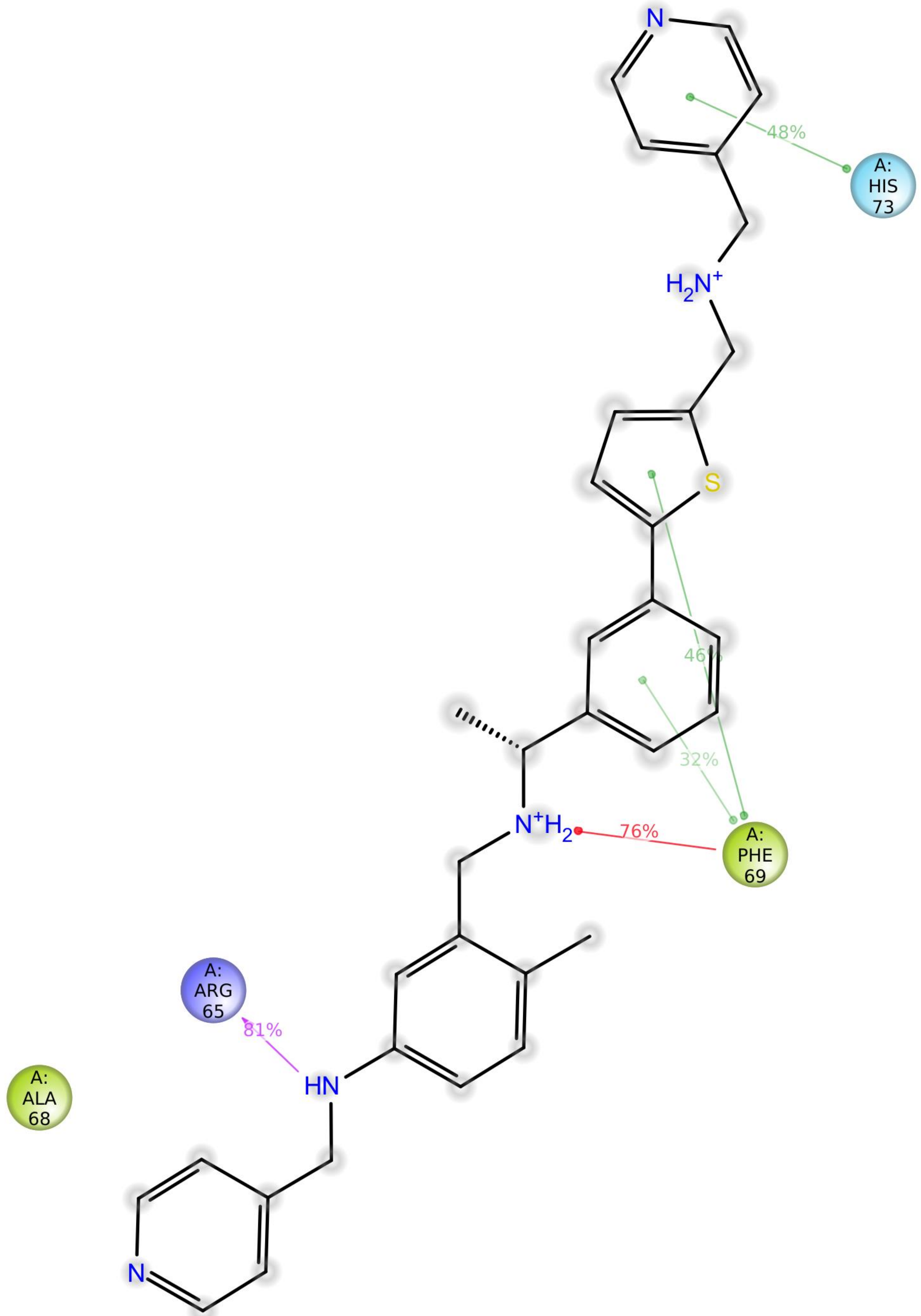
N
48%
A:
HIS
73
H2N+
S
46%
32%
N+H2
76%
A:
PHE
69
A:
ARG
65
81%
HN
A:
ALA
68
N

Compound Pred14 in H73

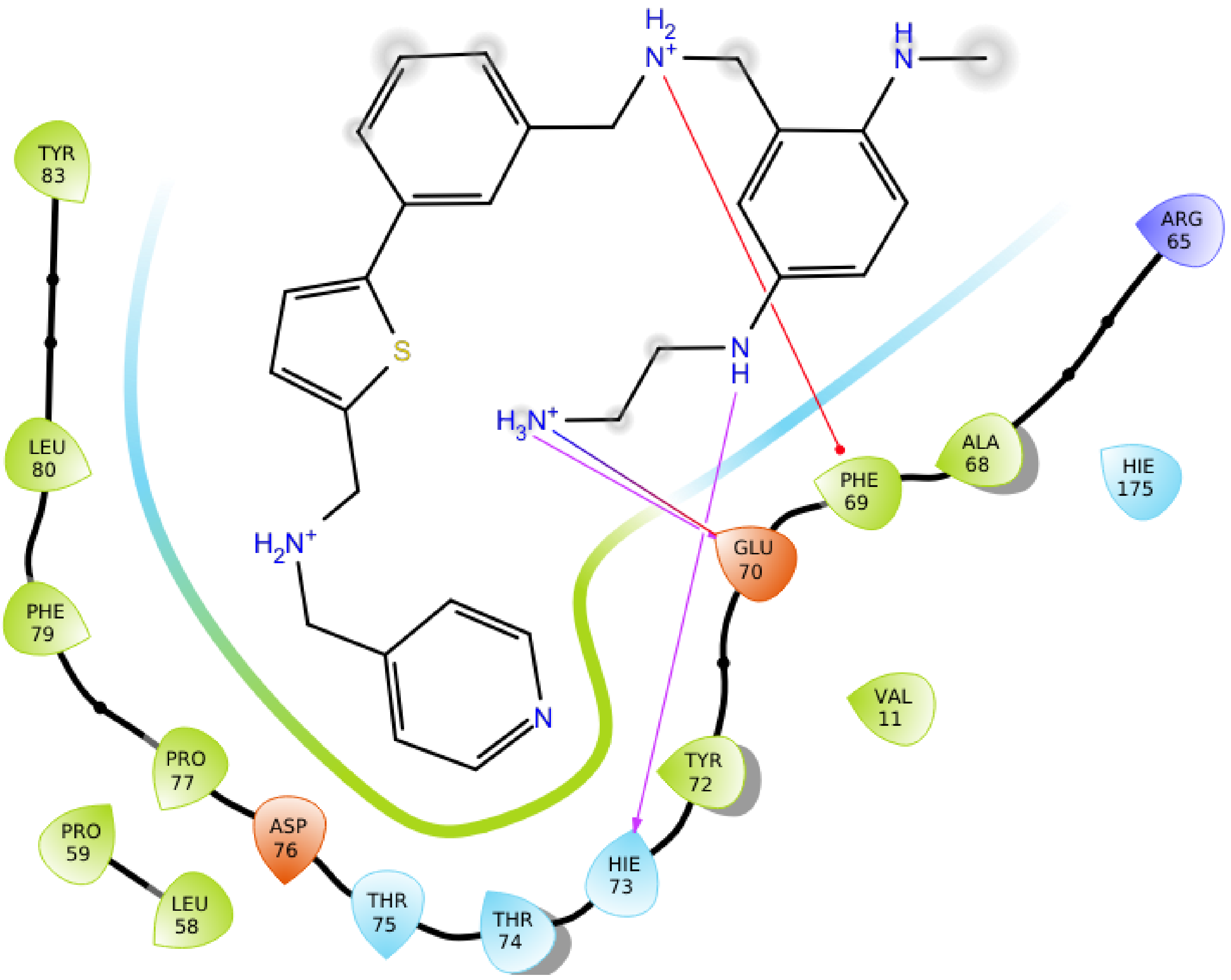

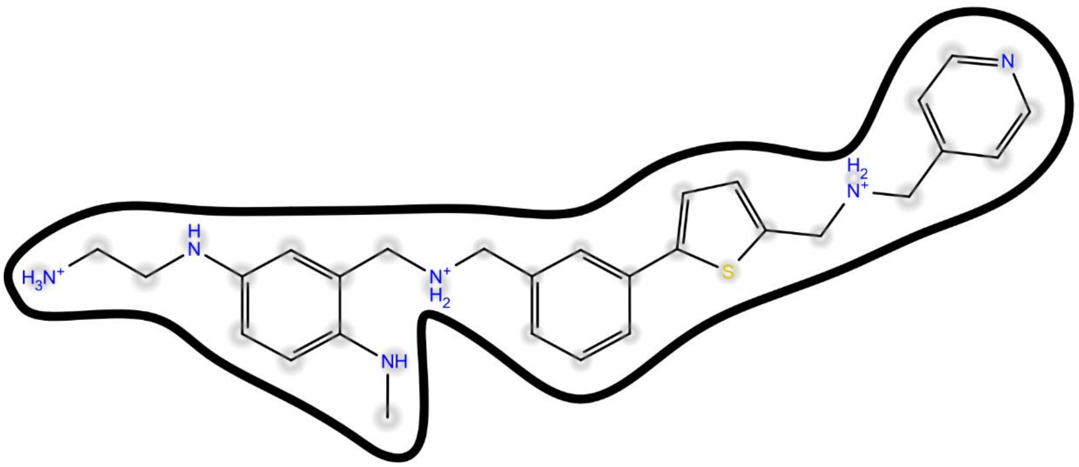

Compound Pred 15 in H73

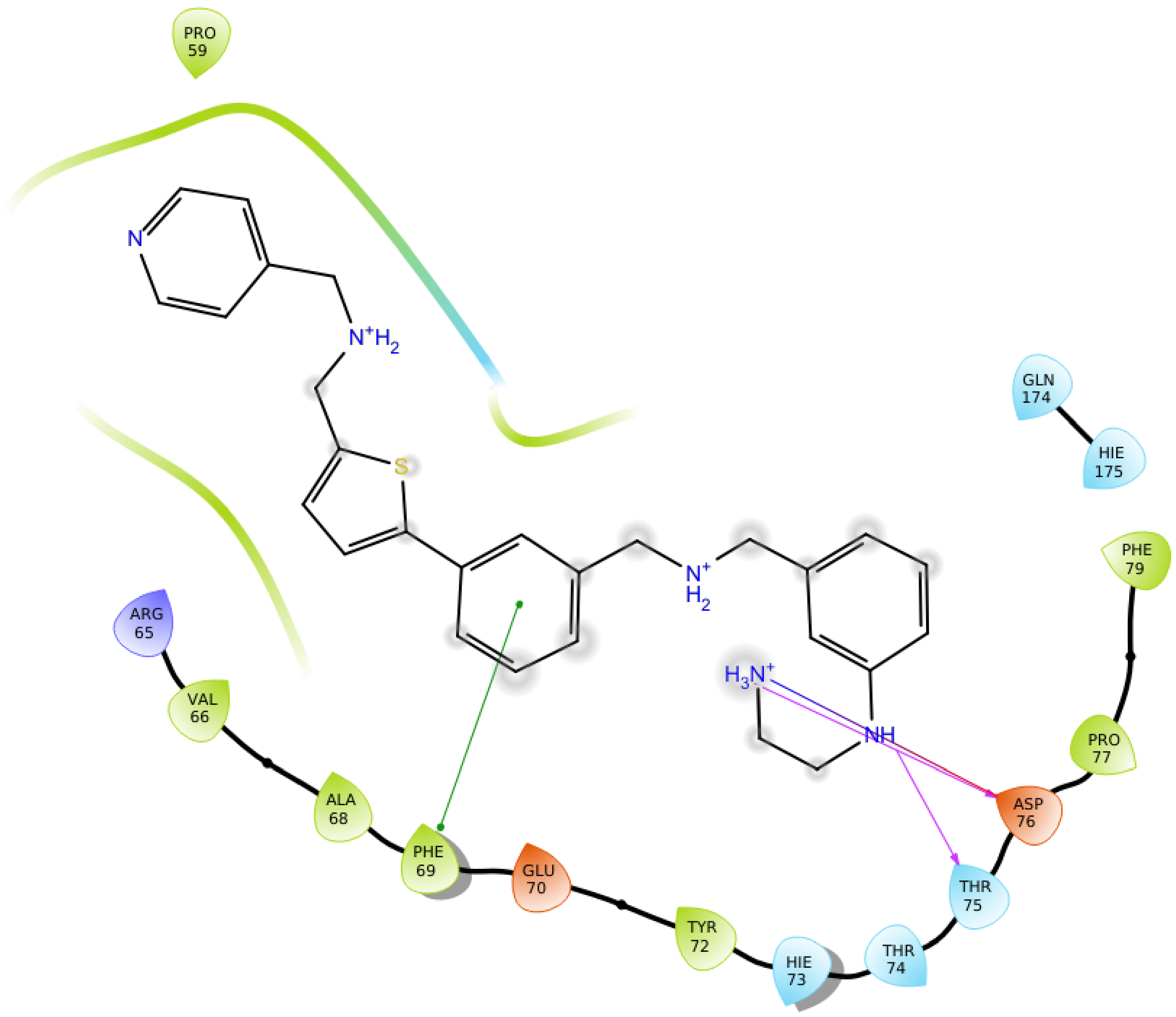

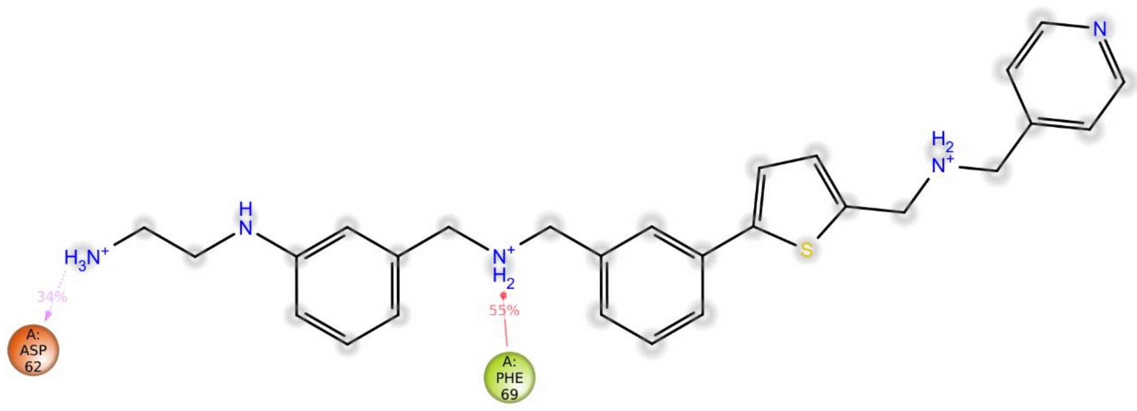

Compound Pred2 in H73

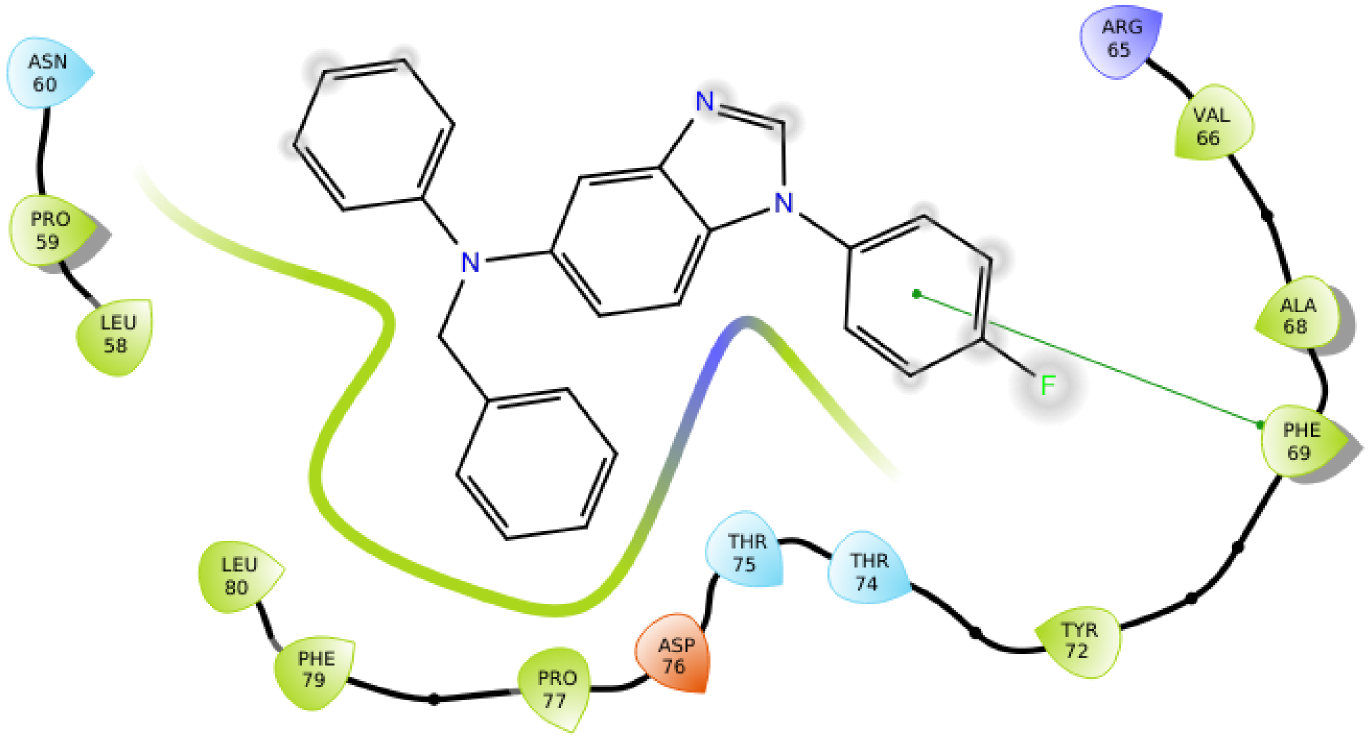

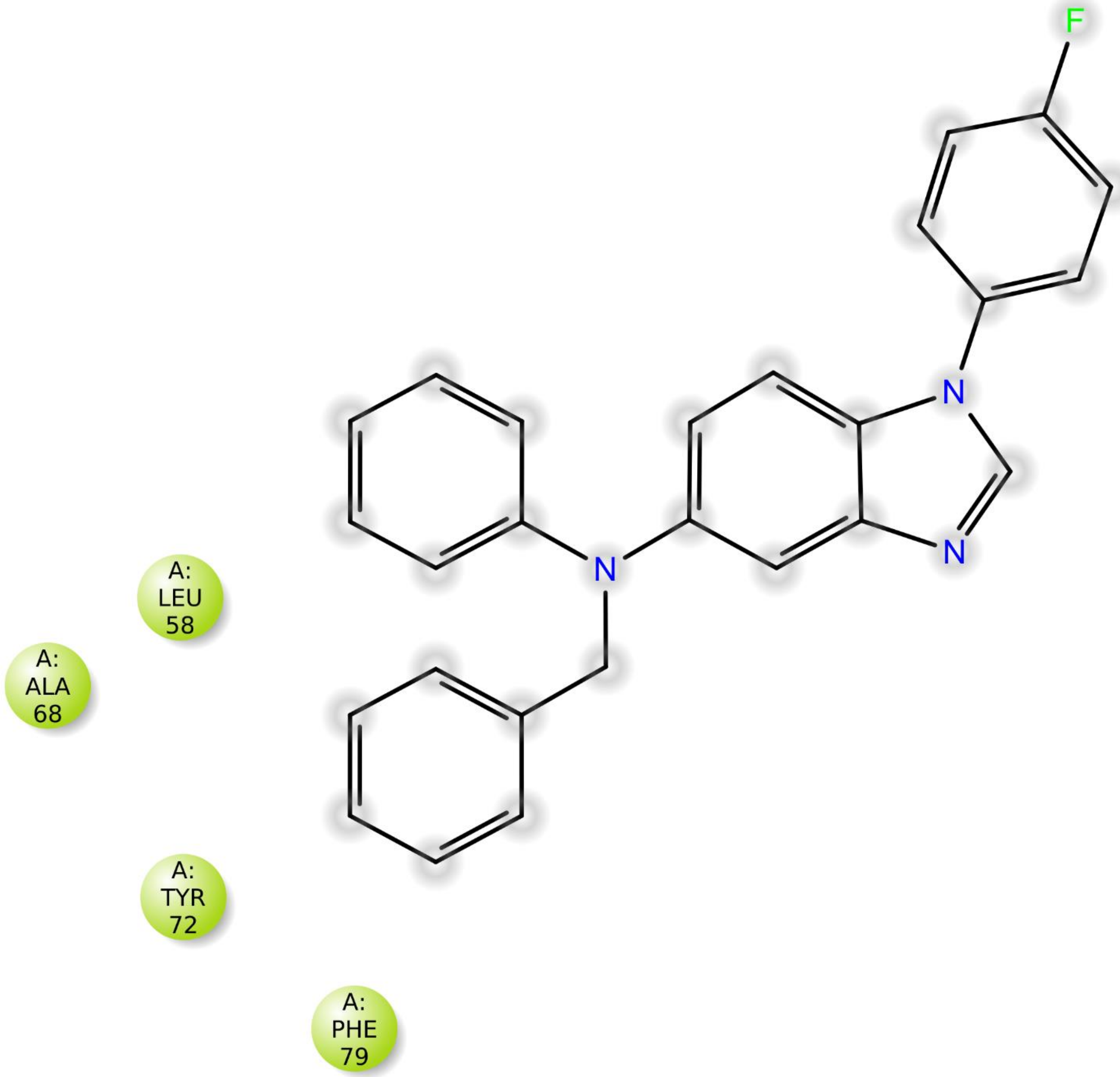
F
N
N
N
A:
LEU
58
A:
ALA
68
A:
TYR
72
A:
PHE
79

Figure S2. The RMSD of the antivirals and designed molecules.

The RMSD of Indinavir in Y268 (State of protonation 1)

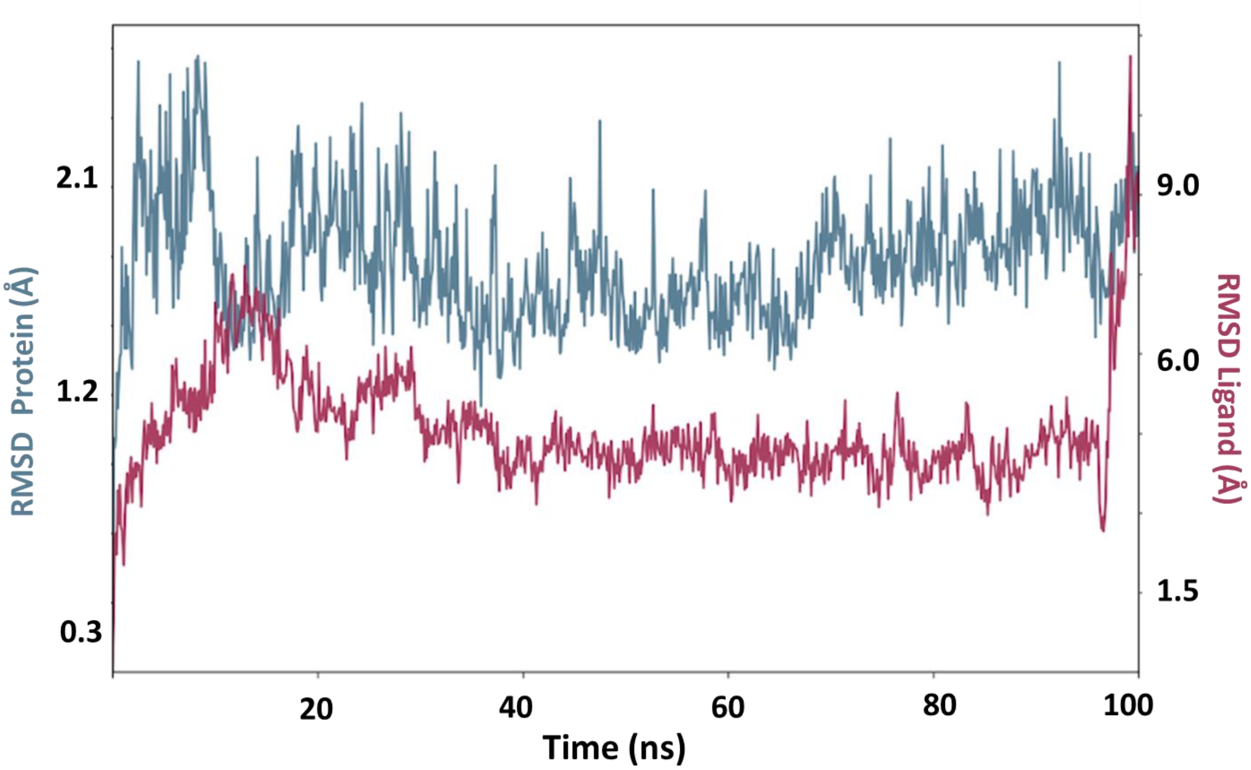

The RMSD of Indinavir in Y268 (State of protonation 2)

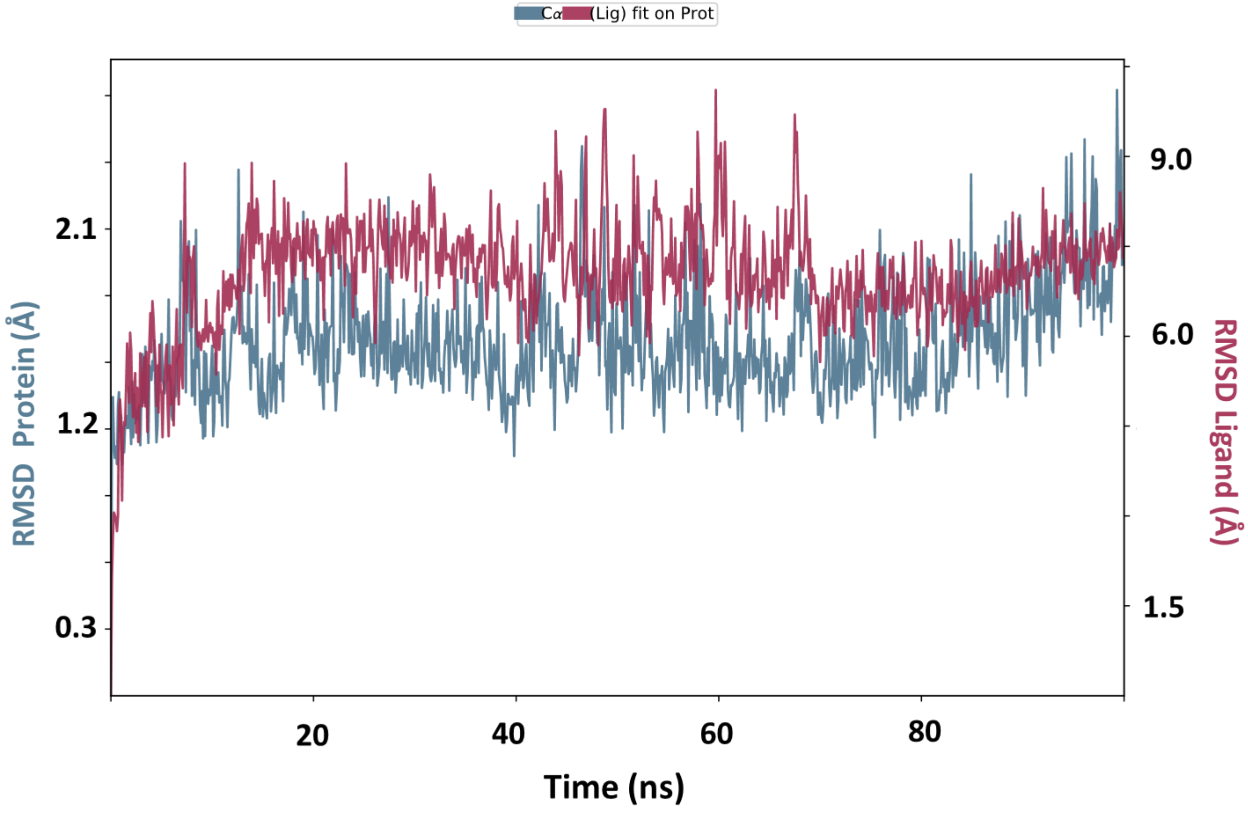

The RMSD of Etravirine in Y268

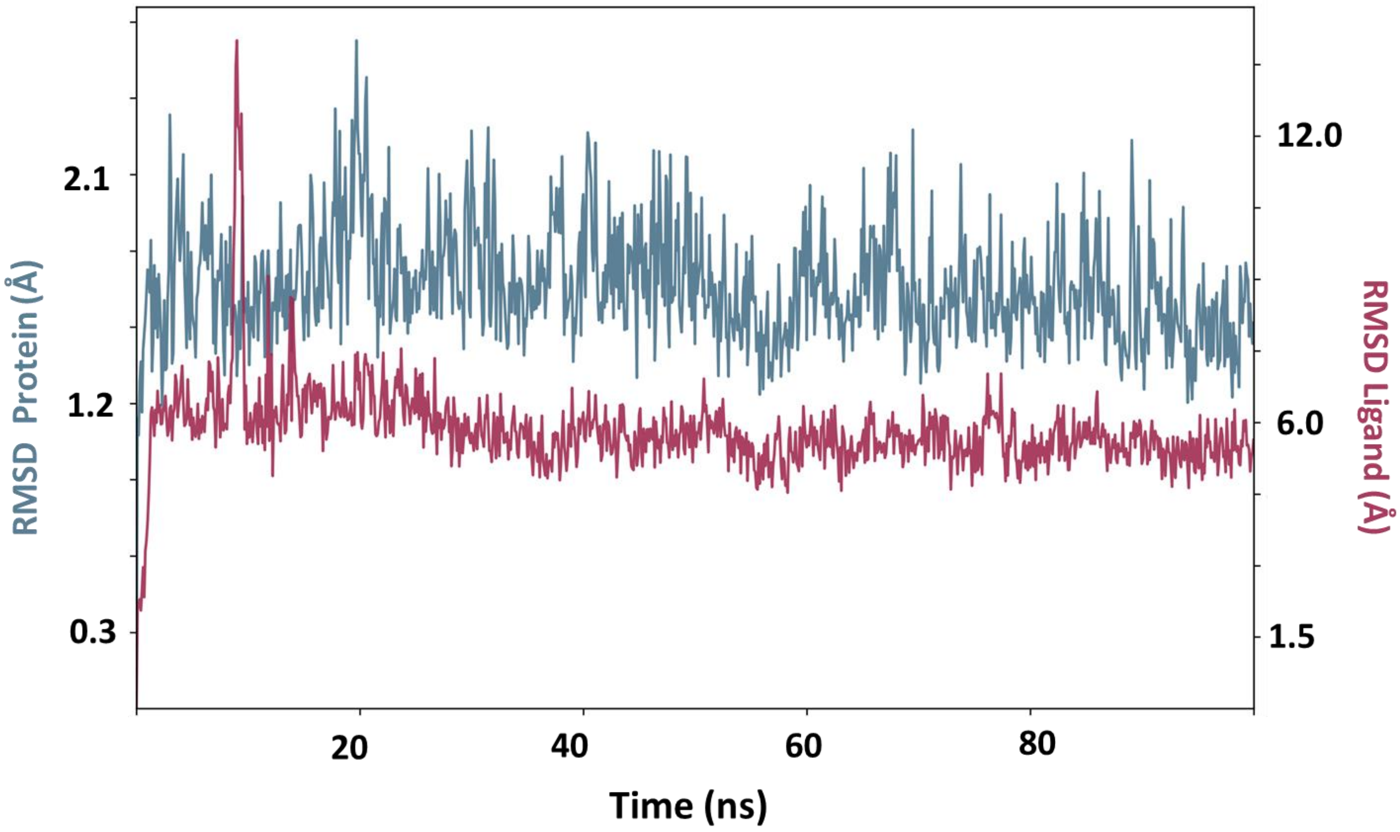

The RMSD of Palinavir in Y268

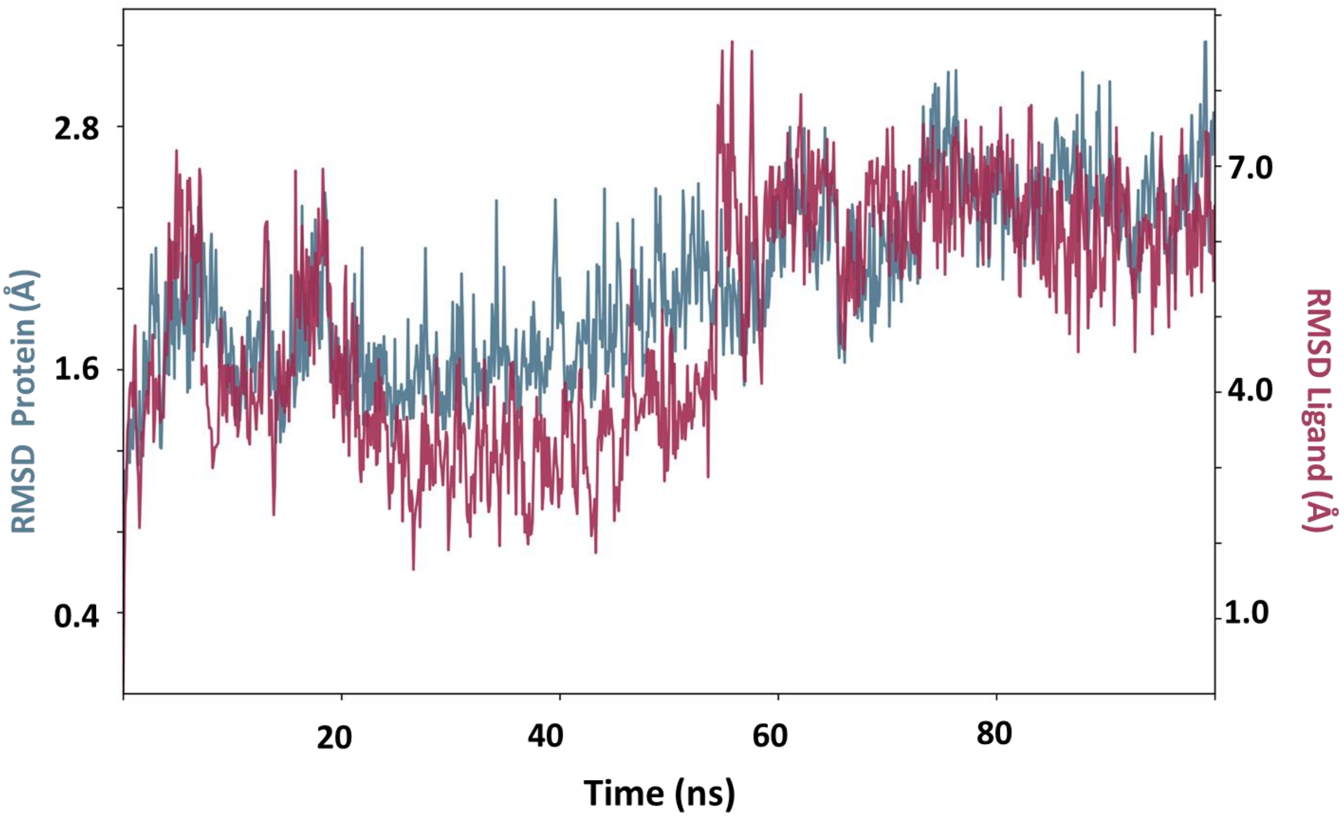

The RMSD of Lopinavir in C111

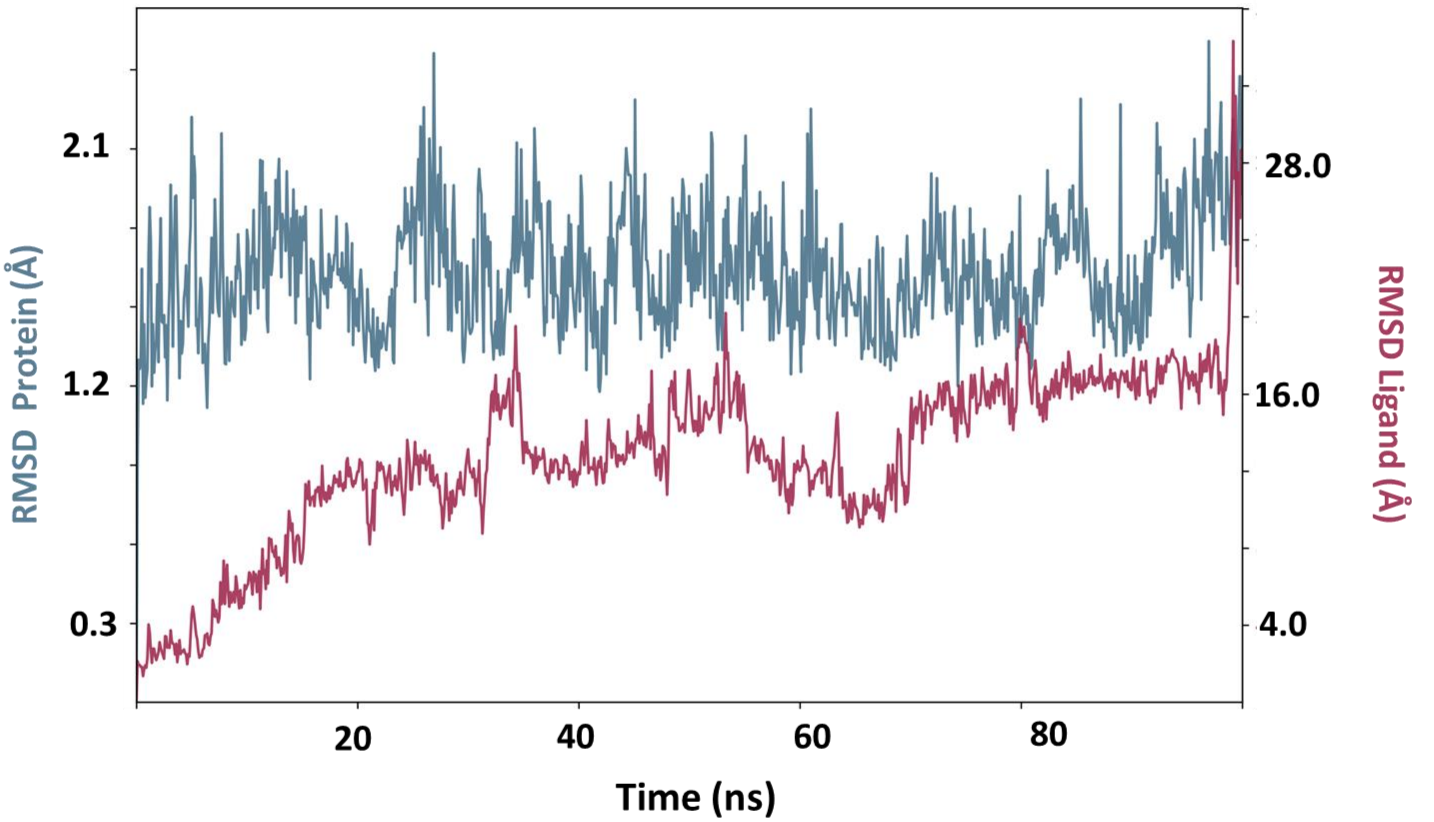

The RMSD of Indinavir in C111 (State of protonation 1)

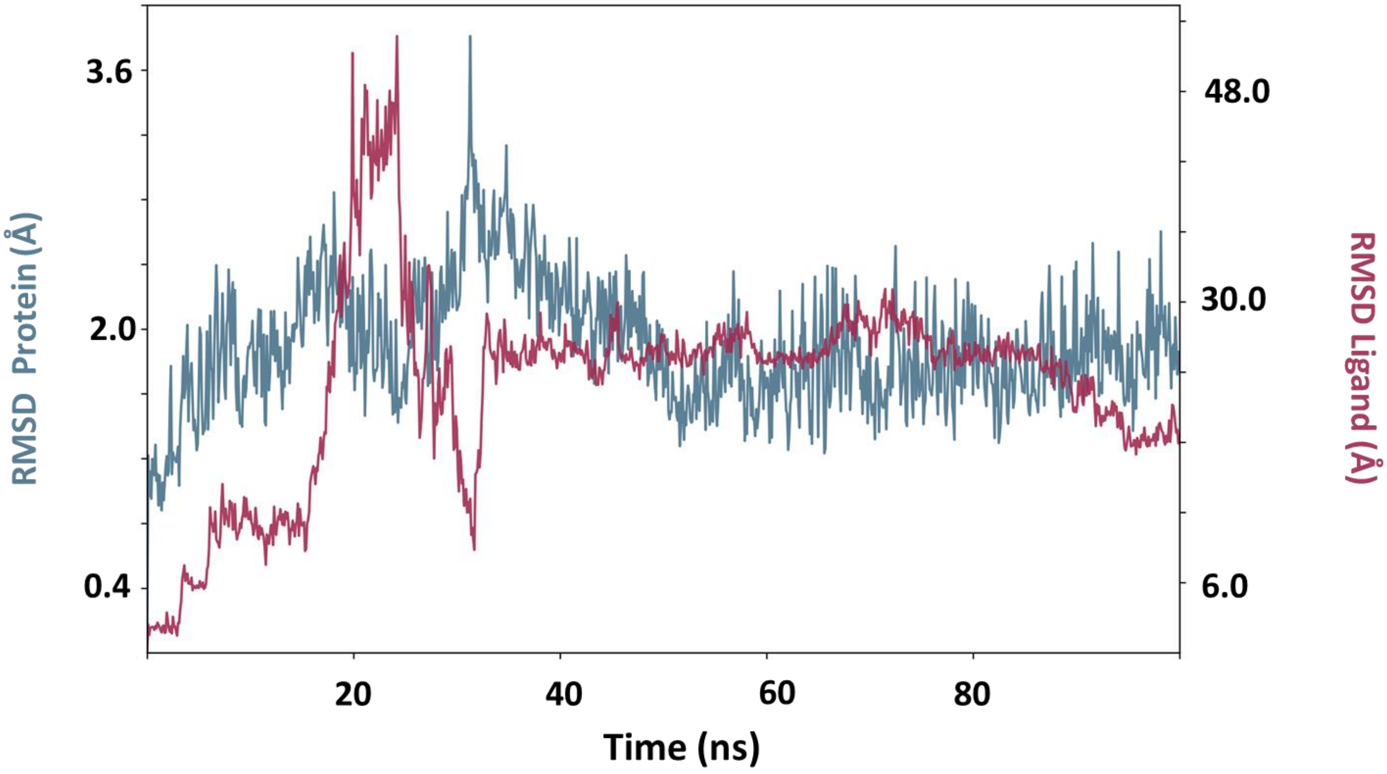

The RMSD of Indinavir in C111 (State of protonation 2)

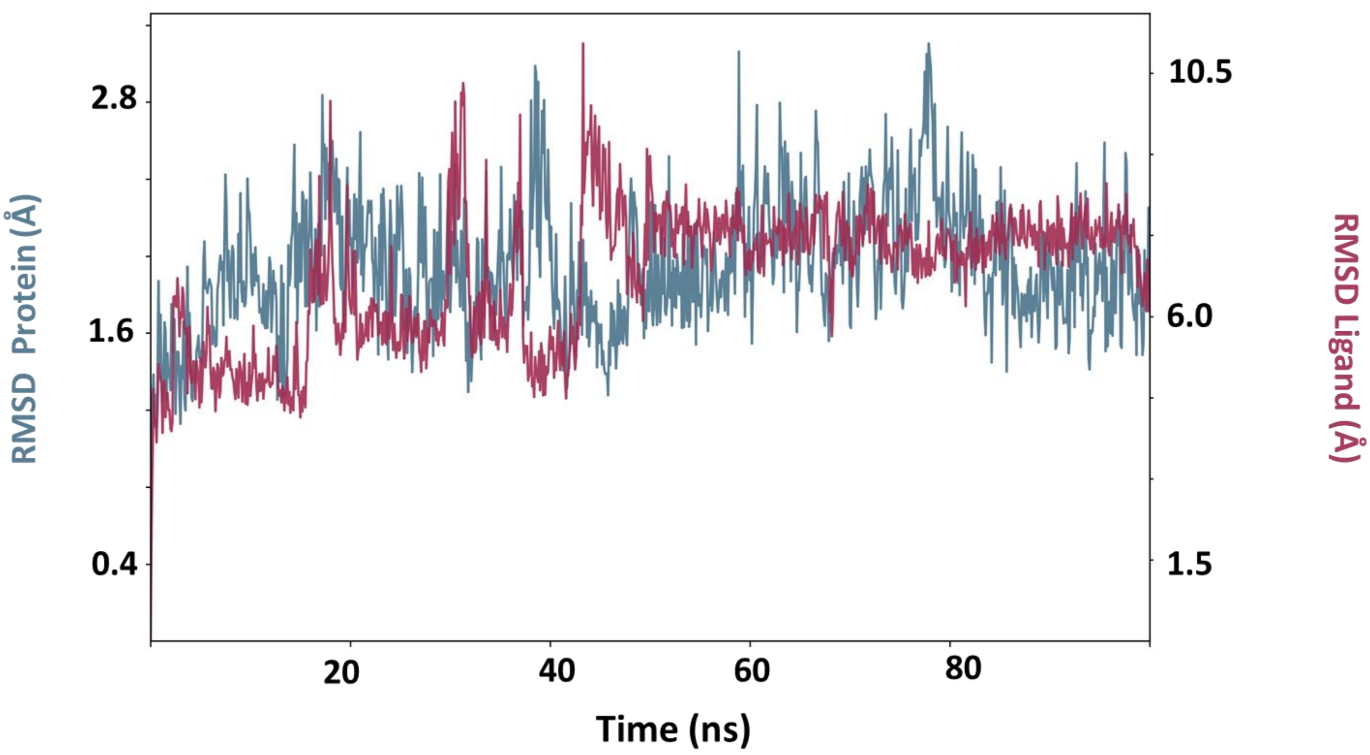

The RMSD of Etravirine in C111

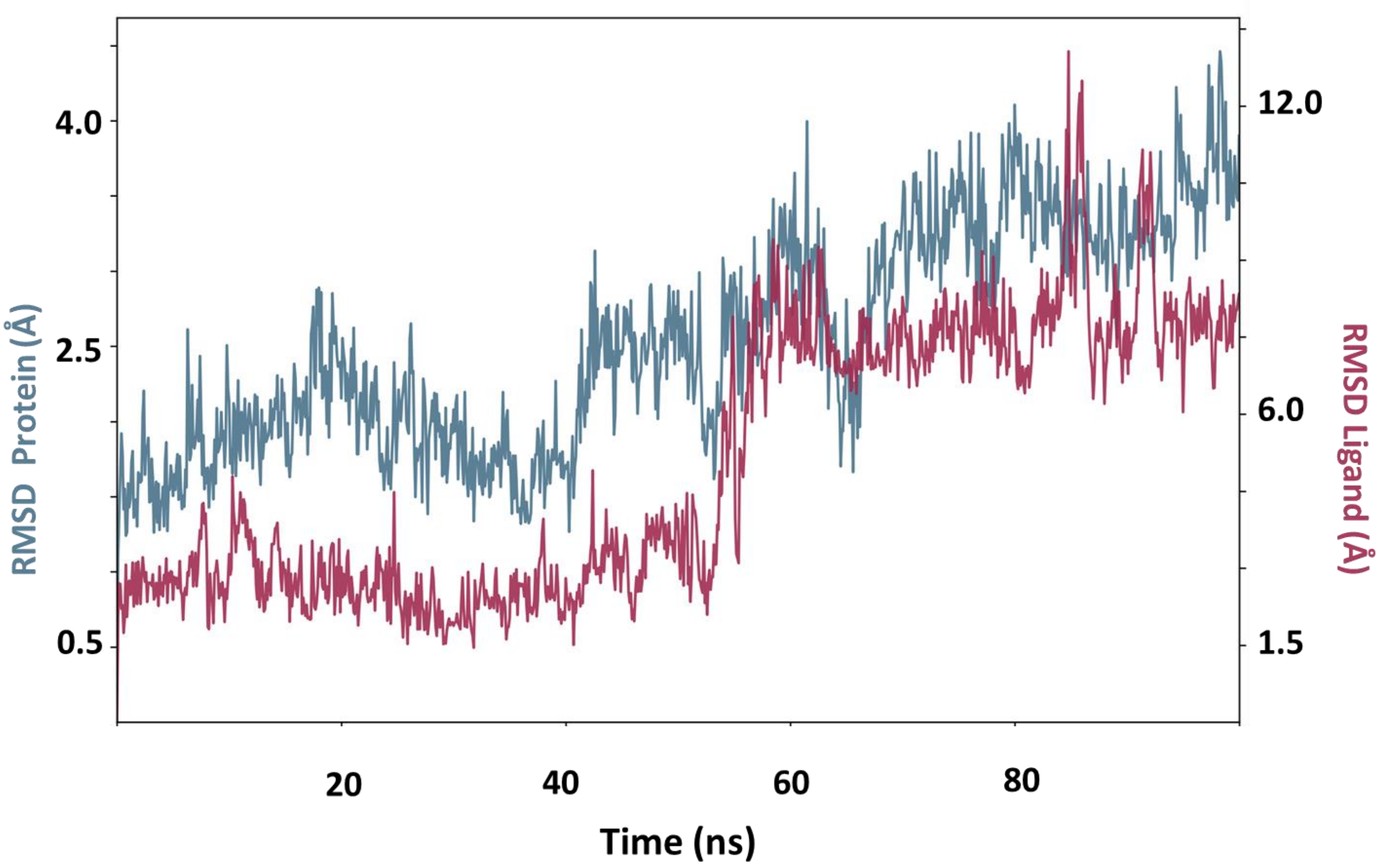

The RMSD of Indinavir in H73 (State of protonation 1)

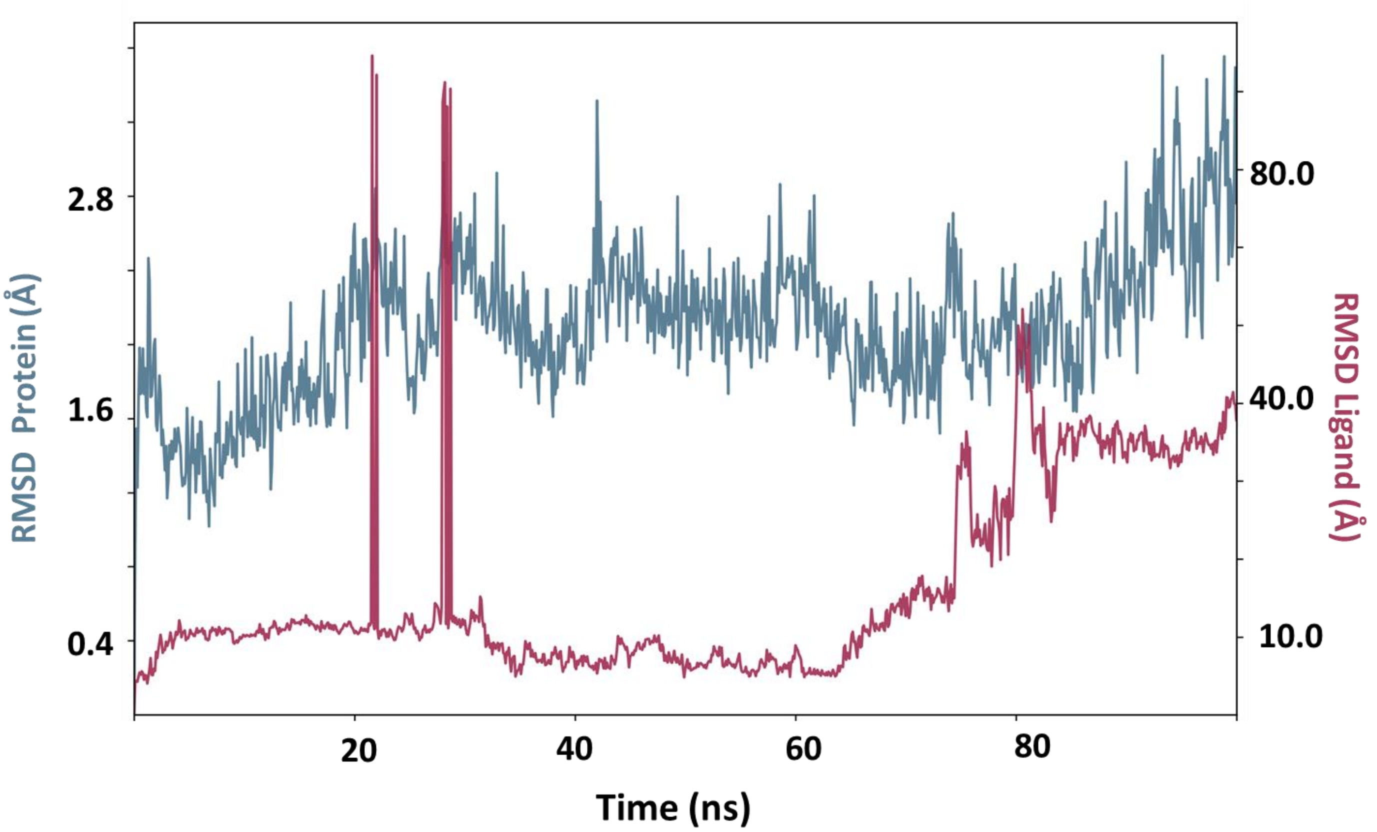

The RMSD of Indinavir in H73 (State of protonation 2)

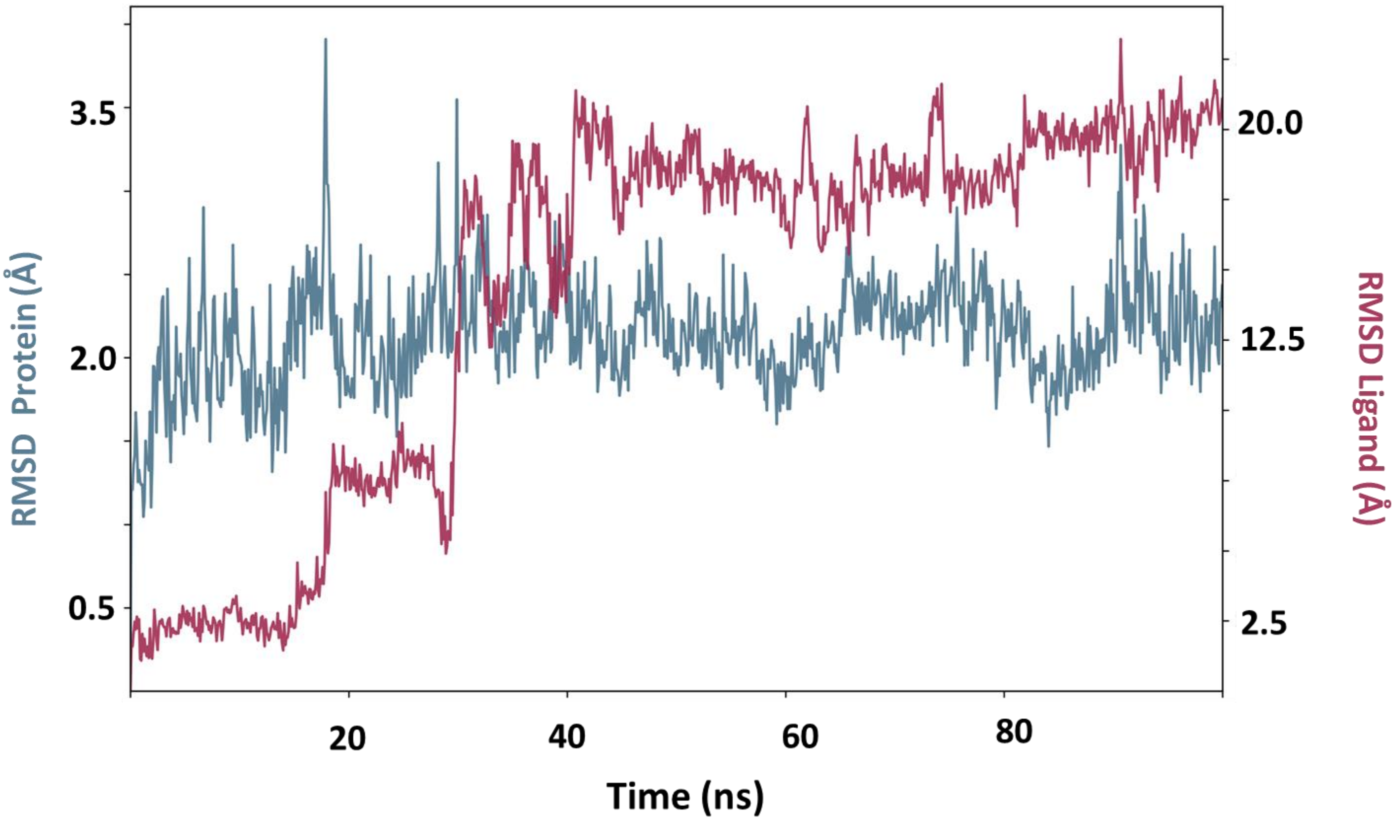

The RMSD of Atazanavir in H73

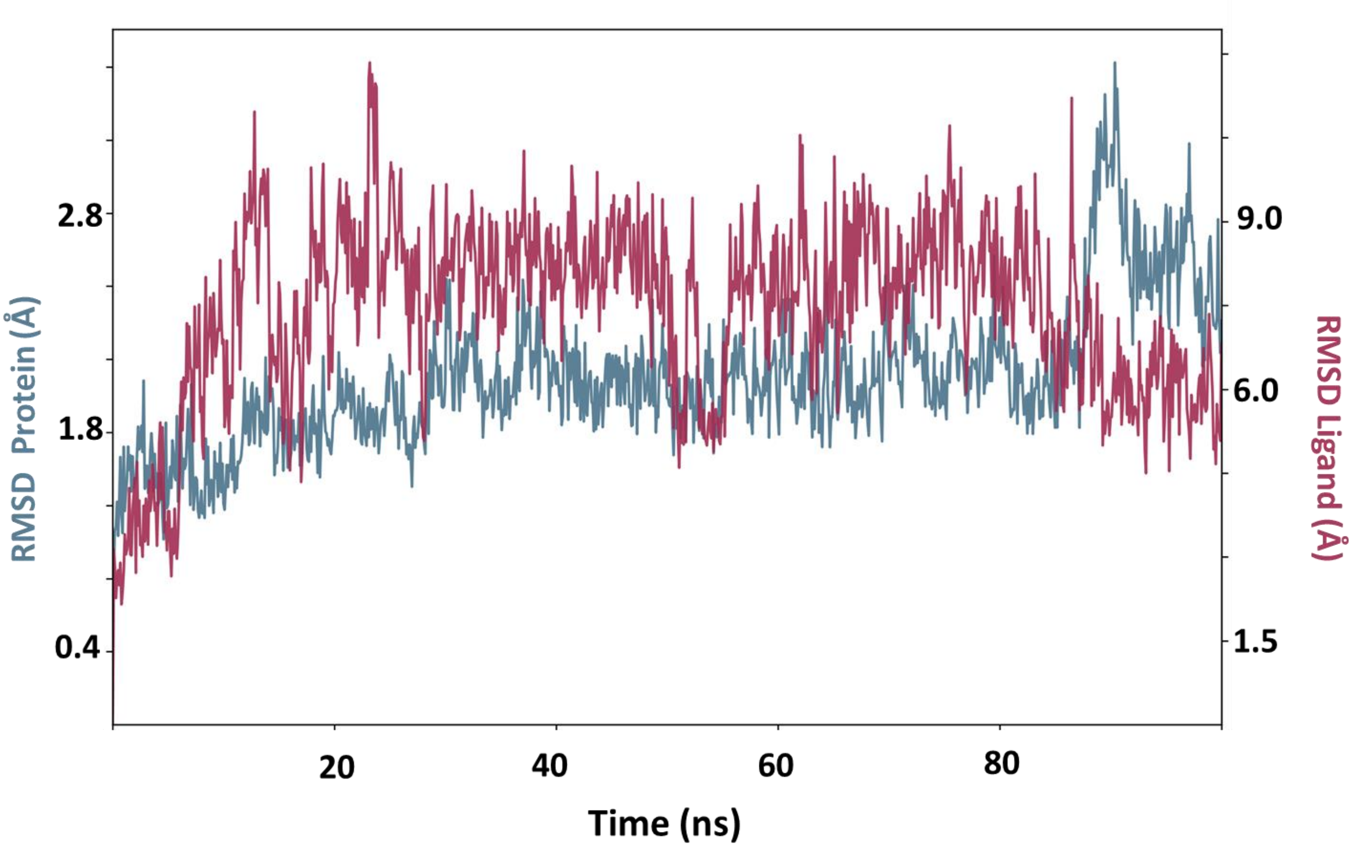

The RMSD of Palinavir in H73

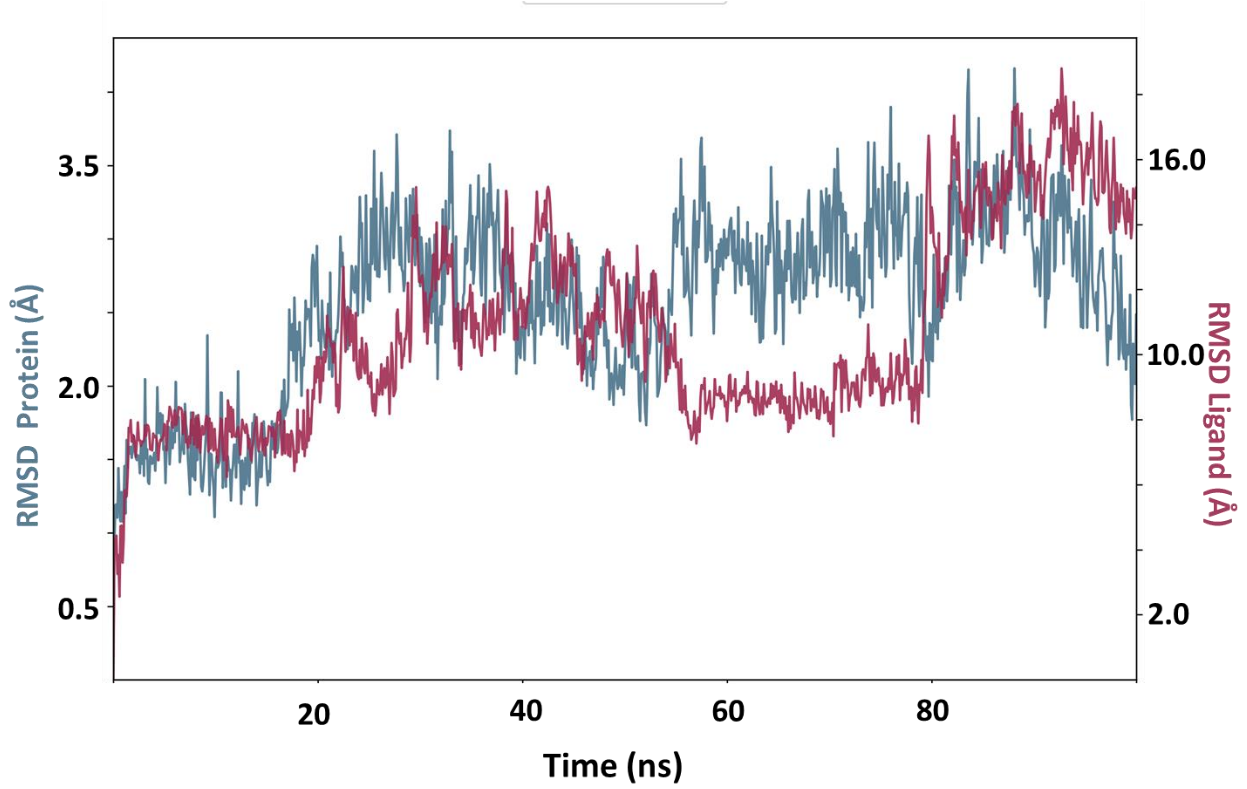

The RMSD of compound Pred14 in Y268

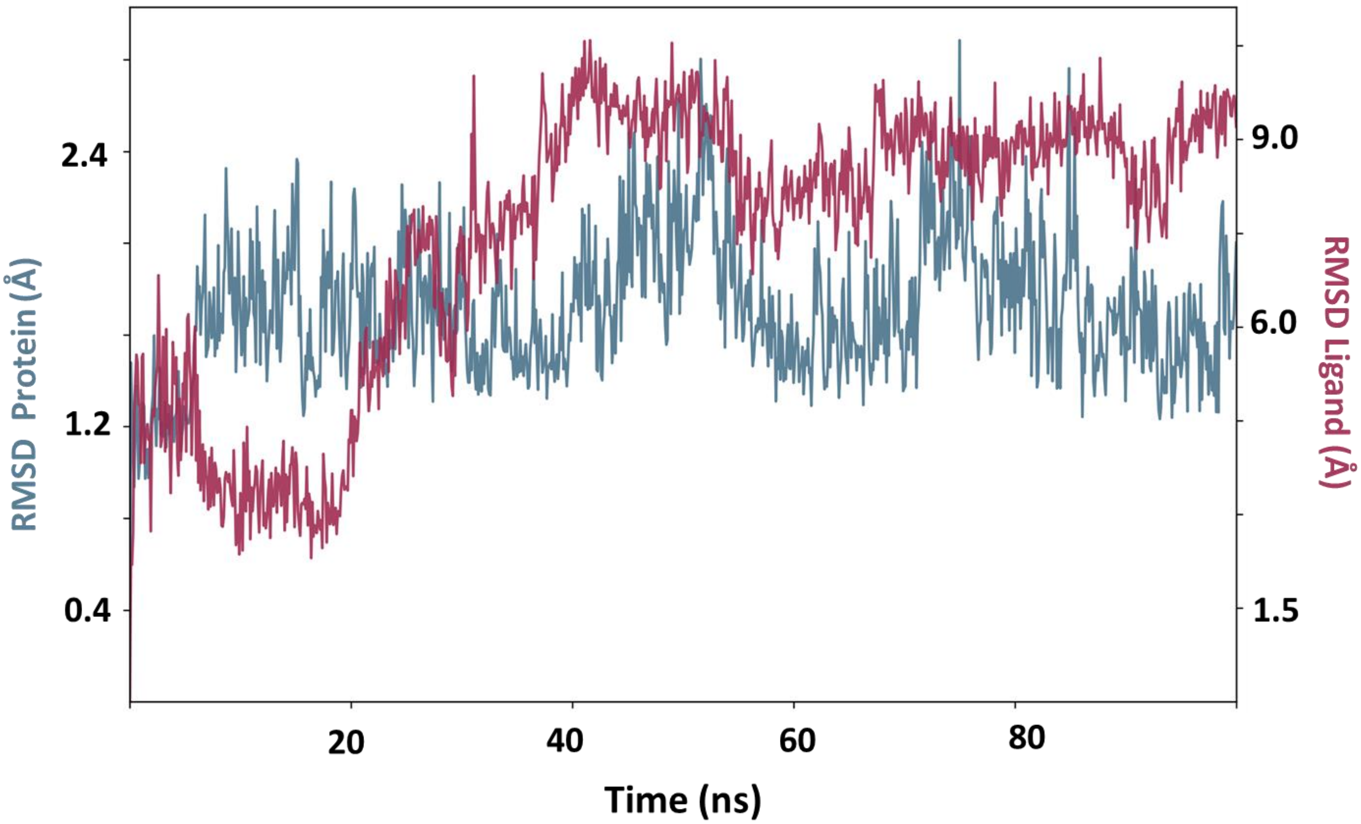

The RMSD of compound Pred15 in Y268

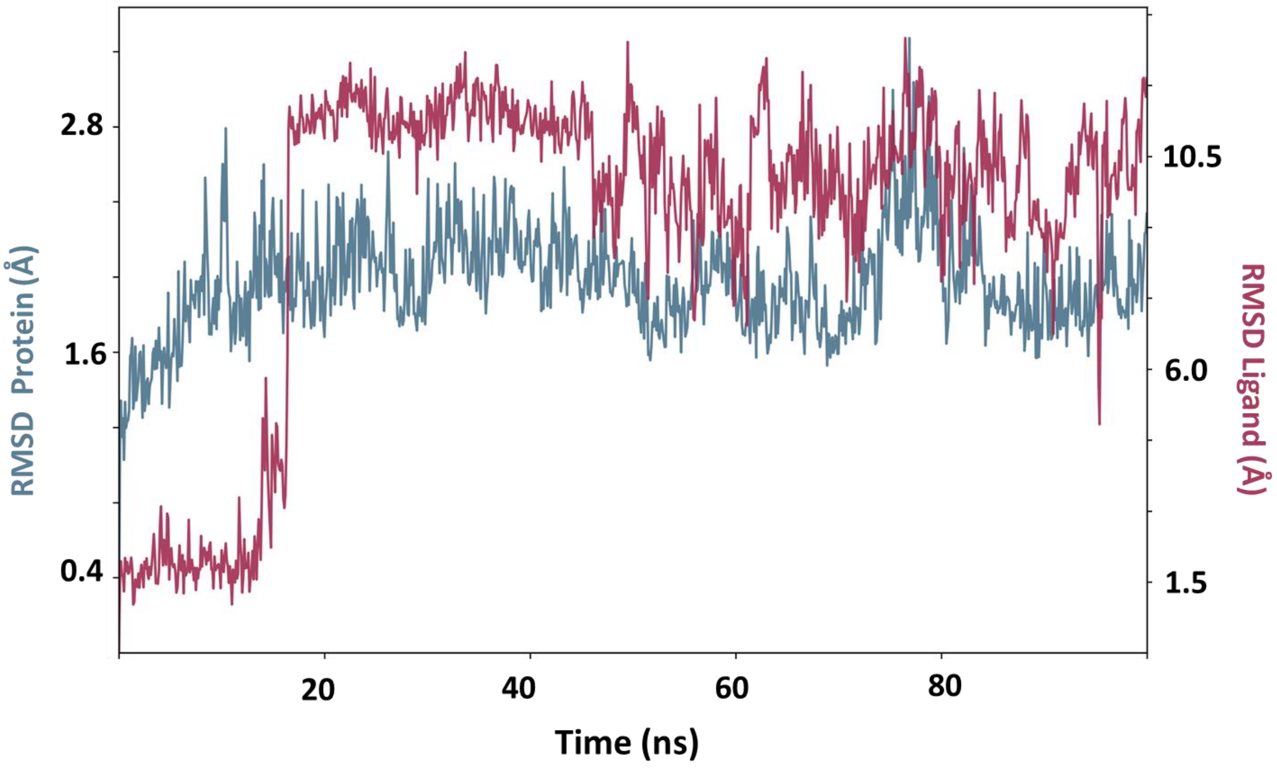

The RMSD of compound Pred12 in Y268

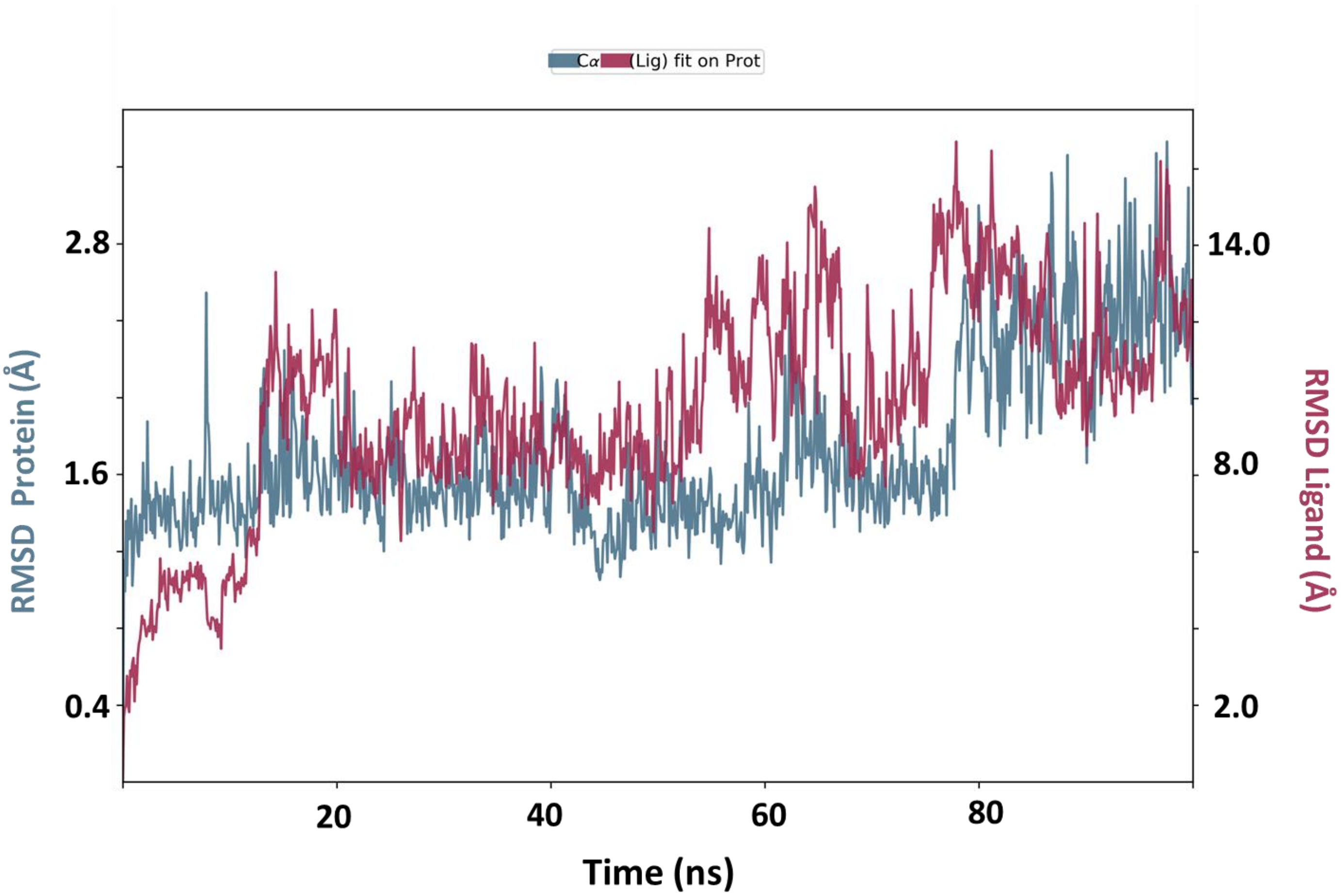

The RMSD of compound Pred10 in Y268

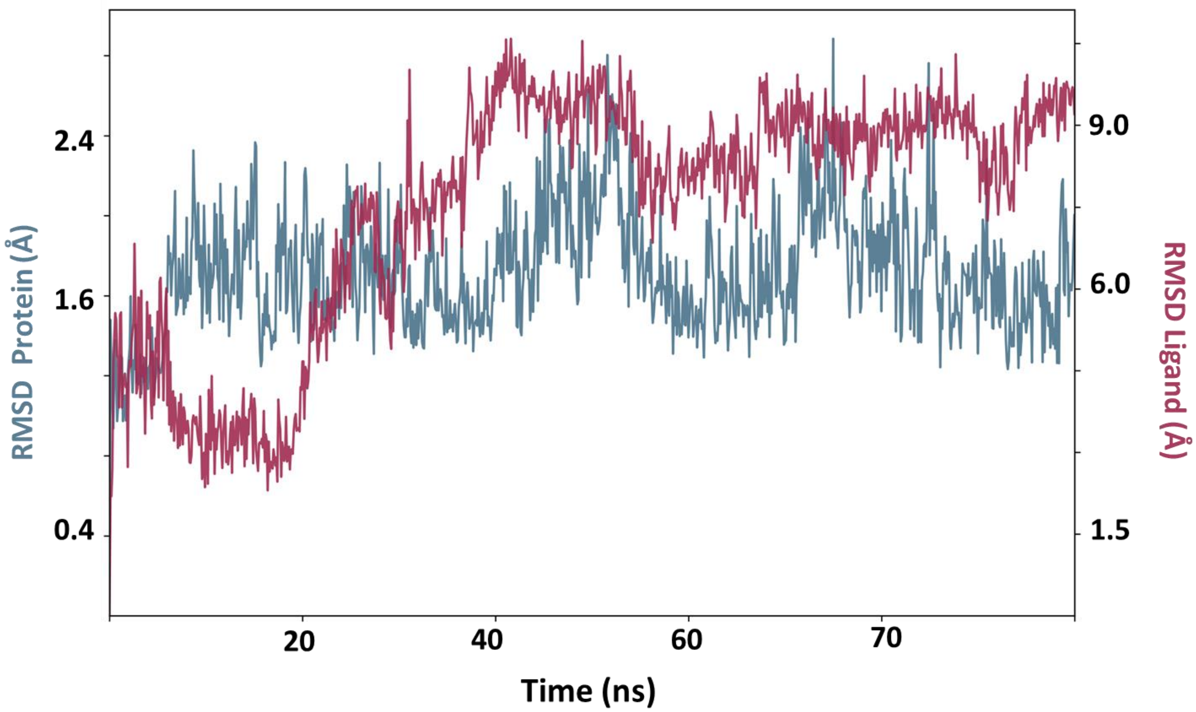

The RMSD of compound Pred15 in C111

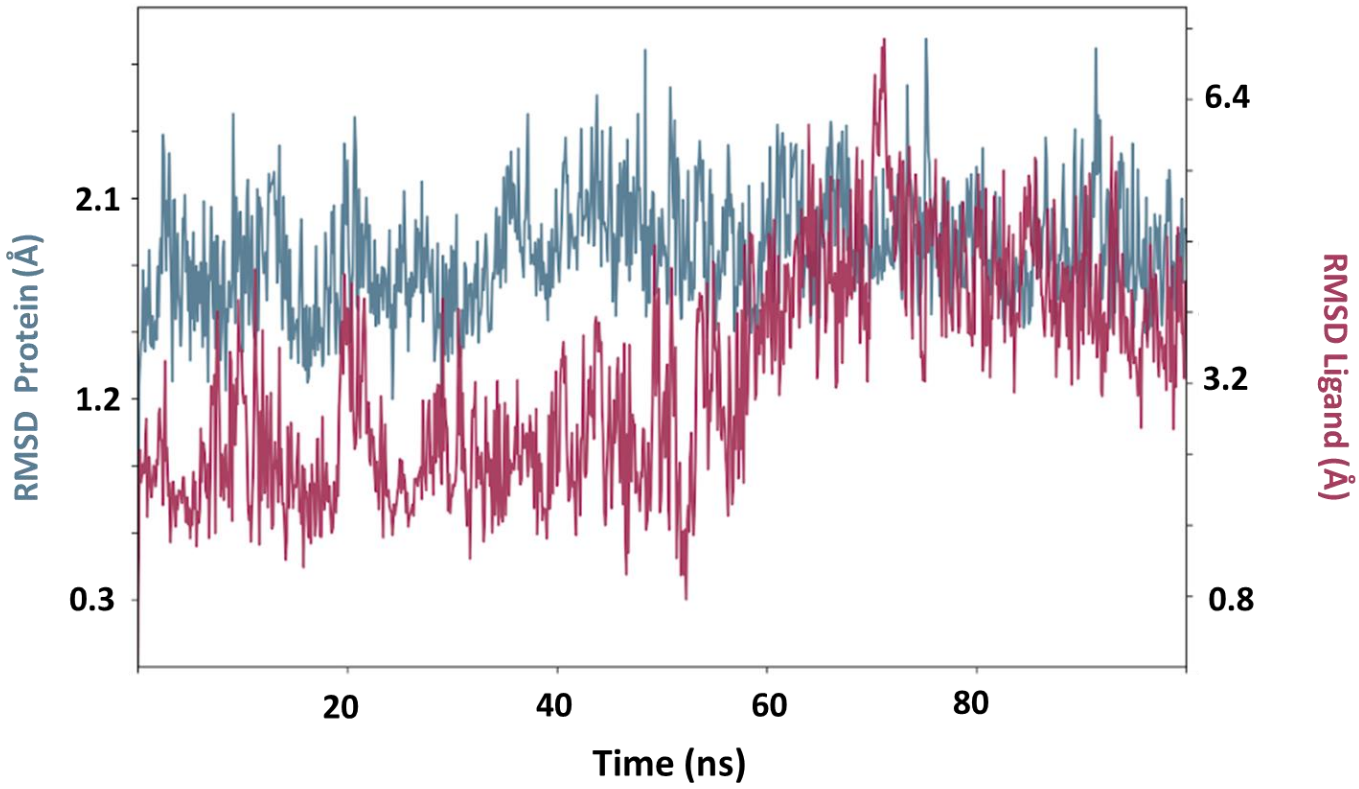

The RMSD of compound Pred14 in C111

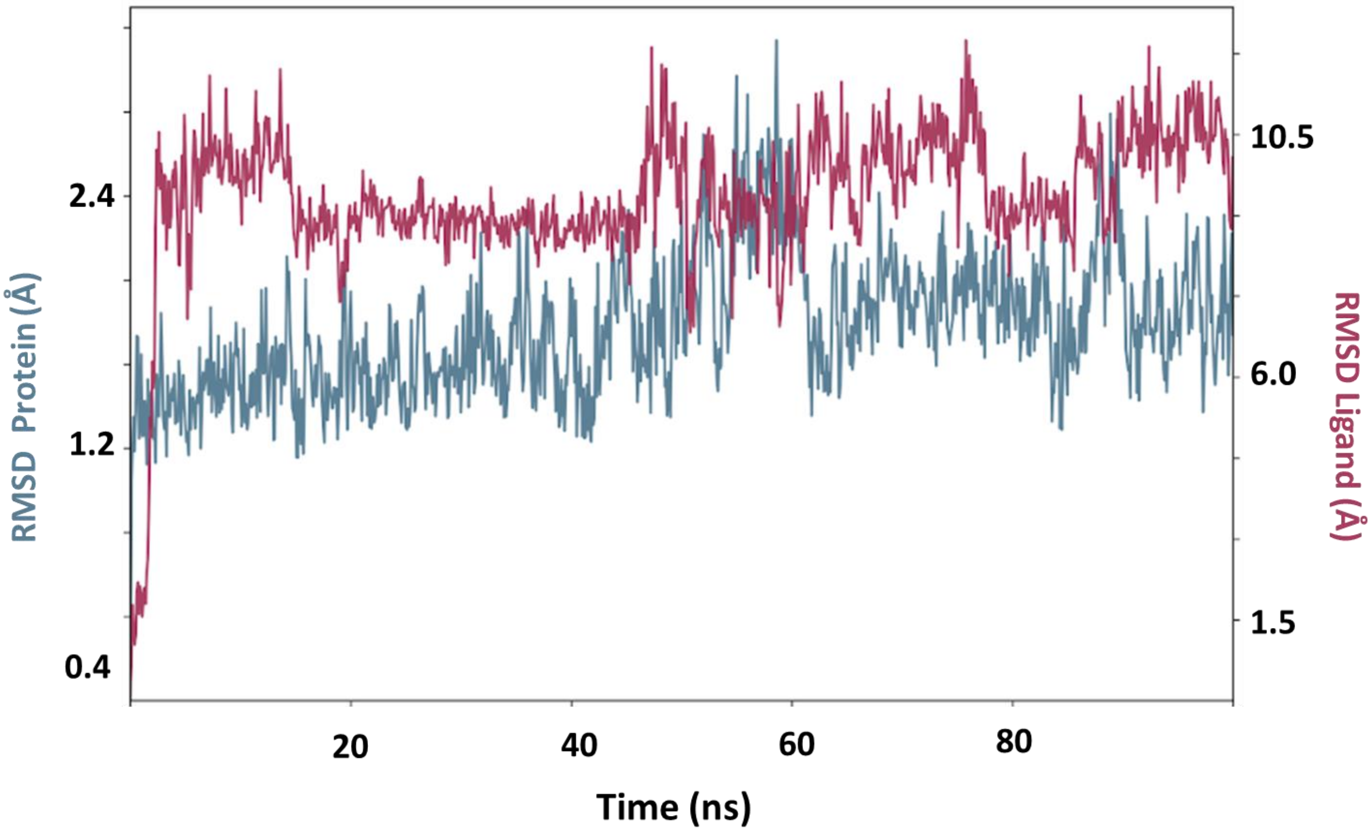

The RMSD of compound Pred13 in C111

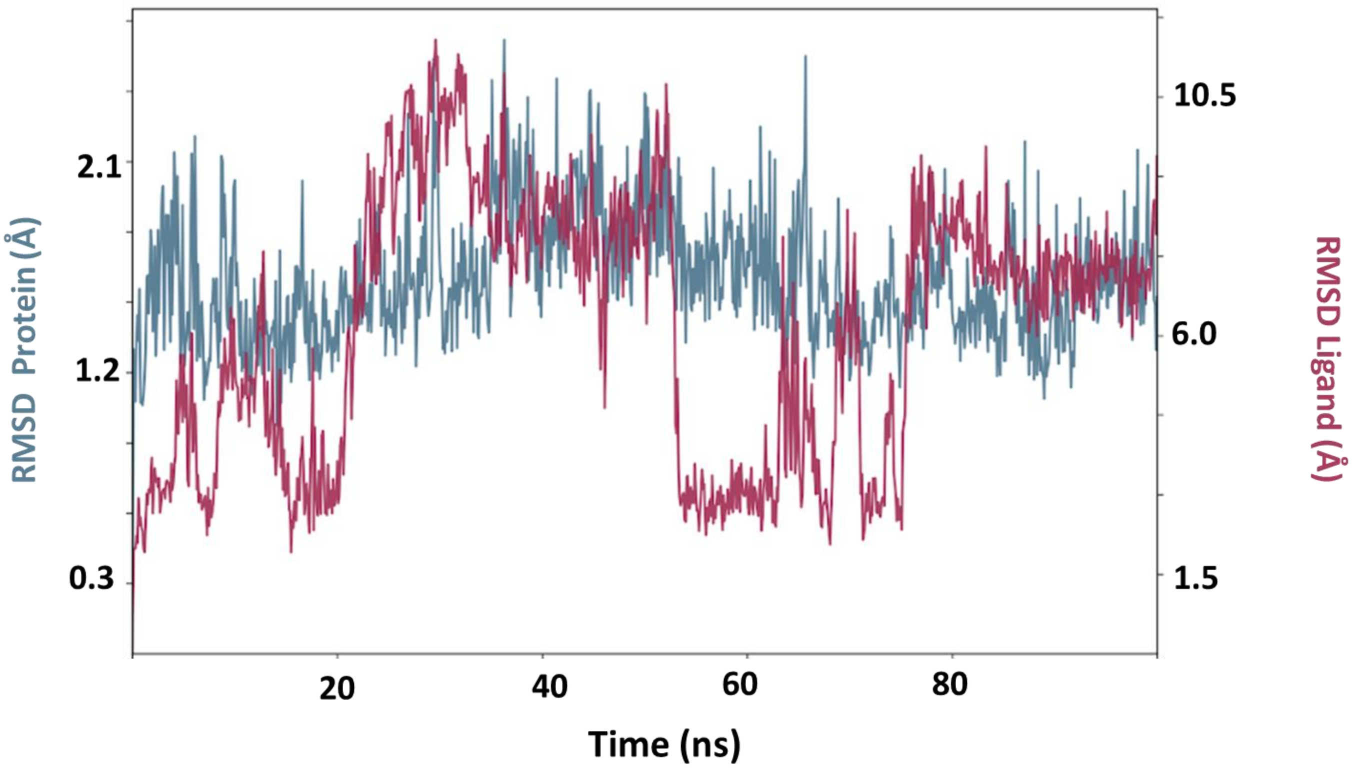

The RMSD of compound Pred6 in C111

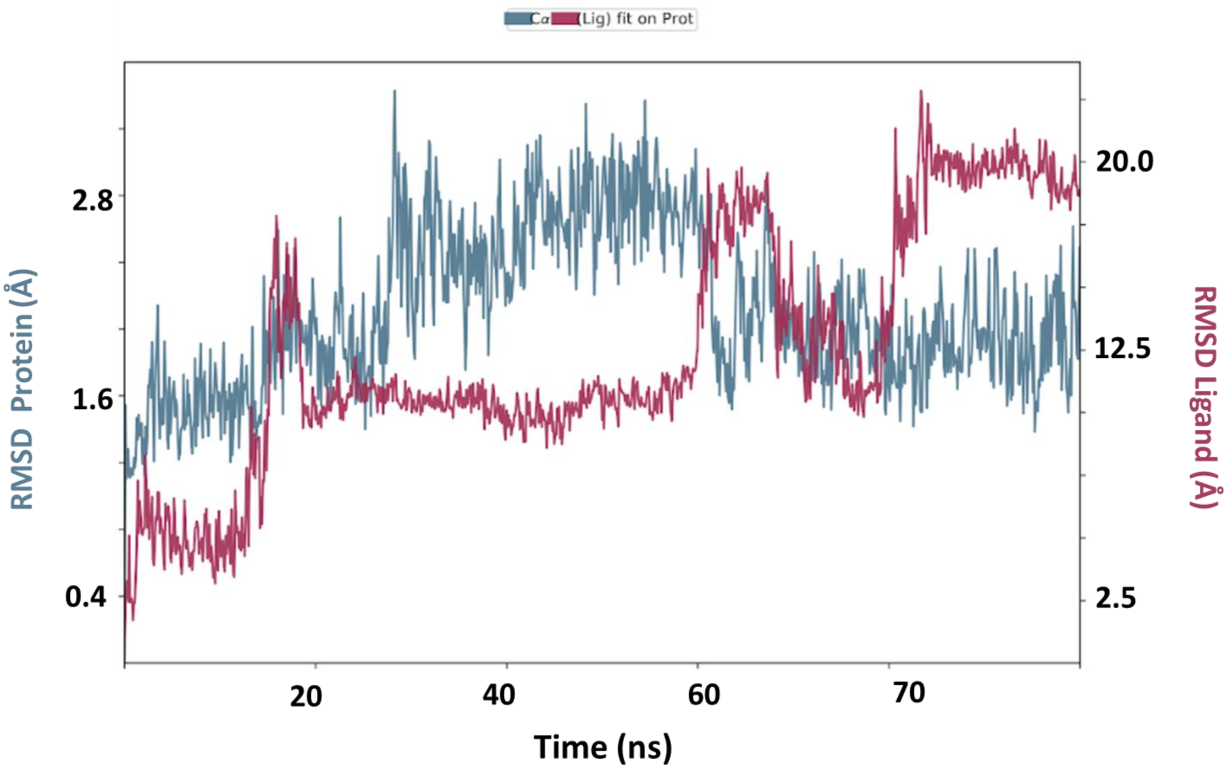

The RMSD of compound Pred14 in H73

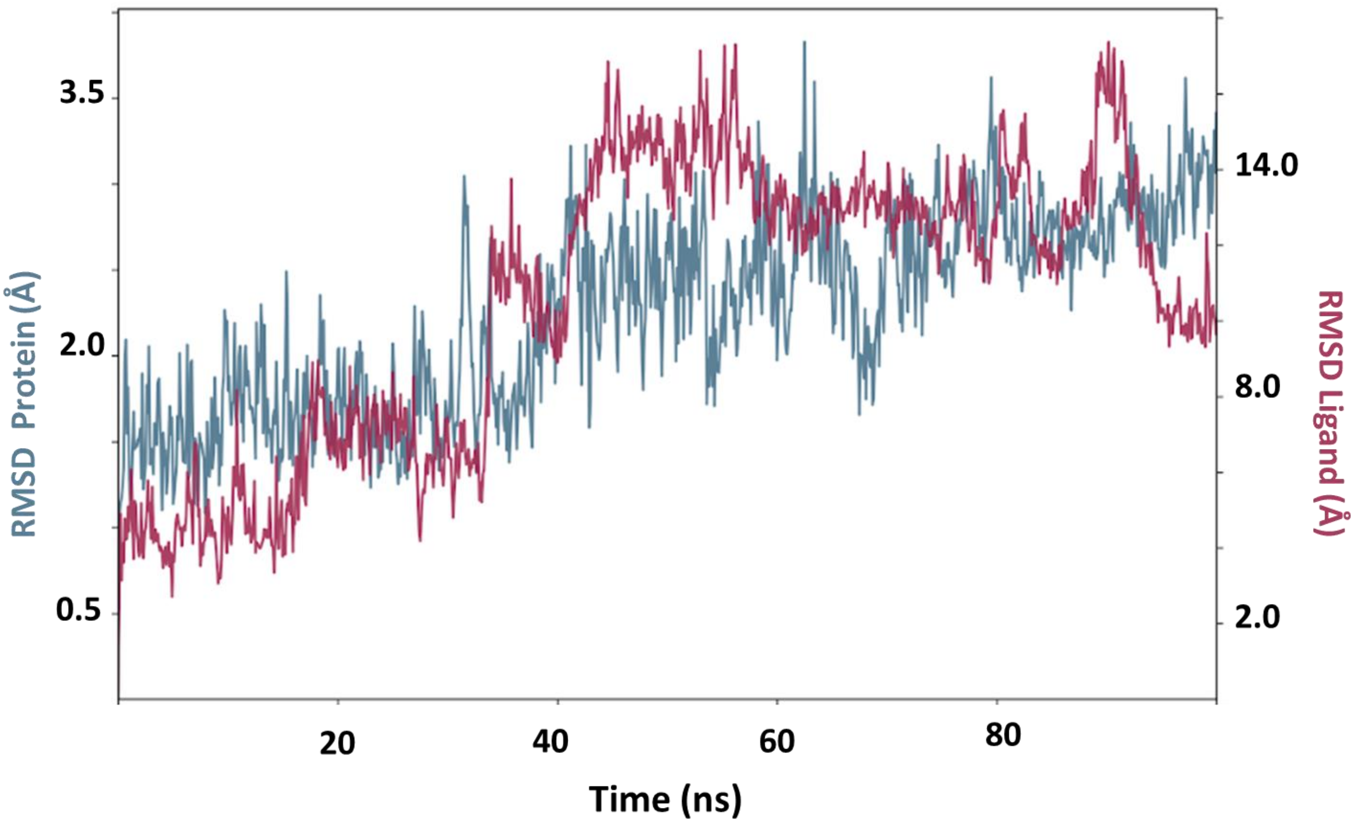

The RMSD of compound Pred10 in H73

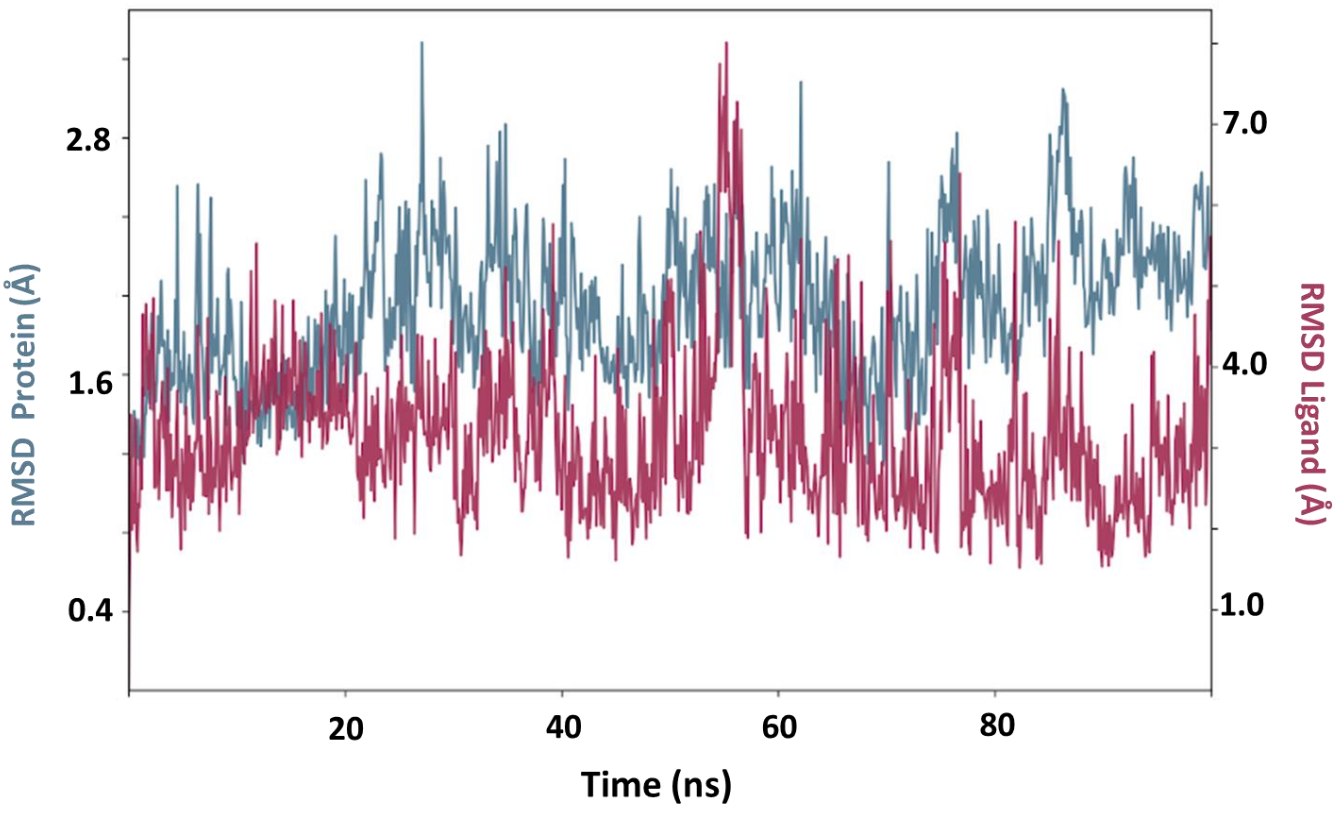

The RMSD of compound Pred15 in H73

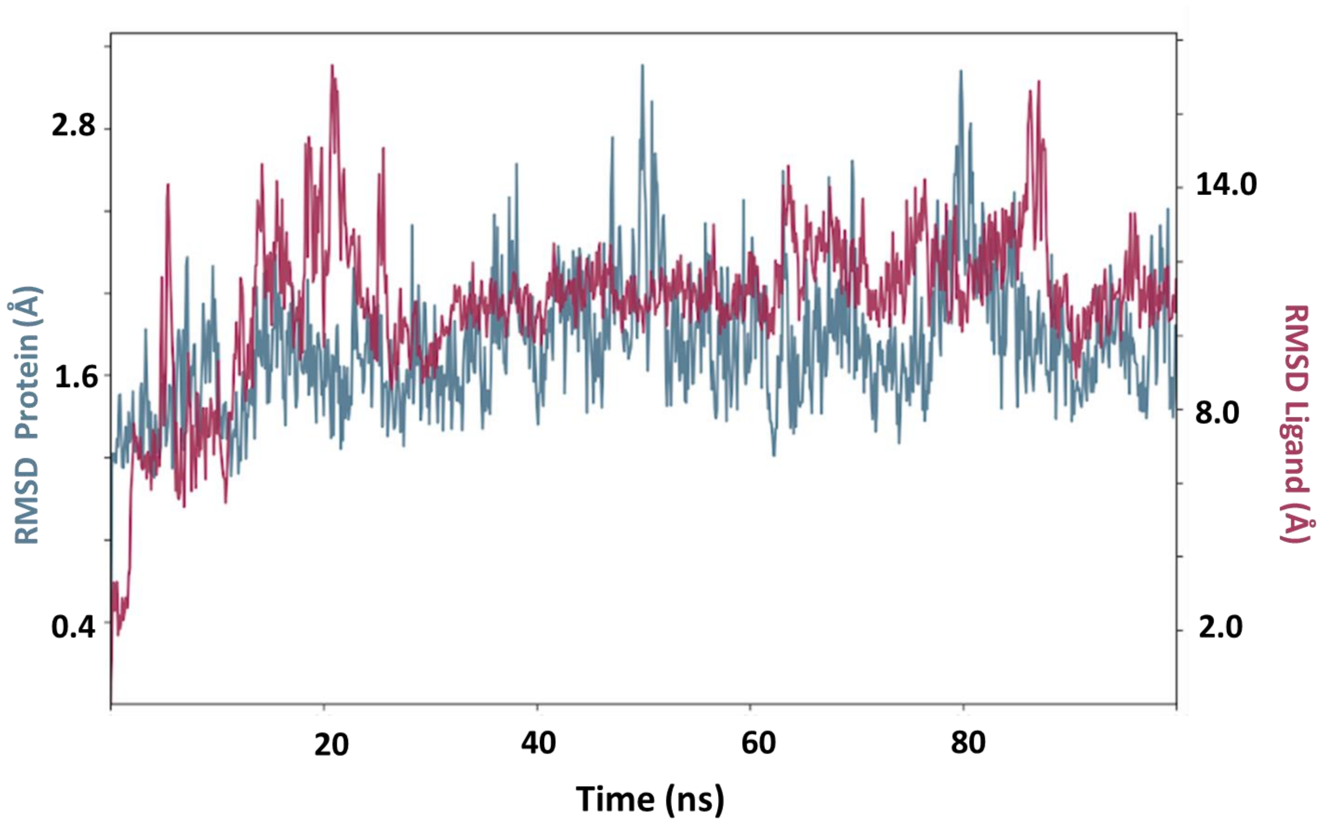

The RMSD of compound Pred2 in H73

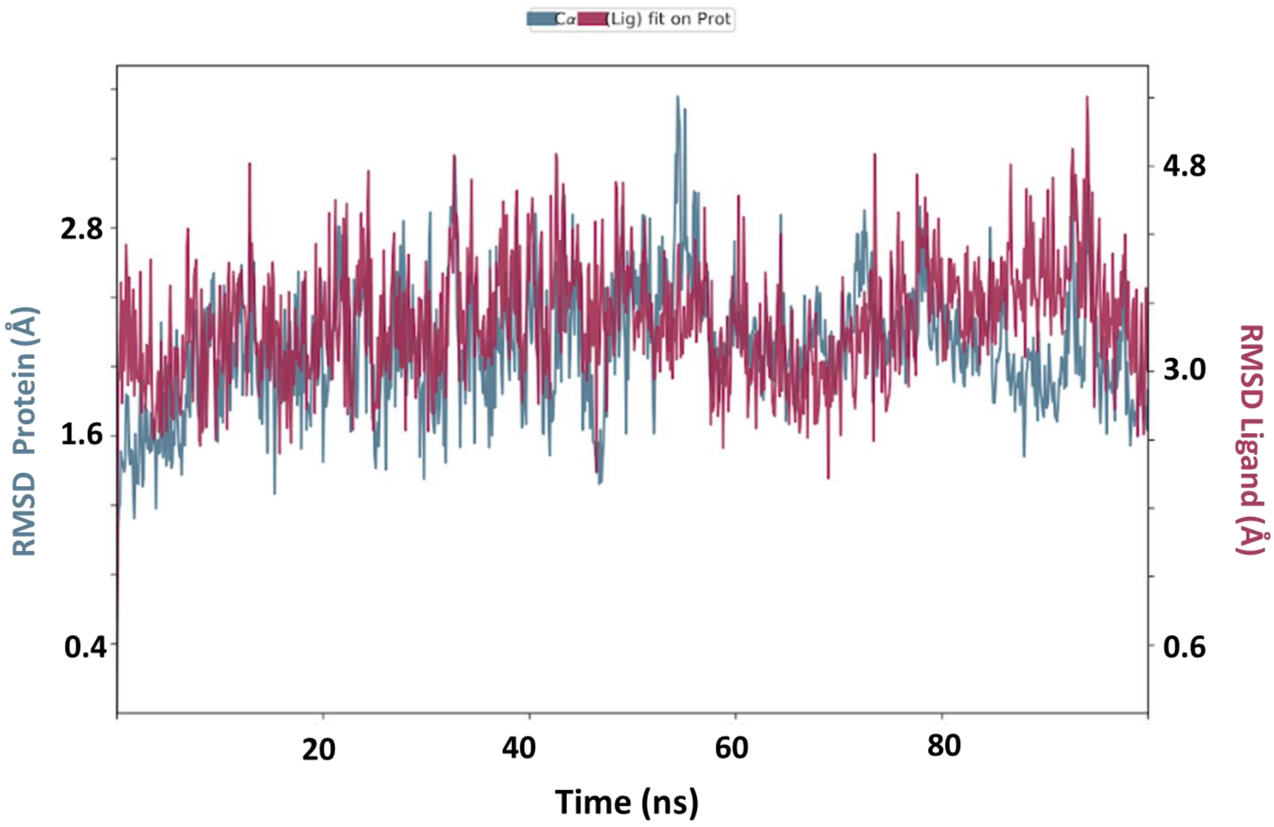

Supplement: Supplementary file 1 [file pharmaceuticals-17-00606-s001.zip › pharmaceuticals-2981564-supplementary.pdf]
